# Methylomic analysis of monozygotic twins discordant for autism spectrum disorder and related behavioural traits

**DOI:** 10.1038/mp.2013.41

**Published:** 2013-04-23

**Authors:** C C Y Wong, E L Meaburn, A Ronald, T S Price, A R Jeffries, L C Schalkwyk, R Plomin, J Mill

**Affiliations:** 1 King's College London, MRC Social, Genetic and Developmental Psychiatry Centre, Institute of Psychiatry, De Crespigny Park, London, UK; 2 Department of Psychological Sciences, Birkbeck, University of London, London, UK; 3 Institute of Translational Medicine and Therapeutics, School of Medicine, University of Pennsylvania, PA, USA; 4 University of Exeter Medical School, Exeter University, St Luke's Campus, Exeter, UK

**Keywords:** ASD, autism, copy-number variation, DNA methylation, epigenetics, monozygotic twins

## Abstract

Autism spectrum disorder (ASD) defines a group of common, complex neurodevelopmental disorders. Although the aetiology of ASD has a strong genetic component, there is considerable monozygotic (MZ) twin discordance indicating a role for non-genetic factors. Because MZ twins share an identical DNA sequence, disease-discordant MZ twin pairs provide an ideal model for examining the contribution of environmentally driven epigenetic factors in disease. We performed a genome-wide analysis of DNA methylation in a sample of 50 MZ twin pairs (100 individuals) sampled from a representative population cohort that included twins discordant and concordant for ASD, ASD-associated traits and no autistic phenotype. Within-twin and between-group analyses identified numerous differentially methylated regions associated with ASD. In addition, we report significant correlations between DNA methylation and quantitatively measured autistic trait scores across our sample cohort. This study represents the first systematic epigenomic analyses of MZ twins discordant for ASD and implicates a role for altered DNA methylation in autism.

## Introduction

Autism spectrum disorder (ASD) defines a collection of complex childhood neurodevelopmental disorders affecting ∼1% of the population and conferring severe lifelong disability.^1^ ASD is characterized by a triad of impairments: (1) deficits in social interactions and understanding, (2) non-social impairments, such as repetitive behaviour and interests, and (3) impairments in language and communication development. Quantitative genetic studies indicate that ASD has a strong heritable component,^2^ which is supported by the recent identification of several susceptibility loci and an emerging literature implicating the relevance of *de novo* and inherited copy number variants (CNVs) in the disorder.^3^ Despite intense research effort during the past decade, however, no definitive biological or clinical markers for ASD have been identified. This can be partly explained by the highly heterogeneous nature of ASD, both clinically and aetiologically. The clinical manifestation of ASD displays considerable individual variability in the severity of impairments and quantitative genetic studies also report genetic heterogeneity between the three trait domains of ASD.^4, 5, 6^

Despite the high heritability estimates for ASD, there is notable discordance within monozygotic (MZ) twin pairs for diagnosed ASD, and often considerable symptom severity differences within ASD-concordant MZ twins,^2^ strongly implicating a role for non-genetic epigenetic factors in aetiology. Epigenetic mechanisms mediate reversible changes in gene expression independent of DNA sequence variation, principally through alterations in DNA methylation and chromatin structure.^7^ Epigenetic changes in the brain have been associated with a range of neurological and cognitive processes, including neurogenesis,^8^ brain development^9^ and drug addiction.^10^ Emerging evidence implicates epigenetic modifications in several neuropsychiatric disorders, including ASD.^11, 12^ In particular, epigenetic dysregulation underlies the symptoms of Rett syndrome and Fragile X syndrome, two disorders with considerable phenotypic overlap with ASD.^11^ Although few empirical studies have systematically examined the role of altered epigenetic processes in ASD, recent analyses provide evidence for altered DNA methylation and histone modifications in disease pathology.^13, 14, 15^

The use of disease-discordant MZ twins represents a powerful strategy in epigenetic epidemiology because identical twins are matched for genotype, age, sex, maternal environment, population cohort effects and exposure to many shared environmental factors.^16, 17^ Recent studies have uncovered considerable epigenetic variation between MZ twins,^18, 19, 20^ and DNA methylation differences have been associated with MZ twin discordance for several complex phenotypic traits, including psychosis^21^ and Type 1 diabetes.^22^ In ASD, Nguyen and co-workers^23^ recently examined lymphoblastoid cell lines derived from peripheral blood lymphocytes collected from three ASD-discordant MZ twin pairs, reporting several ASD-associated differentially methylated loci.^23^ Two loci (*RORA* and *BCL2*) reported as hypermethylated in ASD were found to be downregulated in RNA from post-mortem autism brains. These findings support a role for DNA methylation in ASD and highlight the successful use of peripherally derived DNA from discordant MZ twins to identify disease-associated epigenetic changes. Given the highly heterogeneous nature of ASD, however, more comprehensive genome-wide analyses across larger numbers of samples are warranted to investigate the extent to which ASD-associated epigenetic variation is individual- and symptom-specific.

## Materials and methods

### Samples for methylomic analysis

Participants were recruited from the Twins’ Early Development Study (TEDS), a United Kingdom-based study of twins contacted from birth records.^24^ For this study, a total of 50 MZ twin pairs were identified within TEDS using the Childhood Autism Symptom Test (CAST), which assesses dimensional ASD traits, at age 8 years. The CAST^25^ is a 31-item screening measurement for ASD, designed for parents and teachers to complete in non-clinical settings to assess behaviours characteristic of the autistic spectrum. Items within the CAST are scored additively and a score of **⩾**15 (that is, answering ‘yes’ on **⩾**15 items) is the cutoff for identifying children ‘at risk’ for ASD. On the basis of the Diagnostic and Statistical Manual of Mental Disorders, Fourth Edition criteria for autism, CAST items can be divided into three subscales: impairments in social symptoms (12 items); impairments in non-social symptoms (that is, restricted repetitive behaviours and interests (RRBIs); 7 items); and communication impairments (12 items).^6^ The CAST has been widely used in population-based studies of singletons^25^ as well as in twin studies.^26^ Within TEDS, the CAST has been shown to have good reliability and validity.^27^ Supplementary Figure 1 shows the distribution of total CAST and its three subscale scores within samples selected using parent ratings. Supplementary Table 1 provides a summary of the samples included in the analyses. Whole-blood samples were collected from subjects at age 15 years by a trained phlebotomist for DNA extraction and blood cell-count analysis. Blood cell counts were assessed for all collected samples and found to be within the normal range.

### Genome-wide analysis of DNA methylation

For each individual, genomic DNA (500 ng) extracted from whole blood was treated with sodium bisulphite using the EZ 96-DNA methylation kit (Zymo Research, Irvine, CA, USA) following the manufacturer’s standard protocol. The bisulphite conversion reaction was performed in duplicate for each sample to minimize potential bias caused by variable conversion efficiency, and pooled bisulphite-treated DNA was used for subsequent array analysis. Genome-wide DNA methylation was assessed using the Illumina Infinium HumanMethylation27 BeadChip (Illumina, San Diego, CA, USA), which interrogates the DNA methylation profile of 27 578 CpG sites located in 14 495 protein-coding gene promoters and 110 microRNA gene promoters, at single-nucleotide resolution.^28^ Illumina GenomeStudio software (Illumina, San Diego, CA, USA) was used to extract signal intensities for each probe and perform initial quality control checks, with all data sets (except two individuals) being considered to be of high quality and included in subsequent analyses. To ensure stringent data quality, probes with a detection *P*-value >0.05 in any of the samples were removed across *all* individuals (*N*=1161 probes) in addition to a set of probes (*N*=2923) that were reported as nonspecific and potentially unreliable in a recent survey of all probes on the microarray.^29^

### Methylation microarray data processing

All computations and statistical analyses were performed within the R statistical analysis environment (http://www.r-project.org), and all analysis scripts are available on request from the authors. A customized pipeline was used for the analysis of Illumina 27K methylation data as described in a previous study of psychosis-discordant MZ twins.^21^ Briefly, signal intensities for each probe were normalized using quantile normalization to reduce unwanted interarray variation. The relative methylation level of each interrogated CpG site was calculated as the ratio of the normalized signal from the methylated probe to the sum of the normalized signals of the methylated and unmethylated probes. This gave an average DNA methylation value, described as average ‘*β*-value’ for each CpG site, ranging from 0 (unmethylated) to 1 (fully methylated). A density plot of *β*-values for every sample revealed that, as expected given the known distribution of probes on the array, the data followed a bimodal distribution (Supplementary Figure 2). An empirical variance stabilizing transformation was used to adjust for the bimodal distribution of the data.^21^ Raw microarray data are available for download from http://epigenetics.iop.kcl.ac.uk/ASDTwins/.

### Identification of ASD-associated DMRs

Two major analysis strategies were used to identify DMRs associated with ASD and related traits. First, DNA methylation differences within pairs of MZ twins were examined in MZ twin pairs discordant for ASD and ASD-related traits. Second, case–control comparisons of DNA methylation were performed between groups of individuals scoring high and low for ASD traits. With the aim of identifying real, biologically relevant within-twin and between-group DNA methylation differences, we used an analytic approach that incorporates both the significance (that is, *t*-test statistic) and magnitude (that is, absolute delta-*β* (Δ*β*)) of any observed differences to produce a ranked list of DMRs.^21^ A summary of the analysis strategy is presented in Supplementary Figure 3. This combined approach, where data are interpreted based on the combination of fold change and statistical significance, is routinely used in genome-wide gene expression studies and has been shown to produce gene lists of higher reproducibility and biological relevance.^30^ We recently used a similar approach successfully to identify disease-associated epigenetic changes in a psychosis-discordant MZ twin study.^21^ Given the known phenotypic and aetiologic heterogeneity, we also screened for large Δ*β*-values within each discordant MZ twin pair to examine the possibility that disease-associated epigenetic changes are potentially private and not consistent across all families. Finally, we examined whether quantitative CAST scores are correlated with DNA methylation at specific loci. The association between each of the quantitatively rated CAST subscale variables and DNA methylation at each CpG site was assessed using Pearson’s product–moment correlation.

### Global DNA methylation analysis

Global levels of DNA methylation were quantified using the LUminometric Methylation Assay (LUMA).^31^ This method relies on DNA cleavage by methylation-sensitive and -insensitive restriction enzymes, followed by the quantification of the resulting restriction fragments using pyrosequencing.^31^ Positive controls, including both artificially methylated and artificially unmethylated samples, were included in all experimental steps to ensure unambiguous restriction enzyme digestions and to calibrate the experimental data, with each sample being processed in duplicate.

### Fine mapping of DNA methylation using bisulphite pyrosequencing

Although the Illumina 27K array has been well validated for detecting differences in DNA methylation, we further tested specific regions nominated from the genome-wide microarray analysis using bisulphite pyrosequencing. Independent verification analyses were performed on two CpG sites (cg16474696, *MGC3207*; cg20507276, *OR2L13*) that demonstrated a large significant ASD-associated difference from the case versus control analysis. In each case, the assay spanned multiple CpG sites, including the specific CpG interrogated on the Illumina 27K array. Briefly, 500 ng DNA from each individual was independently treated with sodium bisulphite in duplicate using the EZ 96-DNA methylation kit as described above. Bisulphite-polymerase chain reaction amplification was performed in duplicate. Quantitative DNA methylation analysis was conducted using the PyroMark Q24 pyrosequencer (Qiagen, Valencia, CA, USA). The correlation between DNA methylation estimates obtained from Illumina 27K array and bisulphite pyrosequencing was assessed using Pearson’s moment–correlation coefficient. In addition, Sanger sequencing using BigDye v.3.1 terminator mix (Applied Biosystems, Foster City, CA, USA) was performed on the regions targeted by the *MGC3207* pyrosequencing assay to ensure that the Illumina probe sequences and the primer binding sites for the pyrosequencing assay were free of any DNA sequence variation. The primers and assay conditions are given in Supplementary Table 2.

### CNV analysis using genotyping arrays

Genomic DNA (200 ng) extracted from whole blood was genotyped using the Illumina HumanOmniExpress BeadChip (Illumina) targeting >730 000 single-nucleotide polymorphisms and Illumina GenomeStudio software was used to call genotypes based on predefined genotype cluster boundaries to denote cluster positions (HumanOmniExpress-12v1_C.egt). CNVs were identified from the genotyping data using two independent algorithms, PennCNV^32^ and QuantiSNP,^33^ with default parameters, and GC content signal preprocessing was applied. Stringent quality control steps were used to ensure that only high-confidence CNVs, that is, those >1 kb in size, covered by >5 probes and detected by both programs, were included for further analysis.

## Results

### ASD is not associated with systemic differences in global DNA methylation

As expected, within-twin patterns of DNA methylation were highly correlated across all MZ twin pairs (average within-twin *r* across all probes=0.99), indicating that ASD and related traits are not associated with systemic changes in epigenetic programming. Supplementary Figure 4a shows the correlation between genome-wide DNA methylation across all probes on the array and one example ASD-discordant MZ twin pair; data for the other ASD-discordant MZ pairs are available for download from http://epigenetics.iop.kcl.ac.uk/ASDTwins/. These data were corroborated by global DNA methylation analysis using LUMA, which identified no significant difference between affected ASD twins and their co-twins (affected ASD twins mean=65.1%, unaffected co-twins mean=65.9% *P*=0.817) (Supplementary Figure 4b).

### Site-specific DNA differences are widespread in MZ-discordant ASD twins

**Figure 1:**
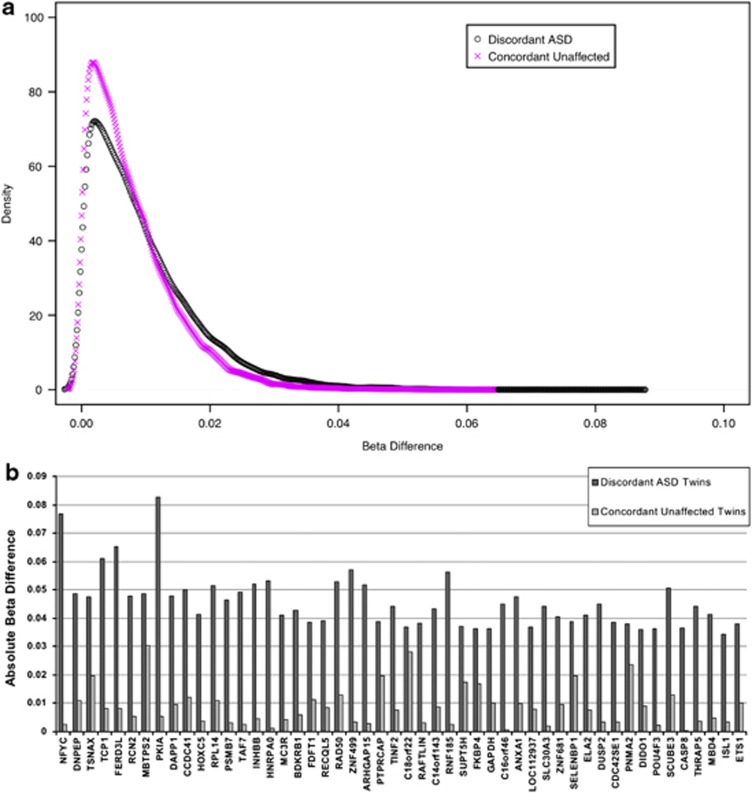
(**a**) A significantly (*P*<2.2E−16) higher number of CpG sites with a large average within-twin *β* differences was observed in autism spectrum disorder (ASD)-discordant monozygotic (MZ) twin pairs compared with unaffected twin pairs (that is, concordant for low Childhood Autism Symptom Test (CAST) score). (**b**) Absolute mean Δ*β* of the top 50 differentially methylated CpG sites in ASD-discordant MZ twin pairs and in unaffected MZ twin pairs.

**Figure 2:**
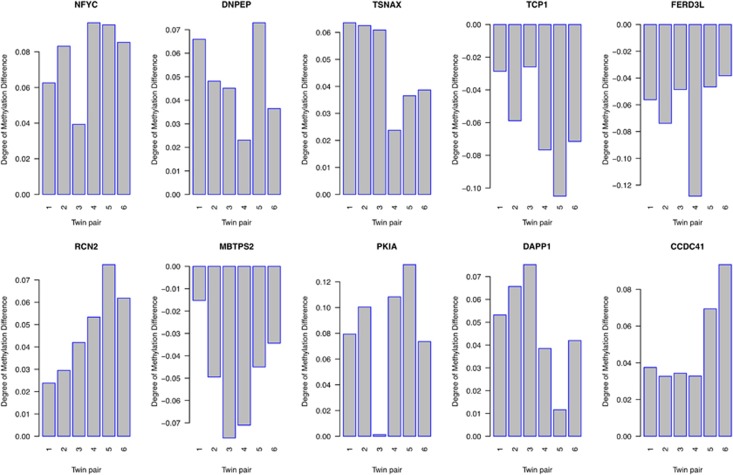
DNA methylation differences (Δ*β*; autism spectrum disorder (ASD) twin minus well twin) for the top 10 ASD-associated differentially methylated CpG sites in six ASD-discordant monozygotic (MZ) twin pairs.

In contrast to global levels of DNA methylation, DNA methylation at individual CpG sites demonstrated considerable variability within ASD-discordant MZ twin pairs. Figure 1a shows the distribution of average absolute differences in DNA methylation (Δ*β*) within all MZ twins discordant for ASD and ‘control’ MZ twin pairs concordant for low autistic trait score (unaffected). The overall distribution of average within-pair DNA methylation differences showed a highly significant skew to the right in ASD-discordant twins (*P*<2.2e−16, Kolmogorov–Smirnov test), with a higher number of CpG sites demonstrating a larger average difference in DNA methylation. Using an analysis method designed to identify the largest and most significant differences in DNA methylation at individual CpG sites, we identified multiple CpG sites across the genome exhibiting significant ASD-associated differential DNA methylation (Table 1). Of note, variability at these sites appears to be specific to ASD-discordant twin pairs; for the 50 top-ranked ASD-associated DMRs, we observe significantly higher average within-pair differences for MZ twin pairs discordant for ASD (*P*<0.01; see Figure 1b). The top differentially methylated site (cg13735974) across all ASD-discordant MZ twin pairs located in the *NFYC* promoter was consistently hypermethylated in affected individuals compared with their unaffected co-twins (mean Δ*β*=0.08, range=0.04–0.10, *P*<0.0004). For the top 10 DMRs, Figure 2 indicates highly consistent differences across all six ASD-discordant twin pairs.

### Large DNA methylation differences are observed at specific loci within individual ASD-discordant MZ twin pairs

Because ASD is a highly heterogeneous disorder,^3^ it is probable that many disease-associated DMRs are family-specific. We therefore screened for the largest family-specific DNA methylation differences within each discordant ASD twin pair, identifying numerous loci (average=37.4 per twin pair) showing large DNA methylation differences (Δ*β***⩾**0.15) within each discordant twin pair (Supplementary Figure 5 and Supplementary Table 3). Although the majority of DMRs of large magnitude are family-specific, several are common across two or more discordant twin pairs in the same direction: cg12164282, located in *PXDN* promoter, showed ASD hypomethylation in twin pair 2 (Δ*β*=−0.19) and twin pair 4 (Δ*β*=−0.28); cg04545708, located in exon 1 of *C11orf1*, showed ASD hypermethylation in both twin pair 3 (Δ*β*=0.23) and twin pair 6 (Δ*β*=0.35); cg20426860, located in exon 1 of *TMEM161A*, showed ASD hypermethylation in twin pair 4 (Δ*β*=0.21) and twin pair 6 (Δ*β*=0.27); and cg27009703, located in *HOXA9* promoter, showed ASD hypermethylation in twin pair 1 (Δ*β*=0.19) and twin pair 4 (Δ*β*=0.21).

### DNA methylation differences are observed in MZ twins discordant for ASD-related traits

We detected significant DNA methylation differences between MZ twin pairs discordant for the three ASD-associated traits: that is, social autistic traits (*N*=9 MZ pairs), autistic RRBIs (*N*=9 MZ pairs) and communication autistic traits (*N*=8 MZ pairs). The top-ranked DMRs for each trait are shown in Supplementary Figure 6 and Supplementary Table 4. Interestingly, these included several genes previously implicated in the aetiology of ASD, including *GABRB3*, *AFF2*, *NLGN2*, *JMJD1C*, *SNRPN*, *SNURF*, *UBE3A* and *KCNJ10.*

As ASD is composed of a triad of all three impairments, we also examined if any CpG sites are differentially methylated across all discordant twin pairs (*N*=32 pairs, 64 individuals), regardless of their focal impairment. The top DMRs across all discordant twin pairs are shown in Supplementary Figure 7 and Supplementary Table 5. The top-ranked DMR located in the promoter region of *PIK3C3* (cg19837131) was significantly hypomethylated in affected individuals compared with their unaffected co-twins (mean Δ*β*=−0.04, *P*<0.00004). Interestingly, while the overall *average* difference at this locus is small, the range of within-twin methylation difference is much greater (Δ*β* ranges from −0.12 to 0.6) and that the direction of effect is strikingly consistent across the majority of individual twin pairs, with 25 out of 32 discordant pairs (78%) demonstrating trait-related hypomethylation (Supplementary Figure 7).

### Between-group analyses identified additional ASD-associated DMRs

Our study design also permitted us to examine group-level DNA methylation differences between ASD cases and controls. Unlike the within-pair discordant MZ twin design, between-group DNA methylation differences can be attributable to both genetic and environmental factors. Given the known gender difference in DNA methylation across the X chromosome, these analyses were restricted to probes on the autosomes (*N*=22 678) to minimize gender-induced biases.

Numerous DNA methylation differences were observed between ASD cases and controls. Supplementary Table 6a and Supplementary Figure 8 highlight the CpG sites showing the largest absolute DNA methylation differences (mean Δ*β***⩾**0.15) between ASD cases and unrelated control samples. The top case–control ASD-associated DMR was located upstream of *MGC3207* (cg16474696), which was significantly hypomethylated in ASD cases compared with control samples (mean Δ*β*=−0.24, *P*<0.0002). In addition to *MGC3207*, large significant ASD-associated differences were observed in several other loci, including CpG sites near *OR2L13* (cg20507276; mean Δ*β*=0.18) and *C14orf152* (cg20022541; mean Δ*β*=−0.16, data not shown). Verification experiments were conducted on *MGC3207* and *OR2L13* using bisulphite-pyrosequencing confirming a high correlation (*r*=0.91 and 0.86, for *MGC3207* (total *N*=33) and *OR2L13* (total *N*=35), respectively) in DNA methylation levels, detected using the Infinium microarray and pyrosequencing platforms. Although our list of DMRs was stringently filtered to exclude probes containing known polymorphic SNPs,^29^ several of the top-ranked case–control DMRs, including cg16474696 and cg20507276, demonstrated patterns of DNA methylation consistent with DNA sequence effects, suggesting that they may be mediated by *cis* effects on DNA methylation^34^ or potentially reflect technical artefacts caused by uncatalogued sequence variation in probe binding sequences. To exclude the latter for *MGC3207*, we sequenced genomic DNA across the DMR in a range of samples showing differential methylation and identified no obvious polymorphic DNA sequence variation in the immediate vicinity of the probe.

### Epigenetic differences identified between sporadic and familial ASD cases

ASD is an aetiologically heterogeneous syndrome and can occur both as a sporadic and a familial disorder. Recent CNV analyses report considerably higher frequencies of *de novo* variation in simplex compared with multiplex ASD families,^35^ suggesting that they represent genetically distinct classes. To test whether these are epigenetically distinct, we compared DNA methylation between individuals with sporadic ASD (where ASD is reported in only one member of the MZ twin pair; *N*=6) and individuals with familial ASD (as observed in concordant ASD MZ twin pairs; *N*=10). The genes most proximal to the 50 top-ranked differentially methylated CpG sites from this analysis are listed in Supplementary Table 6b. The top differentially methylated CpG site (cg07665060) is located upstream of *C19orf33*, which was significantly hypomethylated in individuals affected by sporadic ASD compared with those affected by familial ASD (mean Δ*β*=−0.12, *P*<0.0008) (Supplementary Figure 9). Interestingly, significant DNA methylation differences were also observed near several genes that have been previously implicated in ASD, including *MBD4*, *AUTS2* and *MAP2*.

### There is some overlap in DMRs across analytical groups

Table 2 provides a full list of top-ranked DMRs demonstrating overlap between analytical groups and highlighting their potential relevance to different autism-associated phenotypes. Interestingly, the top-ranked locus from the ASD-discordant twin analysis, located near *NFYC*, was also differentially methylated in the case–control analysis (mean Δ*β*=0.04, *P*<0.003). Furthermore, we identified significant DNA methylation differences in the *MBD4* promoter in both ASD-discordant twin analysis and sporadic *versus* familial ASD analysis, suggesting that *MBD4* methylation may have functional relevance to sporadic ASD. For each of the 50 top-ranked probes in each analysis category, Supplementary Table 7 lists their corresponding rank across the other analysis groups; although there is some overlap across groups (and each ranked list is positively, although modestly, correlated; Supplementary Table 8), few CpG sites are consistently altered across multiple analytical groups.

### Quantitative autistic trait scores are correlated with DNA methylation at multiple CpG sites

**Figure 3:**
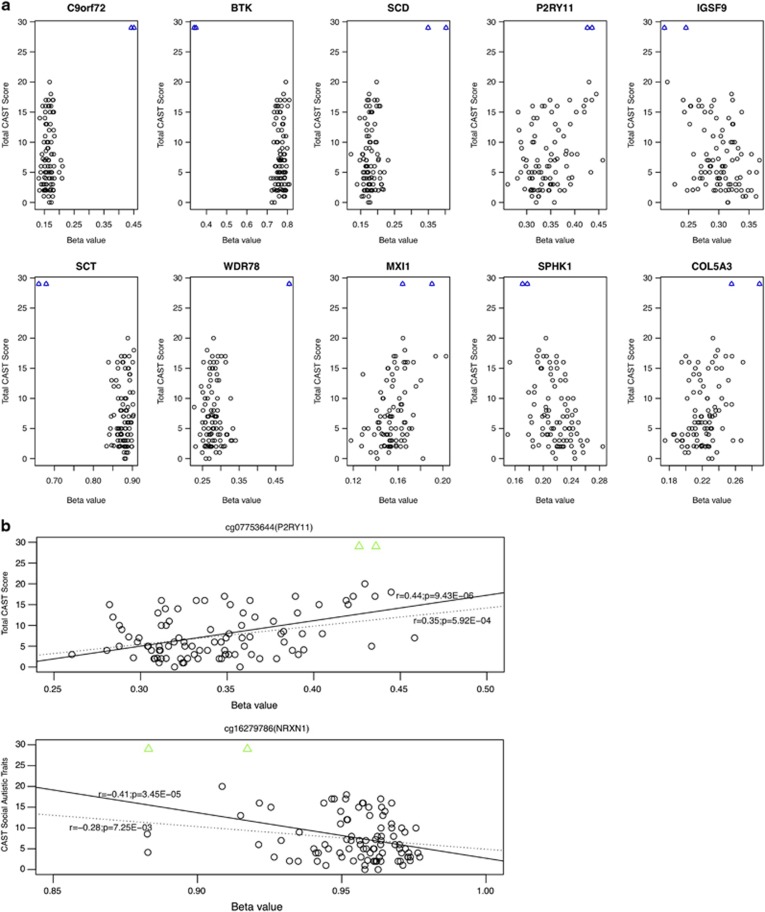
(**a**) The top 10 CpG sites showing the most significant correlation with total Childhood Autism Symptom Test (CAST) score. Each circle represents a sample. For some loci, the high correlations are influenced by extreme DNA methylation and CAST scores from a single pair of autism spectrum disorder (ASD)-concordant twins (denoted as blue triangles). (**b**) Significant correlation between *P2RY11* and *NRXN1* DNA methylation and quantitative autistic trait scores remains when the extreme twin pair was excluded. Solid and dashed lines represent results from correlation analysis including and excluding the extreme twin pair, respectively.

Supplementary Figure 1 shows the distribution of total CAST and its three trait subscale scores across our samples. Initial analyses highlighted a strong correlation between DNA methylation and CAST score at multiple CpG sites (Supplementary Table 9). Further analysis showed that many of these correlations are influenced by extreme DNA methylation levels and phenotypic scores exhibited by one male ASD-concordant MZ twin pair (Figure 3a and Supplementary Figure 10). These twins are extreme outliers for CAST score (both scored 29 out of a maximum score of 31) and DNA methylation at multiple CpG sites (Supplementary Figure 11), and both have a history of pervasive developmental problems, with severe behavioural phenotypes and early-appearing IQ deficits, with special deficits in language. Given the existing link between highly penetrant CNVs and severe ASD, we tested whether the extreme patterns of DNA methylation in these two twins were associated with the presence of genomic alterations. Interestingly, high-density SNP microarray analysis revealed significant structural genomic alterations at multiple loci, with CNVs detected in regions previously implicated in ASD (Supplementary Table 10).

DNA methylation at multiple CpG sites remained significantly correlated with CAST scores even after this extreme twin pair was excluded from analyses (Supplementary Table 11 and Figure 3b), suggesting that they do not necessarily represent epigenetic/phenotypic ‘outliers’ but have DNA methylation levels (and phenotypic scores) at the extreme end of a true quantitative spectrum. For example, there is a strong correlation between DNA methylation at cg07753644 in *P2RY11* and total CAST score in both analyses (with extreme twin pair: *r*=0.44; *P*=0.000009; without extreme twin pair: *r*=0.35; *P*=0.0006). Furthermore, DNA methylation at cg16279786 in the known ASD susceptibility locus, *NRXN1*, is significantly correlated with social autistic trait score in both analyses with (*r*=−0.41; *P*=0.00003) and without (*r*=−0.28; *P*=0.007) the extreme twin pair.

## Discussion

This study represents the first comprehensive analysis of DNA methylation differences in MZ twins discordant for ASD and autism-related traits using a genome-wide approach. We report ASD-associated DNA methylation differences at numerous CpG sites, with some DMRs consistent across all discordant twin pairs for each diagnostic category and others specific to one or two twin pairs, or one or two autism-related traits. Although sporadic cases of ASD appear to be epigenetically distinct to familial cases of ASD, some DMRs are common across both discordant MZ twin and case–control analyses. We also observed that DNA methylation at multiple CpG sites was significantly correlated with quantitatively rated autistic trait scores, with our analyses identifying one MZ twin pair, concordant for a very severe autistic phenotype, that appear to represent epigenetic outliers at multiple CpG sites across the genome. Interestingly, both individuals harbour numerous CNVs in genomic regions previously implicated in autism. Given the important role of epigenetic mechanisms in regulating gene expression, it is plausible that, like CNVs, methylomic variation could mediate disease susceptibility via altered gene dosage. Our hypothesis-free experimental design allowed us to identify disease-associated DNA methylation differences at loci not previously implicated in ASD, although we also found evidence for epigenetic changes at several genes previously implicated in autism.

Our findings have several implications for our understanding about the aetiology of ASD. First, they document the presence of numerous DNA methylation differences in MZ twins discordant for ASD and ASD-related traits, as well as between autistic individuals and control samples. This concurs with findings from a previous ASD-discordant twin study^23^ and further supports the association of variable DNA methylation with phenotypic differences between genetically identical individuals.^21, 22^ Second, the observed DNA methylation differences in MZ twins discordant for ASD and ASD-related traits, who are otherwise matched for genotype, shared environment, age, sex and other potential confounders, highlight the role of non-shared environmental and stochastic factors in the aetiology of autism. These findings concur with mounting data suggesting that environmentally mediated effects on the epigenome may be relatively common and important for disease.^36^ Third, our data suggest that although DNA methylation at some CpG sites is consistently altered across the entire set of discordant twins, differences at other CpG sites are specific to certain symptom groups, with considerable overall epigenetic heterogeneity between the three domains of autistic traits. These findings are in line with recent genetic research demonstrating significant genetic heterogeneity between the three core symptoms of ASD.^4, 6^ Fourth, the analysis of individual ASD-discordant twin pairs suggests that there is also considerable familial heterogeneity, with rare epigenetic alterations of large magnitude being potentially associated with ASD. These findings are not entirely surprising given the known heterogeneous nature of ASD revealed by molecular genetic studies,^3^ with an important role for highly penetrant rare genomic alterations, especially *de novo* mutations. Fifth, the identification of significant correlations between DNA methylation and autism symptom scores across our sample cohort suggests that there is a quantitative relationship between the severity of the autistic phenotype and epigenetic variation at certain loci. This reinforces the view of autism as the quantitative extreme of a phenotypic spectrum and highlights the potential use of epigenetic biomarkers as a predictor for severity of symptoms, although the accuracy, sensitivity and specificity of such predictors would require extended investigation. Finally, in addition to implicating a number of novel genes in the aetiology of ASD, we identified ASD-associated differential DNA methylation in the vicinity of multiple loci previously implicated in the pathogenesis of autism in genetic studies, including *AFF2*, *AUTS2*, *GABRB3*, *NLGN3*, *NRXN1*, *SLC6A4* and *UBE3A* (see Supplementary Table 12 for a comprehensive list).

This study has several strengths. First, our unique sample consisted of MZ twin pairs discordant for autism and ASD-related-traits, in addition to age-matched concordant MZ twin pairs (for both ASD and low CAST score). It allowed us to perform a comprehensive analysis of the role of DNA methylation in ASD and ASD-related traits controlling for genotype, age, sex and other potential confounders. Second, by undertaking a genome-wide approach using a robust and reliable array platform, we were able to uncover phenotype-relevant differentially methylated loci in genomic regions that are both novel and have been previously associated with ASD. Third, our analysis of 32 discordant MZ twin pairs is relatively large compared with other discordant twin studies performed for other complex disease phenotypes; in this regard, for example, the only other ASD-discordant twin study assessed only three MZ twin pairs.^23^ Finally, we were able to complement our discordant-twin analyses by assessing group-level differences between ASD cases and controls, and also examining the relationship between DNA methylation and quantitatively rated trait scores across our entire sample cohort.

This study also has a number of limitations that should be considered when interpreting the results. First, although this is the largest and most comprehensive study of epigenetic variation in ASD performed to date, the sample size for each subgroup is small, in part because truly discordant MZ twin pairs are relatively rare. Although none of the reported differentially methylated loci reached a Bonferroni-corrected *P*-value cutoff (*P*=2.13E–05 for discordant-twin analysis and *P*=2.20E–05 for between-group analysis), this statistical approach is likely to be too conservative, especially given the non-independence of CpG sites^37^ and the small numbers of samples tested in each group. In this study, a combined analytic approach, taking into account the significance and the extent of methylation change, was used to identify differentially methylated loci that have potentially real, biological relevance to ASD. This analytic approach is widely used in genome-wide gene expression studies and is reported to produce gene lists of higher reproducibility and biological relevance compared with the convention method that relies solely on statistical significance.^30^ This notion is supported by the identification of differentially methylated loci near numerous genes previously implicated in ASD. Nonetheless, given the relatively small subgroup sample size, replication in larger samples is needed. Second, genome-wide DNA methylation profiling was performed on DNA extracted from whole blood, controlled for cell count, rather than the brain. Unfortunately, there is no archived collection of post-mortem brain samples from ASD-discordant MZ twins. Although there are known tissue-specific differences in DNA methylation profiles, recent studies suggest that disease-associated epimutations may be detectable across tissues,^38^ and our recent work suggests that some between-individual epigenetic variation is conserved across brain and blood.^39^ Furthermore, ASD-associated epimutations have been demonstrated to be detectable both in the brain and in peripheral tissues (that is, blood).^13, 23^ Moreover, our identification of DMRs in the vicinity of genes previously implicated in autism supports the notion that disease-relevant gene network and pathways can be identified from peripheral samples. Nonetheless, it would be informative for future studies to assess whether disease-associated epimutations reported from this study are also present in brain samples from ASD patients. Third, informations pertaining to the amniotic and chorionic status of our twin samples are unavailable, preventing us from further dissecting the epigenetic similarity/dissimilarity between twins sharing their placenta and/or amniotic sac. Fourth, the genome-wide platform used for this study (the Illumina 27K array), although robust and highly reliable,^28^ has a somewhat limited density of probe coverage, assaying only one or two CpG sites per gene. Future studies should take advantage of recent advances in genomic profiling technology and perform a more in-depth examination of methylomic differences associated with ASD. Finally, it is difficult to draw conclusions about causality for any of the ASD-associated DMRs identified in this study, in part, because we do not have corresponding RNA expression data, or DNA samples from the twins taken before they became discordant for ASD. It is thus plausible that many of the identified changes have occurred downstream of ASD, for example, resulting from exposure to medications commonly used to treat autistic symptoms. In fact, there is mounting evidence that many drugs used to treat neuropsychiatric disorders induce epigenetic changes.^40^ Such medication-induced changes could still be interesting; an understanding of the pathways via which these drugs work may provide information about the neurobiological processes involved in disease. The ideal study design, however, would assess DNA methylation changes in the brain longitudinally during individuals’ transition into ASD, although such a study does not appear feasible at present.

In summary, this is the first large-scale study to examine the role of genome-wide DNA methylation in ASD and ASD-related traits. Our findings show that: (1) there are numerous DNA methylation differences between MZ twins discordant for ASD and ASD-related traits, as well as between autistic individuals and control samples; (2) many of these DMRs are located in the vicinity of both novel genes and loci that have been previously implicated in ASD; (3) the nature of ASD-associated epimutations is complex with high heterogeneity between individuals; (4) there is high epigenetic heterogeneity between the triad of impairments that define ASD; and (5) there is a quantitative relationship between the severity of the autistic phenotype and DNA methylation at specific CpG sites across the genome. Overall, our findings from this study provide further support for the potential role of DNA methylation in ASD and ASD-related traits.

## Figures and Tables

**Table 1:** The top 50 differentially methylated CpG sites identified in ASD-discordant MZ twin pairs, ranked by statistical significance and mean Δ*β* (calculated as DNA methylation level of ASD twin minus well twin)

*Rank*	*ProbeID*	*Gene*	*Chr.*	*Position*	*Mean* Δβ	P*-value*
1	cg13735974	*NFYC*	1	4092 9567	0.08	3.63E−04
2	cg27321538	*DNPEP*	2	2 1996 0475	0.05	1.33E**−**03
3	cg01447498	*TSNAX*	1	22 9731 294	0.05	9.68E**−**04
4	cg08142684	*TCP1*	6	160 129 858	−0.06	4.29E**−**03
5	cg11241627	*FERD3L*	7	19 151 690	−0.07	4.74E**−**03
6	cg20372689	*RCN2*	15	75 010 774	0.05	2.08E**−**03
7	cg21195120	*MBTPS2*	X	21 768 225	−0.05	3.47E**−**03
8	cg04689061	*PKIA*	8	79 590 548	0.08	6.55E**−**03
9	cg21614638	*DAPP1*	4	100 956 844	0.05	3.48E**−**03
10	cg02639007	*CCDC41*	12	93 377 476	0.05	4.90E**−**03
11	cg15700739	*HOXC5*	12	52 713 967	0.04	8.79E**−**04
12	cg12148581	*RPL14*	3	40 473 347	0.05	6.67E**−**03
13	cg25118574	*PSMB7*	9	126 217 467	0.05	5.09E**−**03
14	cg06284322	*TAF7*	5	140 680 846	−0.05	7.30E**−**03
15	cg23397015	*INHBB*	2	120 818 902	−0.05	8.95E**−**03
16	cg12241297	*HNRPA0*	5	137 118 306	0.05	9.57E**−**03
17	cg13588354	*MC3R*	20	54 256 710	0.04	4.27E**−**03
18	cg10528989	*BDKRB1*	14	95 792 059	0.04	5.48E**−**03
19	cg15836394	*FDFT1*	8	11 697 241	0.04	1.61E**−**03
20	cg21935083	*RAD50*	5	131 920 213	0.05	1.16E**−**02
21	cg03660451	*RECQL5*	17	71 176 201	−0.04	2.80E**−**03
22	cg16166399	*ZNF499*	19	63 722 565	0.06	1.30E**−**02
23	cg23627134	*ARHGAP15*	2	143 602 845	0.05	1.16E**−**02
24	cg05751148	*PTPRCAP*	11	66 961 375	0.04	3.48E**−**03
25	cg18284523	*TINF2*	14	23 781 402	0.04	8.23E**−**03
26	cg20346096	*C18orf22*	18	75 895 818	−0.04	1.96E**−**04
27	cg07200280	*RAFTLIN*	3	16 529 623	−0.04	3.34E**−**03
28	cg12624641	*C14orf143*	14	89 490 322	0.04	8.96E**−**03
29	cg01253545	*RNF185*	22	29 886 314	−0.06	1.48E**−**02
30	cg17367215	*SUPT5H*	19	44 628 460	−0.04	3.15E**−**03
31	cg01894895	*ANXA1*	9	74 956 114	−0.05	1.32E**−**02
32	cg24687764	*C16orf46*	16	79 668 575	−0.04	1.12E**−**02
33	cg20917484	*GAPDH*	12	6 514 327	−0.04	1.74E**−**03
34	cg18776056	*FKBP4*	12	2 775 173	−0.04	1.40E**−**03
35	cg19674669	*LOC112937*	11	133 652 120	0.04	2.91E**−**03
36	cg03875195	*SLC30A3*	2	27 339 867	−0.04	1.09E**−**02
37	cg05520656	*ZNF681*	19	23 733 431	0.04	8.51E**−**03
38	cg18515587	*SELENBP1*	1	149 611 430	0.04	6.46E**−**03
39	cg07239938	*ELA2*	19	803 813	0.04	9.48E**−**03
40	cg01148741	*DUSP2*	2	96 175 180	−0.05	1.40E**−**02
41	cg17002259	*CDC42SE1*	1	149 298 884	0.04	8.37E**−**03
42	cg02154186	*PNMA2*	8	26 427 270	−0.04	7.53E**−**03
43	cg18482268	*POU4F3*	5	145 699 158	−0.04	4.62E**−**03
44	cg20969846	*DIDO1*	20	61 040 666	0.04	4.34E**−**03
45	cg00347904	*SCUBE3*	6	35 290 486	−0.05	1.78E**−**02
46	cg26799474	*CASP8*	2	201 807 196	0.04	5.41E**−**03
47	cg07584959	*THRAP5*	19	843937	−0.04	1.44E**−**02
48	cg19235307	*MBD4*	3	130 642 844	−0.04	1.26E**−**02
49	cg11861730	*ETS1*	11	127 897 893	0.04	8.97E**−**03
50	cg21410991	*ISL1*	5	50 714 208	0.03	1.52E**−**03

Abbreviations: ASD, autism spectrum disorder; MZ, monozygotic.

**Table 2:** Differentially methylated loci highlighted from multiple analytic groups and their relevance to ASD phenotype

*Associated phenotype*	*Reported in analyses*	*Gene*
ASD	(1) Case *versus* control (2) ASD-discordant twins	*NFYC*, *PTPRCAP*
		
	(1) Sporadic *versus* familial ASD (2) ASD-discordant twins	*MBD4*, *RNF185*, *TINF2*
		
Social autistic traits	(1) Discordant twins for social autistic traits (2) Combined autism group	*AFF2*, *GNB2*, *GRB2*, *MAP4*, *PDHX*, *PIK3C3*, *SMEK2*, *THEX1*
		
Autistic RRBIs	(1) Discordant twins for RRBIs (2) Combined discordant group (3) ASD-discordant twins	*TCP1*
	(1) Discordant twins for RRBIs (2) Combined discordant group	*ANKS1A*, *APXL*, *BPI*, *EFTUD2*, *NUDCD3*, *SNRPN*, *SOCS2*
		
Communication autistic traits	(1) Discordant twins for communication autistic traits (2) Case *versus* control	*NUP43*
	(1) Discordant twins for communication autistic traits (2) Combined discordant group	*CCT6A*, *CEP55*, *FCJ12505*, *SRF*

Abbreviations: ASD, autism spectrum disorder; RRBI, restricted repetitive behaviours and interests.
