# Supplementary material for: Methylomic analysis of monozygotic twins discordant for autism spectrum disorder and related behavioural traits
Source: Mol Psychiatry. 2013 Apr 23;19(4):495–503. doi: 10.1038/mp.2013.41 (PMC3906213; doi:10.1038/mp.2013.41)
Supplement: Supplementary Table 1 [file mp201341x1.doc]

| **Group** | **Phenotype Characteristics** | **Number of MZ twin pairs*** | **Number of Individuals*** | **Male:Female Ratio** |
| --- | --- | --- | --- | --- |
| 1 | Discordant for ASD | 6 | 12 | 6M:6F |
| 2 | Discordant for social autistic traits | 9 | 18 | 6M:12F |
| 3 | Discordant for autistic RRBIs | 10 | 20 | 14M:6F |
| 4 | Discordant for communicative autistic traits | 9 | 18 | 12M:6F |
| 5 | Concordant for ASD | 5 | 10 | 10M |
| 6 | Concordant for a low CAST score | 11 | 22 | 8M:14F |
|  | Total | 50 | 100 |  |
