# Supplementary material for: Methylomic analysis of monozygotic twins discordant for autism spectrum disorder and related behavioural traits
Source: Mol Psychiatry. 2013 Apr 23;19(4):495–503. doi: 10.1038/mp.2013.41 (PMC3906213; doi:10.1038/mp.2013.41)
Supplement: Supplementary Table 2 [file mp201341x2.doc]

| **Platform** | **Gene name (Probe ID)** | **Location*** | **Size (bp)** | **Forward primer sequence 5'-3' **** | **Reverse primer sequence 5'-3'** | **Sequencing primer sequence 5'-3'** | **Tm (°C)** | **CpG site** |
| --- | --- | --- | --- | --- | --- | --- | --- | --- |
| Pyrosequencing | *MGC320* (cg16474696) | Chr19:13735953-13736092 | 140 | GGGGATATATTTTTAATTTTAGATAGTAGG | CCACTCCACCACTCCTACCAA | ATTTTTAATTTTAGATAGTAGGTTG | 56 | 4 |
| Pyrosequencing | *OR2L13* (cg20507276) | chr1:246,167,172-246,167,285 | 114 | TTTGAGGGTTTAGGAGGGTT | CTACCCTTCCCCAAAAAACACCAATATATA | TTGAGGGTTTAGGAGGGT | 56 | 7 |
| Sanger Sequencing | *MGC3207* | [chr19:13735941-13736095](http://genome.ucsc.edu/cgi-bin/hgTracks?hgsid=289785921&db=hg18&position=chr19:13735941-13736095&hgPcrResult=pack) | 155 | TACCTTGCCACTGGGGATAC | AACCCACTCCACGACTCCT | TACCTTGCCACTGGGGATAC | 56 | NA |
