# Supplementary material for: Methylomic analysis of monozygotic twins discordant for autism spectrum disorder and related behavioural traits
Source: Mol Psychiatry. 2013 Apr 23;19(4):495–503. doi: 10.1038/mp.2013.41 (PMC3906213; doi:10.1038/mp.2013.41)
Supplement: Supplementary Table 3 [file mp201341x3.pdf]

Discordant ASD MZ Twin-pair	Gene	Probe	Δβ≤ -0.15	Gene	Probe	Δβ≥ 0.15
1	*FLJ42486*	cg03734874	-0.17	*HOXA9*	cg27009703	0.19
	GLRA3	cg21094669	-0.16	*HHEX*	cg09721427	0.16
	PDE5A	cg07154408	-0.16			
	SRF	cg16041611	-0.16			
	IRX2	cg15433631	-0.15			
	NPTX2	cg12799895	-0.15			
2	*CTCF*	cg01866162	-0.36	*SYNJ2*	cg13645811	0.19
	PXDN	cg12164282	-0.19	*STYX*	cg03803589	0.17
	RPL7	cg03991326	-0.17	*HNRPD*	cg07964133	0.16
	CCL23	cg14916288	-0.16	*TM6SF1*	cg14696396	0.16
				SMS	cg18624866	0.15
3	*C11orf1*	cg04545708	-0.35	*ZDHHC24*	cg08420085	0.31
	TACR3	cg04263186	-0.17	*KCNJ5*	cg11387131	0.19
				HCRTR2	cg13948987	0.19
				TCP11	cg22407458	0.18
				CRMP1	cg03544320	0.17
				PHKA2	cg07314984	0.17
				ATP5J	cg15707568	0.16
				SLC30A3	cg19461344	0.16
4	*NDUFB2*	cg12318316	-0.29	*MGC9850*	cg21097640	0.43
	PXDN	cg12164282	-0.28	*SLC38A1*	cg16469386	0.35
	ABCC4	cg15868151	-0.27	*DGKQ*	cg17592292	0.30
	RGS7	cg21303386	-0.24	*CYP46A1*	cg01117627	0.28
	FLJ20422	cg20426860	-0.21	*CTNNB1*	cg04180460	0.27
	DLX5	cg02101486	-0.20	*JOSD1*	cg26380756	0.26
	AGMAT	cg11706911	-0.19	*FGFR1OP*	cg25395108	0.25
	HOXB7	cg06493080	-0.19	*UBE2Q1*	cg09311052	0.24
	HECA	cg24349555	-0.19	*PSMD2*	cg19188060	0.24
	RTN4RL2	cg17349753	-0.18	*CD24*	cg23654549	0.24
	NMNAT3	cg01724150	-0.17	*SACM1L*	cg22986271	0.23
	C6orf89	cg20598389	-0.17	*KCNQ1*	cg17575811	0.23
	SLC39A8	cg18277508	-0.16	*CRLF1*	cg10810921	0.23
	ZNF502	cg08210616	-0.16	*KCNQ3*	cg27016990	0.22
	IMPA1	cg16746631	-0.16	*GABBR1*	cg24847163	0.22
	BOP1	cg11375622	-0.16	*HOXA9*	cg27009703	0.21
	ZC3H10	cg05347567	-0.16	*SMG1*	cg07295034	0.21
	C19orf6	cg23792364	-0.16	*CPZ*	cg03292388	0.21
	ACCN2	cg23126949	-0.16	*RASGRF2*	cg09952204	0.21
	STARD13	cg26049501	-0.16	*STOML1*	cg00441382	0.20
				HS3ST3A1	cg07480567	0.20
				BAHD1	cg07114024	0.19
				POLS	cg20149766	0.19
				SLC30A2	cg05674944	0.19
				ALDOB	cg26181372	0.18
				HR	cg12748258	0.18
				FLJ30934	cg19018097	0.18
				WT1	cg15107670	0.18
				HIAT1	cg02161046	0.18
				ITPK1	cg16628119	0.18
				VAMP5	cg11108890	0.17

				C21orf7	cg22074666	0.17
				DNMT3B	cg16523653	0.17
				GRB10	cg06790324	0.17
				MARCH7	cg02479196	0.17
				XPR1	cg16394138	0.16
				ATP5S	cg15383574	0.16
				ATP13A4	cg23159337	0.16
				AMDHD2	cg09548179	0.16
				RARB	cg02499249	0.16
				CHEK2	cg16189954	0.16
				PCTK3	cg07713493	0.16
				NUP43	cg26675382	0.16
				HOXA9	cg26521404	0.16
				LOC51315	cg26602920	0.15
				SLC6A15	cg03064067	0.15
				MGC26816	cg08431931	0.15
				FCRL1	cg09340639	0.15
				LOXL1	cg03682712	0.15
5	*MATN3*	cg21177096	-0.17	*FLJ25422*	cg24237439	0.18
	NKX6-2	cg08441806	-0.17	*MRPL12*	cg15214092	0.17
	ERCC2	cg04878842	-0.17	*ITGB7*	cg08374799	0.16
	CSMD2	cg19228118	-0.16	*SUSD1*	cg14862827	0.15
	KIF2C	cg03907454	-0.16	*REPS2*	cg20855303	0.15
	SURF4	cg22244122	-0.16	*INSIG2*	cg04195527	0.15
6	*CYSLTR2*	cg18236297	-0.34	*FLJ20422*	cg20426860	0.27
	PTRH2	cg08793459	-0.25	*EIF3S6*	cg06987504	0.24
	ADPN	cg23653187	-0.25	*TTC1*	cg01222684	0.24
	ABCC12	cg14074641	-0.24	*GRIA4*	cg09980522	0.23
	C11orf1	cg04545708	-0.23	*C20orf38*	cg25125453	0.22
	LRP2BP	cg06521761	-0.22	*IGF2BP3*	cg02860543	0.22
	CD209	cg07608333	-0.21	*ACAT2*	cg15298323	0.21
	HS6ST3	cg17183991	-0.21	*TARDBP*	cg00692549	0.21
	TTLL1	cg09047884	-0.20	*TRIM17*	cg23540518	0.21
	MMP10	cg00347729	-0.20	*EFHA1*	cg08163803	0.20
	MGC16186	cg12064929	-0.20	*CCNT2*	cg17865752	0.20
	ABCB11	cg20118424	-0.19	*ATP2C1*	cg09134747	0.20
	REPIN1	cg21784940	-0.19	*C6orf105*	cg14178895	0.19
	YES1	cg14671488	-0.19	*C10orf63*	cg08847038	0.19
	C4orf6	cg11237738	-0.18	*BCDIN3*	cg17607973	0.19
	FSBP	cg26323655	-0.18	*COBLL1*	cg02537909	0.18
	CYP4F2	cg05358291	-0.18	*SP100*	cg05091653	0.18
	CIRH1A	cg17120423	-0.17	*TCN1*	cg00187686	0.18
	GUCY1A3	cg02210887	-0.17	*SPATA12*	cg04563996	0.18
	SCEL	cg21063899	-0.17	*PPBPL2*	cg26866325	0.18
	GIMAP4	cg00323915	-0.17	*MET*	cg25299676	0.18
	ALS2CR7	cg19654437	-0.17	*EI24*	cg26606064	0.17
	ALDH3B2	cg14353201	-0.17	*HIST1H4E*	cg16706631	0.17
	PPAP2B	cg16505550	-0.17	*DYSF*	cg15491567	0.17
	C14orf119	cg16141690	-0.17	*SFRS2*	cg00953277	0.17
	FLJ32926	cg11521325	-0.16	*PAM*	cg21596317	0.17
	IGSF2	cg08920071	-0.16	*PIGB*	cg27638672	0.17
	FLJ20160	cg15998761	-0.16	*KIAA0372*	cg09244244	0.17
	KIAA1267	cg19832721	-0.16	*DMTF1*	cg19861697	0.17

	CXCL11	cg08046471	-0.16	*EVC*	cg21210758	0.17
	AGA	cg00398048	-0.16	*ASPM*	cg19350340	0.17
	CTLA4	cg08460026	-0.16	*FMO4*	cg21183846	0.17
	JPH1	cg23599843	-0.15	*ZNF235*	cg27541515	0.16
	CDO1	cg07644368	-0.15	*SNRPD2*	cg24254206	0.16
	SULT1C2	cg17966192	-0.15	*ATP5J*	cg25085950	0.16
	CD3E	cg24612198	-0.15	*CDKN2A*	cg26673943	0.16
	ITGA3	cg14737977	-0.15	*FGF8*	cg16584573	0.16
	LRRC50	cg08965527	-0.15	*ITLN1*	cg21476940	0.16
				TEP1	cg16920242	0.16
				HIST2H2AC	cg03086563	0.16
				PLEKHO1	cg00095674	0.16
				GFPT1	cg08646988	0.16
				C1orf9	cg20525378	0.16
				TGFB2	cg11976166	0.16
				NR1H2	cg00336605	0.16
				TMEM128	cg13296371	0.16
				OR10H2	cg12513379	0.16
				ILF2	cg09929564	0.16
				HIST1H1E	cg15519208	0.15
				MOSPD2	cg19740287	0.15
				MGC41945	cg05000446	0.15
				VPS18	cg03643709	0.15
				CXorf42	cg24038764	0.15
