# Supplementary material for: Methylomic analysis of monozygotic twins discordant for autism spectrum disorder and related behavioural traits
Source: Mol Psychiatry. 2013 Apr 23;19(4):495–503. doi: 10.1038/mp.2013.41 (PMC3906213; doi:10.1038/mp.2013.41)
Supplement: Supplementary Table 4 [file mp201341x4.pdf]

a

Rank	ProbeID	Gene	Chromosome	Position	Mean $\Delta\beta$	p-value
1	cg15089487	*THAP10*	15	68972306	0.04	1.30E-03
2	cg11324740	*OIP5*	15	39412085	0.03	6.05E-04
3	cg15240064	*ZNF12*	7	6713362	0.04	2.04E-03
4	cg01353347	*IRAK1*	X	152938352	-0.03	6.67E-04
5	cg22226839	*ATP2B4*	1	201862674	-0.04	5.25E-03
6	cg21241823	*PRDM15*	21	42173093	-0.04	4.54E-03
7	cg15940569	*GABRB3*	15	24570449	0.04	4.25E-03
8	cg11909310	*DKFZP686A10121*	7	89813722	0.06	7.30E-03
9	cg12736438	*RAB20*	13	110012458	-0.03	1.74E-03
10	cg01041367	*TMEM70*	8	75050634	0.04	5.91E-03
11	cg04244987	*NCKIPSD*	3	48698642	-0.03	4.10E-03
12	cg18349258	*SMEK2*	2	55697707	0.04	6.00E-03
13	cg11492856	*CAPN7*	3	15221591	-0.03	3.55E-03
14	cg15995002	*RAD54B*	8	95556400	0.03	3.34E-03
15	cg01798443	*RPSA*	3	39422895	0.03	4.43E-03
16	cg24034289	*SCAMP3*	1	153498968	-0.03	3.62E-03
17	cg25948180	*PDHX*	11	34893357	0.04	8.09E-03
18	cg19837131	*PIK3C3*	18	37789059	-0.05	1.01E-02
19	cg11814446	*LEMD3*	12	63849263	0.03	5.79E-03
20	cg27537561	*VDR*	12	46584923	-0.04	8.87E-03
21	cg01160766	*RPL38*	17	69711466	-0.03	7.81E-03
22	cg18855178	*PCSK4*	19	1441194	0.03	4.51E-03
23	cg08785133	*PORCN*	X	48252137	-0.03	4.24E-03
24	cg13240311	*PKIG*	20	42594209	-0.03	8.51E-03
25	cg20663980	*CDX4*	X	72583795	0.03	9.03E-04
26	cg27198824	*AFF2*	X	147390419	-0.03	9.52E-03
27	cg05857825	*MAPRE1*	20	30871874	-0.03	6.28E-03
28	cg21509097	*LYPLA3*	16	66837114	-0.03	7.45E-03
29	cg02345317	*NLGN3*	X	70281268	-0.03	1.27E-04
30	cg16468910	*SNX4*	3	126721481	0.03	1.10E-02
31	cg14751914	*SMAD7*	18	44731402	-0.04	1.46E-02
32	cg25095380	*PRDM5*	4	122062657	0.03	1.29E-02
33	cg04856685	*MAP4*	3	48105803	-0.03	5.69E-03
34	cg19695867	*WDR47*	1	109386913	0.03	9.43E-03
35	cg13192155	*ERAS*	X	48570887	-0.03	9.45E-03
36	cg11223864	*GNB2*	7	100109259	-0.03	1.16E-02
37	cg17150465	*SCRIB*	8	144969546	-0.03	1.18E-03
38	cg05471775	*HIST1H4B*	6	26135510	-0.03	9.47E-03
39	cg04959788	*PTCH2*	1	45081216	-0.03	5.76E-03
40	cg17758148	*JMY*	5	78609397	0.03	1.86E-03
41	cg14272175	*PXK*	3	58294000	0.03	7.48E-03
42	cg26757053	*LYPLAL1*	1	217413644	0.04	1.75E-02
43	cg00620629	*C6orf113*	6	117096644	0.03	1.27E-02
44	cg07118638	*THEX1*	8	8897611	0.03	9.99E-03
45	cg19724470	*CD274*	9	5440936	0.03	1.71E-02
46	cg05158538	*PPARGC1A*	4	23500584	0.03	1.17E-02
47	cg26365553	*MADD*	11	47247189	-0.02	7.85E-04
48	cg13585240	*ARIH2*	3	48934379	0.02	1.65E-03
49	cg04156850	*GRB2*	17	70914167	-0.03	7.12E-03
50	cg24130043	*ZNF197*	3	44641366	-0.02	3.52E-03

b

Rank	ProbeID	Gene	Chromosome	Position	Mean $\Delta\beta$	p-value
1	cg16746631	*IMPA1*	8	82760695	-0.04	4.72E-04
2	cg04624659	*SPAG17*	1	118529340	-0.04	7.51E-04
3	cg27372468	*SLC22A4*	5	131658111	0.04	1.54E-04
4	cg16208448	*TPP2*	13	102047992	-0.06	2.79E-03
5	cg18215716	*TMEM111*	3	10004246	0.04	2.00E-03
6	cg05107152	*KLC4*	6	43134752	0.04	2.66E-03
7	cg04062907	*ANAPC7*	12	109325928	0.03	7.55E-06
8	cg12930602	*DIPA*	11	65414471	0.04	3.01E-03
9	cg24110050	*TCTEX1D1*	1	66990441	-0.04	3.19E-03
10	cg13700897	*RSPO2*	8	109165240	0.03	1.49E-03
11	cg02210123	*RHOJ*	14	62740588	-0.04	3.46E-03
12	cg02597128	*CXorf41*	X	106336479	-0.05	5.71E-03
13	cg16082125	*USP11*	X	46977504	-0.04	3.32E-03
14	cg08463061	*RND3*	2	151052578	-0.06	6.38E-03
15	cg16541031	*IRF7*	11	605519	-0.03	2.36E-04
16	cg11738543	*SOCS2*	12	92491356	-0.03	2.99E-03
17	cg09665351	*APXL*	X	9715283	0.04	5.83E-03
18	cg16290693	*SPSB1*	1	9275539	-0.05	6.51E-03
19	cg10159529	*IL5RA*	3	3127530	0.03	1.87E-03
20	cg25909811	*KDELR2*	7	6489778	0.03	1.18E-03
21	cg01808130	*SLC35B3*	6	8380205	-0.04	7.02E-03
22	cg14155482	*PIP5K1A*	1	149437706	0.03	5.21E-03
23	cg22492966	*JMJD1C*	10	64698935	0.04	7.92E-03
24	cg08142684	*TCP1*	6	160129858	-0.04	6.12E-03
25	cg10274830	*C6orf96*	6	151815702	-0.05	9.03E-03
26	cg27198824	*AFF2*	X	147390419	-0.03	4.78E-03
27	cg05459203	*NUDCD3*	7	44497199	-0.04	9.22E-03
28	cg27631817	*OFCC1*	6	10168924	-0.04	8.12E-03
29	cg23828595	*PRKG1*	10	52503616	-0.03	5.73E-03
30	cg18506672	*SNURF*	15	22751346	-0.03	2.74E-03
31	cg07623294	*ELAVL2*	9	23816507	-0.03	5.71E-03
32	cg13181019	*MPP7*	10	28611588	0.03	4.99E-04
33	cg06618866	*TLR2*	4	154824537	-0.03	6.71E-03
34	cg23653712	*SGCB*	4	52599136	-0.03	7.65E-04
35	cg17746675	*C3orf31*	3	11863297	0.03	7.97E-03
36	cg02200584	*PDGFC*	4	158111996	-0.03	3.43E-03
37	cg12542604	*ANKS1A*	6	34964682	0.04	1.09E-02
38	cg02171545	*SNRPN*	15	22644459	-0.04	1.32E-02
39	cg14967972	*PANK1*	10	91395245	-0.04	1.38E-02
40	cg17412258	*DLK1*	14	100262770	-0.03	3.27E-03
41	cg02712845	*DPAGT1*	11	118478248	-0.05	1.43E-02
42	cg20902737	*EFTUD2*	17	40331968	0.03	3.53E-03
43	cg11692477	*SLC40A1*	2	190153722	-0.03	3.62E-03
44	cg00468146	*ID4*	6	19946190	-0.03	8.41E-03
45	cg09053680	*UTF1*	10	134894104	-0.03	3.14E-03
46	cg06905514	*CAMK2B*	7	44331466	-0.03	7.05E-03
47	cg24921089	*AMPD3*	11	10429416	-0.03	1.02E-02
48	cg03863149	*ZNF644*	1	91260104	-0.04	1.36E-02
49	cg15494458	*BPI*	20	36364926	0.03	9.51E-03
50	cg26491425	*RHOV*	15	38953924	0.03	6.50E-03

C

Rank	ProbeID	Gene	Chromosome	Position	Mean Δβ	p-value
1	cg16399745	*CNAP1*	12	6474300	-0.06	1.17E-03
2	cg06340713	*CHM*	X	85190083	0.05	5.03E-04
3	cg12237946	*PGBD4*	15	32181668	-0.07	2.73E-03
4	cg02245418	*ZNF364*	1	144321032	0.04	9.45E-04
5	cg25040783	*APPL*	3	57236346	0.04	1.53E-03
6	cg05881762	*UBE3A*	15	23235942	0.04	3.38E-03
7	cg04578090	*PROCA1*	17	24063650	-0.05	5.86E-03
8	cg22947000	*BCMO1*	16	79829782	0.06	6.99E-03
9	cg24371383	*CEP55*	10	95246094	0.04	2.90E-03
10	cg22232206	*SRF*	6	43246163	-0.04	1.75E-03
11	cg24073051	*CDV3*	3	134774669	0.03	2.07E-03
12	cg01485998	*FLJ12505*	1	211190520	-0.04	4.66E-03
13	cg00662775	*TCEAL4*	X	102727091	-0.07	8.90E-03
14	cg23054437	*MOSC2*	1	218987929	-0.05	7.67E-03
15	cg00901652	*SH2BP1*	11	10729010	-0.04	7.86E-03
16	cg23839680	*CCT6A*	7	56085732	-0.06	9.63E-03
17	cg09580336	*ATP1A1*	1	116716645	0.04	5.86E-03
18	cg14196790	*SLC22A5*	5	131732934	-0.04	5.69E-03
19	cg04902405	*ZC3H11A*	1	202035140	0.04	9.01E-03
20	cg11392765	*BAPX1*	4	13155771	-0.04	6.32E-03
21	cg05419984	*PIGX*	3	197922860	-0.04	9.89E-03
22	cg02620013	*MLNR*	13	48692682	-0.03	4.58E-03
23	cg20649047	*NUMB*	14	72994914	-0.04	7.70E-03
24	cg00325491	*FN5*	11	92925818	0.03	2.76E-03
25	cg26536259	*BMP2*	20	6696006	-0.03	3.71E-03
26	cg04145477	*QRSL1*	6	107184322	-0.03	1.30E-03
27	cg13306784	*INPP5E*	9	138454309	-0.04	1.05E-02
28	cg10521267	*ZNF684*	1	40769714	0.04	1.15E-02
29	cg01909833	*BRD2*	6	33045245	-0.03	6.18E-03
30	cg27170298	*BCS1L*	2	219233031	-0.04	1.30E-02
31	cg12073779	*CRYGD*	2	208697714	-0.03	9.22E-03
32	cg05944800	*INSIG2*	2	118562016	-0.03	7.09E-03
33	cg26083396	*IMPDH1*	7	127837121	-0.04	1.47E-02
34	cg05062178	*ITGB5*	3	126087937	0.03	4.67E-03
35	cg11395610	*RAB28*	4	13094336	-0.03	8.61E-03
36	cg04422896	*C12orf43*	12	119938652	-0.03	8.63E-03
37	cg26675382	*NUP43*	6	150109539	0.05	1.80E-02
38	cg05768141	*KCNJ10*	1	158306471	0.03	1.22E-02
39	cg12179176	*SNX19*	11	130291765	0.03	1.09E-02
40	cg25921910	*CDC73*	1	191357280	0.04	1.65E-02
41	cg17572791	*HAX1*	1	152511809	-0.03	1.29E-02
42	cg04587910	*XLF*	2	219733988	0.03	4.37E-03
43	cg19848683	*EVI1*	3	170346742	-0.03	4.47E-03
44	cg24456340	*GNGT2*	17	44641482	-0.03	3.14E-03
45	cg08513100	*UBE2B*	5	133734621	-0.03	1.37E-02
46	cg15182360	*PCDH20*	13	60887738	-0.04	1.92E-02
47	cg20716209	*STAT3*	17	37794796	-0.03	1.23E-02
48	cg12917695	*HSPC268*	7	138675699	-0.03	8.90E-03
49	cg23062876	*DBT*	1	100488055	-0.03	1.21E-02
50	cg14800883	*ARPC4*	3	9809004	0.05	2.18E-02
