# Supplementary material for: Methylomic analysis of monozygotic twins discordant for autism spectrum disorder and related behavioural traits
Source: Mol Psychiatry. 2013 Apr 23;19(4):495–503. doi: 10.1038/mp.2013.41 (PMC3906213; doi:10.1038/mp.2013.41)
Supplement: Supplementary Table 5 [file mp201341x5.pdf]

Rank	ProbeID	Gene	Chr	Position	Mean Δβ (Minimum-Maximum)	p-value
1	cg19837131	*PIK3C3*	18	37789059	-0.04 (-0.12 - 0.06)	3.68E-05
2	cg18349258	*SMEK2*	2	55697707	0.02 (-0.04 - 0.1)	1.84E-04
3	cg12478185	*SCO1*	17	10542726	-0.03 (-0.17 - 0.09)	9.01E-04
4	cg27198824	*AFF2*	X	147390419	-0.02 (-0.09 - 0.05)	3.87E-04
5	cg11223864	*GNB2*	7	100109259	-0.02 (-0.09 - 0.03)	6.20E-04
6	cg21926138	*C1orf88*	1	111690943	-0.02 (-0.1 - 0.03)	6.70E-04
7	cg25095951	*RPS26*	12	54721514	0.02 (-0.06 - 0.09)	1.57E-03
8	cg04961553	*OCIAD2*	4	48603226	-0.02 (-0.05 - 0.04)	2.03E-05
9	cg19318511	*C9orf30*	9	102228979	-0.02 (-0.1 - 0.04)	1.96E-03
10	cg23412850	*SOCS2*	12	92491518	-0.02 (-0.09 - 0.04)	1.86E-03
11	cg12542604	*ANKS1A*	6	34964682	0.02 (-0.06 - 0.13)	4.15E-03
12	cg08142684	*TCP1*	6	160129858	-0.02 (-0.11 - 0.07)	3.92E-03
13	cg23839680	*CCT6A*	7	56085732	-0.02 (-0.14 - 0.08)	4.96E-03
14	cg25948180	*PDHX*	11	34893357	0.02 (-0.05 - 0.13)	4.56E-03
15	cg01485998	*FLJ12505*	1	211190520	-0.02 (-0.07 - 0.03)	5.56E-04
16	cg08729012	*CMTM6*	3	32518632	0.02 (-0.04 - 0.08)	1.40E-03
17	cg24371383	*CEP55*	10	95246094	0.02 (-0.06 - 0.07)	5.69E-04
18	cg07118638	*THEX1*	8	8897611	0.02 (-0.02 - 0.06)	1.64E-04
19	cg24364574	*PREX1*	20	46877101	0.02 (-0.05 - 0.13)	5.11E-03
20	cg12623088	*MGC4562*	15	64372575	-0.02 (-0.08 - 0.04)	1.97E-03
21	cg05815906	*RAB33B*	4	140593788	0.02 (-0.05 - 0.09)	2.72E-03
22	cg04856685	*MAP4*	3	48105803	-0.02 (-0.1 - 0.05)	1.41E-03
23	cg25890048	*OR5I1*	11	55460019	-0.02 (-0.09 - 0.06)	2.22E-03
24	cg14663065	*CTNNB1*	3	41216634	0.02 (-0.05 - 0.07)	1.64E-03
25	cg11164400	*PPP1R9A*	7	94374723	-0.02 (-0.07 - 0.03)	2.49E-03
26	cg05072951	*CAPN1*	11	64705974	0.02 (-0.04 - 0.08)	2.09E-03
27	cg11810837	*ARMCX5*	X	101740932	-0.02 (-0.12 - 0.04)	6.11E-03
28	cg18710985	*C20orf23*	20	16502249	-0.02 (-0.07 - 0.05)	3.89E-03
29	cg05486551	*FLJ25067*	20	5679259	0.03 (-0.09 - 0.12)	9.18E-03
30	cg20902737	*EFTUD2*	17	40331968	0.02 (-0.04 - 0.07)	2.58E-03
31	cg21006686	*NOS1*	12	116284623	0.02 (-0.04 - 0.11)	5.71E-03
32	cg17398595	*SH3GL2*	9	17568725	-0.02 (-0.12 - 0.05)	4.21E-03
33	cg09665351	*APXL*	X	9715283	0.02 (-0.08 - 0.1)	1.02E-02
34	cg21829265	*ZNF451*	6	57019098	-0.02 (-0.08 - 0.04)	2.22E-03
35	cg04156850	*GRB2*	17	70914167	-0.02 (-0.07 - 0.02)	8.43E-04
36	cg02441647	*COL8A1*	3	100840189	0.02 (-0.04 - 0.09)	4.58E-03
37	cg19430430	*COL5A3*	19	9982323	0.02 (-0.02 - 0.07)	4.87E-04
38	cg00250430	*DMRT2*	9	1041579	-0.02 (-0.07 - 0.03)	1.51E-03
39	cg15683488	*ZFYVE9*	1	52380059	0.02 (-0.06 - 0.09)	6.85E-03
40	cg23146358	*CDKN1C*	11	2862072	0.02 (-0.07 - 0.14)	1.03E-02
41	cg22442730	*SRMS*	20	61649677	-0.02 (-0.08 - 0.07)	5.55E-03
42	cg02337166	*NR1D1*	17	35510829	-0.02 (-0.08 - 0.07)	9.38E-03
43	cg13573276	*RDHE2*	8	57395452	0.01 (-0.01 - 0.07)	5.51E-04
44	cg26251865	*IRGC*	19	48912054	0.02 (-0.05 - 0.07)	3.37E-03
45	cg22232206	*SRF*	6	43246163	-0.02 (-0.14 - 0.05)	9.26E-03
46	cg15494458	*BPI*	20	36364926	0.02 (-0.06 - 0.1)	7.48E-03
47	cg02787991	*SECTM1*	17	77884694	0.02 (-0.04 - 0.08)	3.15E-03
48	cg08228917	*LHFP*	13	39075062	-0.01 (-0.07 - 0.03)	1.15E-03
49	cg05960806	*CLNS1A*	11	77026532	-0.02 (-0.07 - 0.03)	3.93E-03
50	cg08137040	*LOC168850*	7	126820931	-0.02 (-0.11 - 0.05)	1.17E-02
