# Supplementary material for: Methylomic analysis of monozygotic twins discordant for autism spectrum disorder and related behavioural traits
Source: Mol Psychiatry. 2013 Apr 23;19(4):495–503. doi: 10.1038/mp.2013.41 (PMC3906213; doi:10.1038/mp.2013.41)
Supplement: Supplementary Table 6 [file mp201341x6.pdf]

a

Rank	ProbeID	Gene	Chromosome	Position	Mean Δβ	p-value
1	cg16474696	*MGC3207*	19	13736014	-0.24	1.54E-04
2	cg15379633	*RAB36*	22	21817586	0.08	1.59E-05
3	cg17421623	*C3orf9*	3	120670260	0.07	1.06E-04
4	cg23815491	*HP*	16	70646123	0.11	5.08E-04
5	cg14119236	*FGF23*	12	4359336	0.05	6.46E-05
6	cg17408686	*CHCHD6*	3	127905265	0.07	2.79E-04
7	cg26029902	*RAB22A*	20	56317831	-0.07	2.82E-04
8	cg04743872	*FLJ20701*	2	229843821	-0.05	2.89E-05
9	cg14329157	*WDR69*	2	228444379	0.04	8.82E-06
10	cg08909157	*C9orf66*	9	205561	0.05	2.45E-04
11	cg14920334	*GALNT8*	12	4700088	0.05	2.81E-04
12	cg22584138	*SLC6A4*	17	25586346	-0.05	6.06E-04
13	cg21717724	*PSMD5*	9	122644335	0.13	1.63E-03
14	cg22778947	*FSD1NL*	9	107250885	0.05	1.25E-03
15	cg13620770	*BAD*	11	63808314	0.04	3.53E-04
16	cg08598221	*SNTB1*	8	121894110	0.05	1.37E-03
17	cg08137040	*LOC168850*	7	126820931	-0.04	8.39E-04
18	cg26675382	*NUP43*	6	150109539	0.04	1.18E-03
19	cg21226225	*TXNRD1*	12	103204872	0.04	8.47E-04
20	cg19328294	*PRSS22*	16	2848555	0.06	2.13E-03
21	cg06392241	*NUDT4*	12	92295477	0.04	4.68E-04
22	cg08942800	*CRISP2*	6	49789269	-0.04	1.27E-03
23	cg26233209	*ATG12*	5	115206663	0.04	1.07E-03
24	cg08085267	*C17orf57*	17	42756832	-0.06	2.37E-03
25	cg24424381	*PEX11A*	15	88034861	-0.04	2.81E-04
26	cg20540428	*PPP4R2*	3	73128376	-0.09	2.94E-03
27	cg26538349	*FLJ20035*	4	169476425	-0.03	3.90E-04
28	cg20721467	*MYST4*	10	76267933	0.05	2.70E-03
29	cg02671171	*RPH3AL*	17	203087	0.03	1.13E-04
30	cg16970232	*APC*	5	112101332	-0.03	2.32E-04
31	cg12000587	*C17orf79*	17	27210743	0.03	6.33E-04
32	cg21911019	*GPR78*	4	8632550	0.04	1.51E-03
33	cg17945001	*IGSF21*	1	18306705	0.03	1.21E-03
34	cg14056644	*PITX2*	4	111778554	0.04	2.06E-03
35	cg07753644	*P2RY11*	19	10083175	0.04	2.92E-03
36	cg13030582	*MFAP4*	17	19231301	0.03	1.27E-03
37	cg19384697	*UPK3B*	7	75977858	0.03	2.01E-04
38	cg24879335	*TF*	3	134947870	-0.04	2.65E-03
39	cg13206017	*SST*	3	188870919	-0.04	3.72E-03
40	cg20843052	*KIAA1841*	2	61147229	0.03	3.68E-04
41	cg19573166	*SLC22A17*	14	22892248	0.04	2.83E-03
42	cg13652336	*DEPDC2*	8	69026566	0.03	1.41E-03
43	cg09682183	*UNC93A*	6	167625243	0.04	3.56E-03
44	cg18960218	*SLC7A7*	14	22355205	0.04	2.45E-03
45	cg03330058	*ABTB1*	3	128875093	0.04	2.71E-03
46	cg05751148	*PTPRCAP*	11	66961375	0.03	1.39E-03
47	cg13735974	*NFYC*	1	40929567	0.04	3.26E-03
48	cg26922202	*OR2S2*	9	35947984	0.03	2.25E-03
49	cg05535113	*CHST4*	16	70117080	0.04	3.26E-03
50	cg15584813	*SLC38A4*	12	45505893	0.08	6.20E-03

b

Rank	ProbeID	Gene	Chromosome	Position	Mean $\Delta\beta$	p-value
1	cg07665060	*C19orf33*	19	43486438	-0.12	7.40E-04
2	cg17006282	*RPL36*	19	5640943	0.07	2.92E-04
3	cg01353448	*C7orf16*	7	31693437	-0.09	1.62E-03
4	cg09547224	*SLC5A1*	22	30769259	-0.1	2.76E-03
5	cg17571291	*BLVRA*	7	43764312	-0.13	3.91E-03
6	cg01511567	*SSRP1*	11	56860207	-0.06	9.29E-04
7	cg00027083	*EPB41L3*	18	5533801	-0.08	3.21E-03
8	cg16685388	*HIVEP3*	1	42156643	-0.09	5.58E-03
9	cg08020808	*CMA1*	14	24047613	-0.08	4.49E-03
10	cg14847483	*TMEM85*	15	32303932	-0.09	6.31E-03
11	cg25434223	*ELAVL3*	19	11452491	-0.05	1.89E-03
12	cg12437481	*MRPL28*	16	360113	0.13	9.45E-03
13	cg01294695	*MESP1*	15	88095644	0.07	6.67E-03
14	cg11126134	*FLJ14834*	13	30378304	-0.07	6.87E-03
15	cg11096993	*ACY3*	11	67174534	-0.07	7.96E-03
16	cg14972143	*EIF4E*	4	100070026	-0.05	5.30E-03
17	cg17838516	*MTNR1B*	11	92343184	-0.05	5.21E-03
18	cg21667836	*PRMT8*	12	3469616	-0.05	3.39E-03
19	cg18771300	*RHOJ*	14	62741490	-0.28	1.17E-02
20	cg19668234	*TSP50*	3	46734453	0.07	8.95E-03
21	cg06131859	*KYNU*	2	143351601	0.05	4.93E-03
22	cg06148175	*ACY3*	11	67174724	-0.07	9.75E-03
23	cg02630694	*C10orf7*	10	12276839	-0.06	9.86E-03
24	cg08040471	*C17orf62*	17	78001068	-0.04	9.19E-05
25	cg20832020	*VSIG9*	3	115495602	-0.05	7.86E-03
26	cg04384398	*PMM1*	22	40316279	0.05	5.71E-03
27	cg16357381	*COL7A1*	3	48607896	-0.04	1.31E-03
28	cg25250358	*PLOD2*	3	147361774	-0.05	7.75E-03
29	cg18432105	*MYH2*	17	10394235	-0.05	7.30E-03
30	cg18284523	*TINF2*	14	23781402	0.05	5.11E-03
31	cg01253545	*RNF185*	22	29886314	-0.05	6.89E-03
32	cg00498305	*SLC18A2*	10	118990226	-0.06	1.04E-02
33	cg05189291	*ICF45*	5	157091035	-0.05	8.28E-03
34	cg22313024	*UQCRB*	8	97317245	-0.05	8.03E-03
35	cg19235307	*MBD4*	3	130642844	-0.04	5.77E-03
36	cg10214058	*CNOT8*	5	154218362	0.05	6.93E-03
37	cg17749961	*LYCAT*	2	30523367	0.07	1.40E-02
38	cg08440425	*LRRC51*	11	71468383	-0.04	5.43E-03
39	cg19404582	*GRIN1*	9	139152774	-0.04	2.01E-03
40	cg19408398	*FIP1L1*	4	53938085	-0.06	1.49E-02
41	cg20663831	*GIMAP2*	7	150014087	-0.05	1.22E-02
42	cg13234848	*AUTS2*	7	68701907	0.04	4.31E-03
43	cg13311440	*CD48*	1	158948028	-0.04	7.22E-03
44	cg14951292	*HMOX2*	16	4465987	-0.06	1.56E-02
45	cg10562586	*MAP2*	2	210152399	-0.06	1.39E-02
46	cg18236734	*HTR3E*	3	185300625	-0.05	1.13E-02
47	cg03962522	*SLC5A1*	22	30769254	-0.08	1.71E-02
48	cg10784813	*SOCS1*	16	11256179	-0.05	1.04E-02
49	cg18632631	*TNK1*	17	7224773	0.06	1.51E-02
50	cg07758574	*C3orf62*	3	49289374	-0.04	6.79E-03
