# Supplementary material for: Methylomic analysis of monozygotic twins discordant for autism spectrum disorder and related behavioural traits
Source: Mol Psychiatry. 2013 Apr 23;19(4):495–503. doi: 10.1038/mp.2013.41 (PMC3906213; doi:10.1038/mp.2013.41)
Supplement: Supplementary Table 7 [file mp201341x7.pdf]

| ProbeID | Gene | Chr | Position | Rank in analysis group* | | | | | | |
|---|---|---|---|---|---|---|---|---|---|---|
| | | | | **1** | **2** | **3** | **4** | **5** | **6** | **7** |
| **cg13735974** | ***NFYC*** | **1** | **40929567** | **1** | 18192 | 4985 | 17744 | 3700 | **47** | 12151 |
| cg27321538 | *DNPEP* | 2 | 219960475 | **2** | 2256 | 22215 | 2401 | 22559 | 603 | 210 |
| cg01447498 | *TSNAX* | 1 | 229731294 | **3** | 20866 | 12183 | 3450 | 121 | 10362 | 282 |
| **cg08142684** | ***TCP1*** | **6** | **160129858** | **4** | 3663 | **24** | 1050 | **12** | 18711 | 4192 |
| cg11241627 | *FERD3L* | 7 | 19151690 | **5** | 7126 | 14778 | 15316 | 7432 | 11957 | 395 |
| cg20372689 | *RCN2* | 15 | 75010774 | **6** | 23248 | 6594 | 1803 | 18008 | 12400 | 4994 |
| **cg21195120** | ***MBTPS2*** | **X** | **21768225** | **7** | 15311 | 5316 | **52** | **91** | 22682 | 22682 |
| cg04689061 | *PKIA* | 8 | 79590548 | **8** | 3402 | 2709 | 9096 | 1515 | 16142 | 1610 |
| cg21614638 | *DAPP1* | 4 | 100956844 | **9** | 4521 | 18323 | 13834 | 10459 | 3990 | 3294 |
| cg02639007 | *CCDC41* | 12 | 93377476 | **10** | 18618 | 8703 | 7351 | 616 | 19561 | 304 |
| cg15700739 | *HOXC5* | 12 | 52713967 | **11** | 2547 | 4497 | 16959 | 231 | 2566 | 10075 |
| cg12148581 | *RPL14* | 3 | 40473347 | **12** | 22035 | 3855 | 6386 | 21390 | 20213 | 1003 |
| cg25118574 | *PSMB7* | 9 | 126217467 | **13** | 3414 | 12704 | 323 | 5579 | 20875 | 986 |
| cg06284322 | *TAF7* | 5 | 140680846 | **14** | 15115 | 5315 | 4144 | 3301 | 3459 | 5850 |
| cg23397015 | *INHBB* | 2 | 120818902 | **15** | 21957 | 4334 | 7008 | 2119 | 16886 | 1970 |
| cg12241297 | *HNRPA0* | 5 | 137118306 | **16** | 11040 | 516 | 22036 | 488 | 3582 | 3367 |
| cg13588354 | *MC3R* | 20 | 54256710 | **17** | 2688 | 20684 | 22768 | 735 | 9056 | 8483 |
| cg10528989 | *BDKRB1* | 14 | 95792059 | **18** | 14217 | 9330 | 23399 | 675 | 3578 | 18211 |
| cg15836394 | *FDFT1* | 8 | 11697241 | **19** | 4844 | 10642 | 335 | 1517 | 16920 | 4652 |
| cg03660451 | *RECQL5* | 17 | 131920213 | **20** | 17869 | 18070 | 16086 | 4161 | 12947 | 352 |
| **cg21935083** | ***RAD50*** | **5** | **71176201** | **21** | **52** | 1656 | 3315 | 1212 | 795 | 4776 |
| cg16166399 | *ZNF499* | 19 | 63722565 | **22** | 8581 | 14998 | 15079 | 4377 | 3126 | 1335 |
| cg23627134 | *ARHGAP15* | 2 | 143602845 | **23** | 5208 | 1015 | 3632 | 4066 | 17117 | 9786 |
| **cg05751148** | ***PTPRCAP*** | **11** | **66961375** | **24** | 12766 | 11681 | 14167 | 369 | **46** | 16646 |
| **cg18284523** | ***TINF2*** | **14** | **23781402** | **25** | 13419 | 8095 | 10212 | 13207 | 7265 | **30** |
| cg20346096 | *C18orf22* | 18 | 75895818 | **26** | 2922 | 657 | 4802 | 19637 | 3092 | 17710 |
| cg07200280 | *RAFTLIN* | 3 | 16529623 | **27** | 21276 | 10667 | 12051 | 14459 | 651 | 1703 |
| cg12624641 | *C14orf143* | 14 | 89490322 | **28** | 9156 | 19565 | 8386 | 2155 | 7454 | 2815 |
| **cg01253545** | ***RNF185*** | **22** | **29886314** | **29** | 10296 | 6538 | 1305 | 4994 | 2485 | **31** |
| cg17367215 | *SUPT5H* | 19 | 44628460 | **30** | 7863 | 20914 | 1013 | 221 | 10997 | 1256 |
| cg01894895 | *ANXA1* | 9 | 74956114 | **31** | 18632 | 22641 | 13245 | 1309 | 604 | 5125 |
| cg18776056 | *FKBP4* | 12 | 79668575 | **32** | 11221 | 4571 | 8389 | 105 | 884 | 10471 |
| cg20917484 | *GAPDH* | 12 | 6514327 | **33** | 20016 | 18499 | 12911 | 7916 | 961 | 4486 |
| cg24687764 | *C16orf46* | 16 | 2775173 | **34** | 8781 | 2393 | 13686 | 16070 | 15566 | 1647 |
| cg19674669 | *LOC112937* | 11 | 133652120 | **35** | 5917 | 16983 | 2545 | 151 | 139 | 4511 |
| **cg03875195** | ***SLC30A3*** | **2** | **27339867** | **36** | 8541 | 6216 | 20625 | 410 | 14792 | **80** |
| cg05520656 | *ZNF681* | 19 | 23733431 | **37** | 4371 | 5786 | 460 | 17754 | 3928 | 13304 |
| cg18515587 | *SELENBP1* | 1 | 149611430 | **38** | 5400 | 13064 | 5273 | 14307 | 805 | 15568 |
| cg07239938 | *ELA2* | 19 | 803813 | **39** | 2492 | 7045 | 164 | 6607 | 379 | 20716 |
| cg01148741 | *DUSP2* | 2 | 96175180 | **40** | 10997 | 3490 | 4706 | 11708 | 10163 | 2665 |
| cg17002259 | *CDC42SE1* | 1 | 149298884 | **41** | 3118 | 2161 | 15045 | 129 | 1609 | 112 |
| cg02154186 | *PNMA2* | 8 | 26427270 | **42** | 23058 | 15599 | 13192 | 1206 | 6033 | 1858 |
| cg18482268 | *POU4F3* | 5 | 145699158 | **43** | 22672 | 16502 | 3432 | 16318 | 19051 | 425 |
| cg20969846 | *DIDO1* | 20 | 61040666 | **44** | 9277 | 19361 | 21536 | 2531 | 4489 | 262 |
| cg00347904 | *SCUBE3* | 6 | 35290486 | **45** | 22362 | 13528 | 6892 | 4169 | 13559 | 16699 |
| cg26799474 | *CASP8* | 2 | 201807196 | **46** | 10977 | 11106 | 1149 | 191 | 1721 | 10264 |
| cg07584959 | *THRAP5* | 19 | 843937 | **47** | 342 | 19392 | 14470 | 337 | 14872 | 2740 |
| **cg19235307** | ***MBD4*** | **3** | **130642844** | **48** | 185 | 23461 | 13191 | 131 | 22523 | **35** |
| cg11861730 | *ETS1* | 11 | 127897893 | **49** | 8569 | 10065 | 15066 | 5975 | 3006 | 3948 |
| cg21410991 | *ISL1* | 5 | 50714208 | **50** | 21902 | 2875 | 1796 | 3411 | 2199 | 10240 |

| ProbeID | Gene | Chr | Position | Rank from Analytical Group* | | | | | | |
|---|---|---|---|---|---|---|---|---|---|---|
| | | | | **2** | **1** | **3** | **4** | **5** | **6** | **7** |
| cg15089487 | *THAP10* | 15 | 68972306 | **1** | 7174 | 19256 | 10245 | 1128 | 13852 | 125 |
| cg11324740 | *OIP5* | 15 | 39412085 | **2** | 7401 | 21067 | 13993 | 1181 | 2456 | 10903 |
| cg15240064 | *ZNF12* | 7 | 6713362 | **3** | 1596 | 1882 | 3952 | 301 | 10173 | 20951 |
| cg22226839 | *ATP2B4* | 1 | 152938352 | **4** | 4983 | 7996 | 22504 | 12287 | 8972 | 21067 |
| cg01353347 | *IRAK1* | X | 201862674 | **5** | 23137 | 21487 | 5569 | 614 | 22711 | 22711 |
| cg21241823 | *PRDM15* | 21 | 42173093 | **6** | 19758 | 20017 | 1513 | 133 | 17982 | 229 |
| cg15940569 | *GABRB3* | 15 | 24570449 | **7** | 17151 | 23100 | 10228 | 331 | 1604 | 12857 |
| cg11909310 | *DKFZP686A10121* | 7 | 89813722 | **8** | 7026 | 21108 | 14279 | 543 | 14422 | 13561 |
| cg12736438 | *RAB20* | 13 | 110012458 | **9** | 21192 | 10625 | 11131 | 2719 | 21008 | 19190 |
| cg01041367 | *TMEM70* | 8 | 75050634 | **10** | 21233 | 19658 | 12136 | 2270 | 19241 | 11556 |
| cg04244987 | *NCKIPSD* | 3 | 48698642 | **11** | 10000 | 19283 | 7183 | 1763 | 4430 | 14488 |
| **cg18349258** | ***SMEK2*** | **2** | **55697707** | **12** | 2792 | 1468 | 3125 | **2** | 17353 | 2014 |
| cg11492856 | *CAPN7* | 3 | 15221591 | **13** | 11033 | 3420 | 416 | 22414 | 8878 | 3077 |
| cg15995002 | *RAD54B* | 8 | 95556400 | **14** | 19773 | 17127 | 20489 | 1148 | 18266 | 9580 |
| cg01798443 | *RPSA* | 3 | 39422895 | **15** | 14827 | 22457 | 11823 | 2620 | 6073 | 22251 |
| cg24034289 | *SCAMP3* | 1 | 153498968 | **16** | 5599 | 4406 | 4122 | 3139 | 7728 | 773 |
| **cg25948180** | ***PDHX*** | **11** | **34893357** | **17** | 1279 | 8636 | 500 | **14** | 322 | 2919 |
| **cg19837131** | ***PIK3C3*** | **18** | **37789059** | **18** | 197 | 3060 | 118 | **1** | 17087 | 2143 |
| cg27537561 | *VDR* | 12 | 63849263 | **19** | 1912 | 5182 | 14895 | 1256 | 2826 | 1174 |
| cg11814446 | *LEMD3* | 12 | 46584923 | **20** | 16158 | 6181 | 12684 | 8205 | 5143 | 13286 |
| **cg01160766** | ***RPL38*** | **17** | **69711466** | **21** | 9128 | 4826 | 22877 | **68** | 15813 | 15223 |
| cg18855178 | *PCSK4* | 19 | 1441194 | **22** | 12098 | 18251 | 7200 | 3406 | 11318 | 7620 |
| cg08785133 | *PORCN* | X | 48252137 | **23** | 16895 | 19683 | 14146 | 3780 | 22856 | 22856 |
| cg13240311 | *PKIG* | 20 | 42594209 | **24** | 8479 | 14867 | 8258 | 1857 | 15116 | 18157 |
| cg20663980 | *CDX4* | X | 72583795 | **25** | 17892 | 4144 | 1648 | 18391 | 23344 | 23344 |
| **cg27198824** | ***AFF2*** | **X** | **147390419** | **26** | 9166 | **25** | 10711 | **4** | 22679 | 22679 |
| cg05857825 | *MAPRE1* | 20 | 30871874 | **27** | 3562 | 18790 | 18376 | 17191 | 15549 | 6129 |
| cg21509097 | *LYPLA3* | 16 | 66837114 | **28** | 11869 | 9268 | 13102 | 11609 | 7192 | 11522 |
| cg02345317 | *NLGN3* | X | 70281268 | **29** | 13475 | 14535 | 20435 | 1593 | 22749 | 22749 |
| cg16468910 | *SNX4* | 3 | 126721481 | **30** | 113 | 6845 | 3420 | 189 | 2054 | 7641 |
| cg14751914 | *SMAD7* | 18 | 44731402 | **31** | 210 | 17760 | 7188 | 6894 | 6174 | 10928 |
| cg25095380 | *PRDM5* | 4 | 122062657 | **32** | 13949 | 9414 | 23043 | 7744 | 6191 | 14898 |
| **cg04856685** | ***MAP4*** | **3** | **48105803** | **33** | 12480 | 499 | 11396 | **23** | 3134 | 14254 |
| cg19695867 | *WDR47* | 1 | 109386913 | **34** | 14245 | 18387 | 14798 | 2547 | 835 | 628 |
| cg13192155 | *ERAS* | X | 48570887 | **35** | 15620 | 282 | 4738 | 664 | 22714 | 22714 |
| **cg11223864** | ***GNB2*** | **7** | **100109259** | **36** | 6303 | 3467 | 259 | **5** | 3638 | 10436 |
| cg17150465 | *SCRIB* | 8 | 144969546 | **37** | 19646 | 5879 | 4771 | 4365 | 19261 | 15286 |
| cg05471775 | *HIST1H4B* | 6 | 26135510 | **38** | 7025 | 4572 | 9709 | 18223 | 8239 | 4750 |
| cg04959788 | *PTCH2* | 1 | 45081216 | **39** | 7500 | 10758 | 5930 | 7232 | 20306 | 7416 |
| cg26757053 | *LYPLAL1* | 1 | 78609397 | **40** | 3326 | 616 | 1180 | 1436 | 5157 | 11284 |
| cg14272175 | *PXK* | 3 | 58294000 | **41** | 8181 | 1193 | 19445 | 480 | 12977 | 16363 |
| cg17758148 | *JMY* | 5 | 217413644 | **42** | 9336 | 19624 | 952 | 1276 | 11726 | 6085 |
| cg00620629 | *C6orf113* | 6 | 117096644 | **43** | 157 | 13502 | 16856 | 139 | 9726 | 17276 |
| **cg07118638** | ***THEX1*** | **8** | **8897611** | **44** | 1314 | 3716 | 6718 | **18** | 20071 | 18450 |
| **cg19724470** | ***CD274*** | **9** | **5440936** | **45** | 8430 | 844 | 20839 | **95** | 3311 | 178 |
| cg05158538 | *PPARGC1A* | 4 | 23500584 | **46** | 5422 | 16631 | 273 | 6263 | 8194 | 15776 |
| cg26365553 | *MADD* | 11 | 47247189 | **47** | 9083 | 3435 | 5191 | 6888 | 16252 | 16329 |
| cg13585240 | *ARIH2* | 3 | 48934379 | **48** | 12712 | 4132 | 10257 | 1504 | 15926 | 7518 |
| **cg04156850** | ***GRB2*** | **17** | **70914167** | **49** | 17366 | 1548 | 4749 | **35** | 12817 | 7435 |
| cg24130043 | *ZNF197* | 3 | 44641366 | **50** | 3867 | 13955 | 15978 | 11537 | 9648 | 15284 |

| ProbeID | Gene | Chr | Position | Rank from Analytical Group* | | | | | | |
|---|---|---|---|---|---|---|---|---|---|---|
| | | | | **3** | **1** | **2** | **4** | **5** | **6** | **7** |
| cg16746631 | *IMPA1* | 8 | 82760695 | **1** | 7183 | 17140 | 22864 | 2070 | 12848 | 5330 |
| cg04624659 | *SPAG17* | 1 | 118529340 | **2** | 3383 | 9682 | 21607 | 7465 | 13242 | 11207 |
| cg27372468 | *SLC22A4* | 5 | 131658111 | **3** | 1095 | 10113 | 15611 | 12123 | 16389 | 3260 |
| cg16208448 | *TPP2* | 13 | 102047992 | **4** | 3944 | 21259 | 2859 | 9695 | 12597 | 7585 |
| cg18215716 | *TMEM111* | 3 | 10004246 | **5** | 7635 | 1057 | 13487 | 3224 | 19411 | 7837 |
| cg05107152 | *KLC4* | 6 | 43134752 | **6** | 1531 | 5823 | 4156 | 6910 | 11380 | 729 |
| cg04062907 | *ANAPC7* | 12 | 109325928 | **7** | 15205 | 730 | 11363 | 20111 | 21462 | 8417 |
| cg12930602 | *DIPA* | 11 | 65414471 | **8** | 17586 | 8257 | 11587 | 238 | 5983 | 10827 |
| cg24110050 | *TCTEX1D1* | 1 | 66990441 | **9** | 13991 | 3735 | 22127 | 3830 | 11943 | 18459 |
| cg13700897 | *RSPO2* | 8 | 109165240 | **10** | 19644 | 3678 | 12097 | 3287 | 18224 | 21814 |
| cg02210123 | *RHOJ* | 14 | 62740588 | **11** | 10736 | 13363 | 4402 | 497 | 219 | 19183 |
| cg02597128 | *CXorf41* | X | 106336479 | **12** | 9377 | 21575 | 16204 | 1054 | 22729 | 22729 |
| cg16082125 | *USP11* | X | 46977504 | **13** | 13100 | 21884 | 3202 | 1018 | 22726 | 22726 |
| cg08463061 | *RND3* | 2 | 151052578 | **14** | 5281 | 17052 | 8646 | 1917 | 6277 | 1207 |
| cg16541031 | *IRF7* | 11 | 605519 | **15** | 17608 | 6469 | 6157 | 14631 | 14529 | 14930 |
| **cg11738543** | ***SOCS2*** | **12** | **92491356** | **16** | 15198 | 1478 | 15991 | **85** | 310 | 1073 |
| **cg09665351** | ***APXL*** | **X** | **9715283** | **17** | 9509 | 7640 | 2720 | **33** | 22681 | 22681 |
| cg16290693 | *SPSB1* | 1 | 9275539 | **18** | 4701 | 5950 | 12174 | 3227 | 11144 | 15882 |
| cg10159529 | *IL5RA* | 3 | 3127530 | **19** | 5766 | 12117 | 12443 | 12179 | 21499 | 11710 |
| cg25909811 | *KDELR2* | 7 | 6489778 | **20** | 6296 | 10975 | 12871 | 1015 | 10636 | 15519 |
| cg01808130 | *SLC35B3* | 6 | 8380205 | **21** | 2438 | 654 | 1651 | 11847 | 22525 | 3400 |
| cg14155482 | *PIP5K1A* | 1 | 149437706 | **22** | 18258 | 15579 | 10644 | 1359 | 1565 | 14233 |
| **cg22492966** | ***JMJD1C*** | **10** | **64698935** | **23** | 102 | 15890 | 6204 | **57** | 14178 | 4668 |
| **cg08142684** | ***TCP1*** | **6** | **160129858** | **24** | **4** | 3663 | 1050 | **12** | 18711 | 4192 |
| **cg27198824** | ***AFF2*** | **X** | **151815702** | **25** | 9166 | **26** | 10711 | **4** | 22679 | 22679 |
| cg10274830 | *C6orf96* | 6 | 147390419 | **26** | 8888 | 7179 | 5928 | 5548 | 14288 | 6743 |
| cg05459203 | *NUDCD3* | 7 | 44497199 | **27** | 121 | 10892 | 15309 | 128 | 20728 | 16523 |
| cg27631817 | *OFCC1* | 6 | 10168924 | **28** | 14089 | 16897 | 8251 | 1151 | 7184 | 6541 |
| cg23828595 | *PRKG1* | 10 | 52503616 | **29** | 9427 | 1076 | 11209 | 12713 | 13937 | 18475 |
| cg18506672 | *SNURF* | 15 | 22751346 | **30** | 2411 | 9442 | 21405 | 9057 | 7713 | 9510 |
| cg07623294 | *ELAVL2* | 9 | 23816507 | **31** | 19098 | 11225 | 12337 | 5942 | 10162 | 13921 |
| cg13181019 | *MPP7* | 10 | 28611588 | **32** | 13186 | 11905 | 1317 | 11806 | 2465 | 22250 |
| cg06618866 | *TLR2* | 4 | 154824537 | **33** | 13257 | 19821 | 12086 | 4740 | 10252 | 9956 |
| cg23653712 | *SGCB* | 4 | 52599136 | **34** | 483 | 11503 | 149 | 12128 | 16919 | 322 |
| cg17746675 | *C3orf31* | 3 | 11863297 | **35** | 11768 | 14424 | 4467 | 6485 | 21309 | 13045 |
| cg02200584 | *PDGFC* | 4 | 158111996 | **36** | 14731 | 6192 | 531 | 18387 | 12773 | 1654 |
| **cg12542604** | ***ANKS1A*** | **6** | **34964682** | **37** | 6626 | 490 | 6565 | **11** | 4144 | **99** |
| cg02171545 | *SNRPN* | 15 | 22644459 | **38** | 19981 | 9936 | 3789 | 2772 | 19680 | 5658 |
| cg14967972 | *PANK1* | 10 | 91395245 | **39** | 1042 | 15947 | 4904 | 1747 | 785 | 250 |
| cg17412258 | *DLK1* | 14 | 100262770 | **40** | 16442 | 22926 | 5578 | 7792 | 2075 | 9363 |
| cg02712845 | *DPAGT1* | 11 | 118478248 | **41** | 1185 | 3600 | 4519 | 130 | 3961 | 18803 |
| **cg20902737** | ***EFTUD2*** | **17** | **40331968** | **42** | 22223 | 688 | 1400 | **30** | 18138 | 10059 |
| cg11692477 | *SLC40A1* | 2 | 190153722 | **43** | 13906 | 5234 | 9152 | 17118 | 9118 | 17066 |
| cg00468146 | *ID4* | 6 | 19946190 | **44** | 1962 | 8150 | 8771 | 12007 | 18874 | 4365 |
| cg09053680 | *UTF1* | 10 | 134894104 | **45** | 9910 | 2303 | 5410 | 5672 | 8499 | 2719 |
| cg06905514 | *CAMK2B* | 7 | 44331466 | **46** | 15011 | 17382 | 12010 | 1962 | 13496 | 9863 |
| cg24921089 | *AMPD3* | 11 | 10429416 | **47** | 22787 | 16214 | 1216 | 12458 | 5478 | 5763 |
| cg03863149 | *ZNF644* | 1 | 91260104 | **48** | 14129 | 9009 | 13044 | 7508 | 13275 | 3631 |
| **cg15494458** | ***BPI*** | **20** | **36364926** | **49** | 611 | 17132 | 15518 | **46** | 15911 | 3013 |
| cg26491425 | *RHOV* | 15 | 38953924 | **50** | 16027 | 12200 | 18848 | 4763 | 1676 | 11158 |

| ProbeID | Gene | Chr | Position | Rank from Analytical Group* | | | | | | |
|---|---|---|---|---|---|---|---|---|---|---|
| | | | | **4** | **1** | **2** | **3** | **5** | **6** | **7** |
| cg16399745 | *CNAP1* | 12 | 6474300 | **1** | 7756 | 20731 | 5267 | 6374 | 10350 | 16217 |
| cg06340713 | *CHM* | X | 85190083 | **2** | 432 | 1434 | 17991 | 18997 | 23368 | 23368 |
| cg12237946 | *PGBD4* | 15 | 32181668 | **3** | 7100 | 18837 | 12687 | 1851 | 11263 | 8209 |
| cg02245418 | *ZNF364* | 1 | 144321032 | **4** | 1571 | 14604 | 11022 | 7615 | 4311 | 17265 |
| cg25040783 | *APPL* | 3 | 57236346 | **5** | 5763 | 14866 | 11699 | 3601 | 17925 | 16936 |
| cg05881762 | *UBE3A* | 15 | 23235942 | **6** | 14629 | 12104 | 4886 | 378 | 1750 | 6927 |
| cg04578090 | *PROCA1* | 17 | 24063650 | **7** | 7620 | 10604 | 13146 | 366 | 16337 | 5779 |
| cg22947000 | *BCMO1* | 16 | 79829782 | **8** | 6377 | 374 | 6085 | 22868 | 15433 | 18479 |
| **cg24371383** | ***CEP55*** | **10** | **95246094** | **9** | 2343 | 9005 | 4749 | **17** | 6091 | 3326 |
| **cg22232206** | ***SRF*** | **6** | **43246163** | **10** | 5739 | 4090 | 13546 | **45** | 728 | 6740 |
| cg24073051 | *CDV3* | 3 | 134774669 | **11** | 1204 | 7262 | 3163 | 2101 | 18569 | 9928 |
| **cg01485998** | ***FLJ12505*** | **1** | **211190520** | **12** | 19781 | 284 | 5609 | **15** | 14675 | 19465 |
| cg00662775 | *TCEAL4* | X | 102727091 | **13** | 3162 | 17859 | 18398 | 655 | 22713 | 22713 |
| cg23054437 | *MOSC2* | 1 | 218987929 | **14** | 900 | 7823 | 14179 | 4938 | 6208 | 14299 |
| cg00901652 | *SH2BP1* | 11 | 10729010 | **15** | 10780 | 18228 | 17292 | 1074 | 17465 | 7723 |
| **cg23839680** | ***CCT6A*** | **7** | **56085732** | **16** | 12751 | 363 | 1577 | **13** | 7150 | 7695 |
| **cg09580336** | ***ATP1A1*** | **1** | **116716645** | **17** | 13158 | 890 | 3449 | **81** | 9946 | 5280 |
| cg14196790 | *SLC22A5* | 5 | 131732934 | **18** | 4755 | 7149 | 10652 | 12281 | 18746 | 20912 |
| cg04902405 | *ZC3H11A* | 1 | 202035140 | **19** | 7110 | 21322 | 17000 | 4105 | 11891 | 12525 |
| cg11392765 | *BAPX1* | 4 | 13155771 | **20** | 147 | 1581 | 21607 | 15382 | 2389 | 4248 |
| cg05419984 | *PIGX* | 3 | 197922860 | **21** | 15866 | 21354 | 17925 | 3333 | 21398 | 12648 |
| cg02620013 | *MLNR* | 13 | 48692682 | **22** | 20963 | 3900 | 4343 | 4393 | 4918 | 12097 |
| cg20649047 | *NUMB* | 14 | 72994914 | **23** | 9191 | 5353 | 14724 | 23237 | 10714 | 14063 |
| cg00325491 | *FN5* | 11 | 92925818 | **24** | 438 | 2393 | 21653 | 4130 | 13124 | 3462 |
| cg26536259 | *BMP2* | 20 | 6696006 | **25** | 20776 | 2288 | 8345 | 1388 | 1548 | 2545 |
| cg04145477 | *QRSL1* | 6 | 107184322 | **26** | 20401 | 11955 | 4026 | 977 | 13493 | 9997 |
| cg13306784 | *INPP5E* | 9 | 138454309 | **27** | 18537 | 19929 | 20846 | 1887 | 7422 | 1843 |
| **cg10521267** | ***ZNF684*** | **1** | **40769714** | **28** | 9963 | 149 | 5772 | **56** | 21795 | 12563 |
| **cg01909833** | ***BRD2*** | **6** | **33045245** | **29** | 878 | 18254 | 10661 | **82** | 8583 | 21268 |
| cg27170298 | *BCS1L* | 2 | 219233031 | **30** | 21113 | 6569 | 4368 | 253 | 21065 | 16448 |
| cg12073779 | *CRYGD* | 2 | 208697714 | **31** | 20960 | 4928 | 19766 | 8982 | 13887 | 12522 |
| cg05944800 | *INSIG2* | 2 | 118562016 | **32** | 13365 | 1606 | 13658 | 1662 | 2610 | 1358 |
| **cg26083396** | ***IMPDH1*** | **7** | **127837121** | **33** | 3356 | 6631 | 19681 | **76** | 3809 | 14918 |
| cg05062178 | *ITGB5* | 3 | 126087937 | **34** | 15711 | 20911 | 13078 | 3612 | 1690 | 7430 |
| cg11395610 | *RAB28* | 4 | 13094336 | **35** | 20667 | 17603 | 6707 | 11072 | 13538 | 21848 |
| cg04422896 | *C12orf43* | 12 | 119938652 | **36** | 14754 | 18841 | 22541 | 3905 | 18762 | 14159 |
| **cg26675382** | ***NUP43*** | **6** | **150109539** | **37** | 5339 | 8531 | 17191 | 475 | **18** | 14561 |
| cg05768141 | *KCNJ10* | 1 | 158306471 | **38** | 3475 | 3980 | 17031 | 16781 | 10717 | 2018 |
| cg12179176 | *SNX19* | 11 | 130291765 | **39** | 2485 | 18407 | 16801 | 10575 | 17261 | 19035 |
| cg25921910 | *CDC73* | 1 | 191357280 | **40** | 1832 | 19593 | 13603 | 894 | 15885 | 10027 |
| cg17572791 | *HAX1* | 1 | 152511809 | **41** | 5967 | 9829 | 1636 | 18734 | 2005 | 3855 |
| cg04587910 | *XLF* | 2 | 219733988 | **42** | 2893 | 107 | 16285 | 2212 | 19262 | 175 |
| cg19848683 | *EVI1* | 3 | 170346742 | **43** | 9504 | 1067 | 18777 | 113 | 7197 | 16385 |
| cg24456340 | *GNGT2* | 17 | 44641482 | **44** | 13030 | 11057 | 17756 | 3325 | 10706 | 2197 |
| **cg08513100** | ***UBE2B*** | **5** | **133734621** | **45** | 7769 | 14242 | 163 | **83** | 15842 | 18816 |
| cg15182360 | *PCDH20* | 13 | 60887738 | **46** | 14789 | 13642 | 20683 | 635 | 14182 | 13777 |
| cg20716209 | *STAT3* | 17 | 37794796 | **47** | 2010 | 4524 | 15840 | 1405 | 5579 | 3698 |
| cg12917695 | *HSPC268* | 7 | 138675699 | **48** | 10982 | 18573 | 22901 | 1435 | 15513 | 4983 |
| cg23062876 | *DBT* | 1 | 100488055 | **49** | 13107 | 19982 | 2633 | 463 | 14140 | 14433 |
| cg14800883 | *ARPC4* | 3 | 9809004 | **50** | 2182 | 12089 | 19023 | 345 | 292 | 12962 |

| ProbeID | Gene | Chr | Position | Rank from Analytical Group* | | | | | | |
|---|---|---|---|---|---|---|---|---|---|---|
| | | | | **5** | **1** | **2** | **3** | **4** | **6** | **7** |
| **cg19837131** | ***PIK3C3*** | **18** | **37789059** | **1** | 197 | **18** | 3060 | 118 | 17087 | 2143 |
| **cg18349258** | ***SMEK2*** | **2** | **55697707** | **2** | 2792 | **12** | 1468 | 3125 | 17353 | 2014 |
| cg12478185 | *SCO1* | 17 | 10542726 | **3** | 923 | 2431 | 626 | 311 | 8389 | 9713 |
| **cg27198824** | ***AFF2*** | **X** | **147390419** | **4** | 9166 | **26** | **25** | 10711 | 22679 | 22679 |
| **cg11223864** | ***GNB2*** | **7** | **100109259** | **5** | 6303 | **36** | 3467 | 259 | 3638 | 10436 |
| cg21926138 | *C1orf88* | 1 | 111690943 | **6** | 1284 | 306 | 419 | 11839 | 12738 | 21093 |
| cg25095951 | *RPS26* | 12 | 54721514 | **7** | 783 | 776 | 194 | 12445 | 17036 | 13383 |
| cg04961553 | *OCIAD2* | 4 | 48603226 | **8** | 143 | 218 | 1456 | 3405 | 1664 | 11019 |
| **cg19318511** | ***C9orf30*** | **9** | **102228979** | **9** | 1189 | **79** | 4698 | 13785 | 15523 | 1220 |
| cg23412850 | *SOCS2* | 12 | 92491518 | **10** | 1788 | 3862 | 1089 | 1630 | 11345 | 939 |
| **cg12542604** | ***ANKS1A*** | **6** | **34964682** | **11** | 6626 | 490 | **37** | 6565 | 4144 | **99** |
| **cg08142684** | ***TCP1*** | **6** | **160129858** | **12** | **4** | 3663 | **24** | 1050 | 18711 | 4192 |
| **cg23839680** | ***CCT6A*** | **7** | **56085732** | **13** | 12751 | 363 | 1577 | **16** | 7150 | 7695 |
| **cg25948180** | ***PDHX*** | **11** | **34893357** | **14** | 1279 | **17** | 8636 | 500 | 322 | 2919 |
| **cg01485998** | ***FLJ12505*** | **1** | **211190520** | **15** | 19781 | 284 | 5609 | **12** | 14675 | 19465 |
| cg08729012 | *CMTM6* | 3 | 32518632 | **16** | 1225 | 191 | 504 | 20788 | 5967 | 3491 |
| **cg24371383** | ***CEP55*** | **10** | **95246094** | **17** | 2343 | 9005 | 4749 | **9** | 6091 | 3326 |
| **cg07118638** | ***THEX1*** | **8** | **8897611** | **18** | 1314 | **44** | 3716 | 6718 | 20071 | 18450 |
| cg24364574 | *PREX1* | 20 | 46877101 | **19** | 7480 | 569 | 12268 | 263 | 5751 | 10959 |
| cg12623088 | *MGC4562* | 15 | 64372575 | **20** | 15209 | 1162 | 2794 | 113 | 11920 | 12805 |
| cg05815906 | *RAB33B* | 4 | 140593788 | **21** | 489 | 276 | 12486 | 169 | 15733 | 3113 |
| cg25890048 | *OR5I1* | 11 | 48105803 | **22** | 260 | 187 | 5412 | 6307 | 3002 | 4113 |
| **cg04856685** | ***MAP4*** | **3** | **55460019** | **23** | 12480 | **33** | 499 | 11396 | 3134 | 14254 |
| cg14663065 | *CTNNB1* | 3 | 41216634 | **24** | 2805 | 298 | 1178 | 5973 | 8695 | 6714 |
| cg11164400 | *PPP1R9A* | 7 | 94374723 | **25** | 13371 | 528 | 1231 | 1880 | 21882 | 9941 |
| cg05072951 | *CAPN1* | 11 | 64705974 | **26** | 3441 | 990 | 9472 | 372 | 9331 | 3312 |
| cg11810837 | *ARMCX5* | X | 101740932 | **27** | 2576 | 11138 | 156 | 1282 | 22680 | 22680 |
| cg18710985 | *C20orf23* | 20 | 16502249 | **28** | 19640 | 6163 | 113 | 277 | 4988 | 11659 |
| cg05486551 | *FLJ25067* | 20 | 5679259 | **29** | 2315 | 117 | 5217 | 4208 | 2093 | 10528 |
| **cg20902737** | ***EFTUD2*** | **17** | **40331968** | **30** | 22223 | 688 | **42** | 1400 | 18138 | 10059 |
| cg21006686 | *NOS1* | 12 | 116284623 | **31** | 13782 | 602 | 1023 | 3975 | 6078 | 6213 |
| **cg17398595** | ***SH3GL2*** | **9** | **17568725** | **32** | 10626 | 11268 | 848 | **91** | 16541 | 6677 |
| **cg09665351** | ***APXL*** | **X** | **9715283** | **33** | 9509 | 7640 | **17** | 2720 | 22681 | 22681 |
| **cg21829265** | ***ZNF451*** | **6** | **57019098** | **34** | **87** | 22318 | 3414 | 237 | 7456 | 880 |
| **cg04156850** | ***GRB2*** | **17** | **70914167** | **35** | 17366 | **49** | 1548 | 4749 | 12817 | 7435 |
| cg02441647 | *COL8A1* | 3 | 100840189 | **36** | 6433 | 829 | 2329 | 2999 | 21225 | 7733 |
| cg19430430 | *COL5A3* | 19 | 9982323 | **37** | 456 | 6528 | 3594 | 932 | 718 | 17584 |
| cg00250430 | *DMRT2* | 9 | 1041579 | **38** | 250 | 2034 | 3388 | 7114 | 804 | 5607 |
| cg15683488 | *ZFYVE9* | 1 | 52380059 | **39** | 1893 | 19451 | 197 | 1916 | 5112 | 21023 |
| cg23146358 | *CDKN1C* | 11 | 2862072 | **40** | 2665 | 145 | 12717 | 9474 | 2312 | 18858 |
| cg02337166 | *NR1D1* | 17 | 61649677 | **41** | 1992 | 690 | 20079 | 1127 | 17055 | 12197 |
| cg22442730 | *SRMS* | 20 | 35510829 | **42** | 3270 | 1014 | 4429 | 5153 | 19767 | 5536 |
| cg26251865 | *IRGC* | 19 | 57395452 | **43** | 5762 | 352 | 2167 | 9337 | 2654 | 15822 |
| cg13573276 | *RDHE2* | 8 | 48912054 | **44** | 453 | 3069 | 779 | 10367 | 4949 | 2129 |
| **cg22232206** | ***SRF*** | **6** | **43246163** | **45** | 5739 | 4090 | 13546 | **10** | 728 | 6740 |
| **cg15494458** | ***BPI*** | **20** | **36364926** | **46** | 611 | 17132 | **49** | 15518 | 15911 | 3013 |
| cg02787991 | *SECTM1* | 17 | 77884694 | **47** | 13762 | 1337 | 4362 | 381 | 10161 | 11472 |
| cg08228917 | *LHFP* | 13 | 39075062 | **48** | 11754 | 3770 | 343 | 1551 | 6168 | 21993 |
| cg05960806 | *CLNS1A* | 11 | 77026532 | **49** | 3950 | 259 | 2871 | 11557 | 17524 | 18004 |
| **cg08137040** | ***LOC168850*** | **7** | **126820931** | **50** | 165 | 18685 | 2134 | 4537 | **17** | 6583 |

| ProbeID | Gene | Chr | Position | Rank from Analytical Group* | | | | | | |
|---|---|---|---|---|---|---|---|---|---|---|
| | | | | 6 | 1 | 2 | 3 | 4 | 5 | 7 |
| cg16474696 | *MGC3207* | 19 | 13736014 | **1** | 4425 | 7862 | 11473 | 4512 | 20335 | 2511 |
| cg15379633 | *RAB36* | 22 | 21817586 | **2** | 2694 | 5168 | 726 | 6521 | 7136 | 16897 |
| cg17421623 | *C3orf9* | 3 | 120670260 | **3** | 23076 | 16303 | 5454 | 1381 | 20184 | 700 |
| cg23815491 | *HP* | 16 | 70646123 | **4** | 18837 | 6793 | 17595 | 12415 | 18265 | 2476 |
| cg14119236 | *FGF23* | 12 | 4359336 | **5** | 18195 | 10750 | 451 | 5333 | 4722 | 12370 |
| cg17408686 | *CHCHD6* | 3 | 127905265 | **6** | 2464 | 9931 | 7859 | 6152 | 6428 | 1938 |
| cg26029902 | *RAB22A* | 20 | 56317831 | **7** | 3418 | 22285 | 11340 | 275 | 7064 | 9292 |
| cg04743872 | *FLJ20701* | 2 | 229843821 | **8** | 13608 | 21303 | 18644 | 5673 | 8181 | 19673 |
| cg14329157 | *WDR69* | 2 | 228444379 | **9** | 4572 | 18260 | 2105 | 16537 | 1254 | 18929 |
| cg08909157 | *C9orf66* | 9 | 205561 | **10** | 15515 | 11623 | 19542 | 1085 | 8601 | 17412 |
| cg14920334 | *GALNT8* | 12 | 4700088 | **11** | 4278 | 2145 | 8530 | 2046 | 22620 | 2551 |
| cg22584138 | *SLC6A4* | 17 | 25586346 | **12** | 7250 | 23132 | 2565 | 14466 | 6379 | 15828 |
| cg21717724 | *PSMD5* | 9 | 122644335 | **13** | 4555 | 3808 | 8601 | 8070 | 18458 | 1466 |
| cg22778947 | *FSD1NL* | 9 | 107250885 | **14** | 6422 | 21518 | 11671 | 20861 | 9935 | 17838 |
| cg13620770 | *BAD* | 11 | 63808314 | **15** | 1410 | 4635 | 11615 | 2045 | 17579 | 8186 |
| cg08598221 | *SNTB1* | 8 | 121894110 | **16** | 11440 | 21085 | 11216 | 12147 | 14768 | 714 |
| **cg08137040** | ***LOC168850*** | **7** | **126820931** | **17** | 165 | 18685 | 2134 | 4537 | **50** | 6583 |
| **cg26675382** | ***NUP43*** | **6** | **150109539** | **18** | 5339 | 8531 | 17191 | **37** | 475 | 14561 |
| cg21226225 | *TXNRD1* | 12 | 103204872 | **19** | 23004 | 7018 | 19094 | 1618 | 1670 | 820 |
| cg19328294 | *PRSS22* | 16 | 2848555 | **20** | 18525 | 22367 | 18999 | 23090 | 23322 | 233 |
| cg06392241 | *NUDT4* | 12 | 92295477 | **21** | 22181 | 22968 | 14043 | 9708 | 18994 | 12196 |
| cg08942800 | *CRISP2* | 6 | 49789269 | **22** | 4925 | 22164 | 21674 | 12475 | 18566 | 11858 |
| cg26233209 | *ATG12* | 5 | 115206663 | **23** | 16714 | 2761 | 5046 | 17942 | 17481 | 5588 |
| cg08085267 | *C17orf57* | 17 | 42756832 | **24** | 3061 | 1574 | 5741 | 5648 | 21675 | 6550 |
| cg24424381 | *PEX11A* | 15 | 88034861 | **25** | 2783 | 11704 | 1393 | 22304 | 2767 | 21643 |
| cg20540428 | *PPP4R2* | 3 | 73128376 | **26** | 8606 | 8806 | 7649 | 3746 | 16748 | 6770 |
| cg26538349 | *FLJ20035* | 4 | 169476425 | **27** | 4379 | 13661 | 10404 | 3192 | 1082 | 17922 |
| cg20721467 | *MYST4* | 10 | 76267933 | **28** | 11168 | 1566 | 22082 | 8592 | 7103 | 19200 |
| **cg16970232** | ***APC*** | **5** | **203087** | **29** | 635 | 6088 | 2000 | 4075 | **87** | 5532 |
| cg02671171 | *RPH3AL* | 17 | 112101332 | **30** | 5545 | 18207 | 19476 | 16915 | 19354 | 12586 |
| cg12000587 | *C17orf79* | 17 | 27210743 | **31** | 13522 | 20326 | 11694 | 21685 | 12262 | 13675 |
| cg21911019 | *GPR78* | 4 | 8632550 | **32** | 4853 | 14264 | 2429 | 18072 | 2172 | 2841 |
| cg17945001 | *IGSF21* | 1 | 18306705 | **33** | 190 | 1676 | 3995 | 23400 | 11341 | 14026 |
| cg14056644 | *PITX2* | 4 | 111778554 | **34** | 18485 | 8099 | 17373 | 14333 | 18064 | 295 |
| cg07753644 | *P2RY11* | 19 | 10083175 | **35** | 9140 | 3256 | 12851 | 17925 | 7497 | 9421 |
| cg13030582 | *MFAP4* | 17 | 19231301 | **36** | 913 | 12746 | 10128 | 16700 | 9196 | 10508 |
| cg19384697 | *UPK3B* | 7 | 75977858 | **37** | 12478 | 23090 | 17823 | 22360 | 19443 | 12533 |
| cg13206017 | *SST* | 3 | 134947870 | **38** | 72 | 16820 | 292 | 4935 | 2859 | 18717 |
| cg24879335 | *TF* | 3 | 188870919 | **39** | 7907 | 8733 | 6709 | 18035 | 12992 | 14158 |
| cg20843052 | *KIAA1841* | 2 | 61147229 | **40** | 6257 | 22831 | 13751 | 20895 | 7661 | 10203 |
| cg19573166 | *SLC22A17* | 14 | 22892248 | **41** | 12959 | 6706 | 12565 | 6628 | 13625 | 21226 |
| cg13652336 | *DEPDC2* | 8 | 69026566 | **42** | 3073 | 23061 | 17414 | 17044 | 12506 | 2082 |
| cg09682183 | *UNC93A* | 6 | 167625243 | **43** | 11599 | 2847 | 9507 | 6695 | 19026 | 16632 |
| cg03330058 | *ABTB1* | 3 | 22355205 | **44** | 6678 | 19731 | 8129 | 1076 | 7847 | 10636 |
| cg18960218 | *SLC7A7* | 14 | 128875093 | **45** | 10442 | 13381 | 18205 | 5682 | 12998 | 2654 |
| cg05751148 | *PTPRCAP* | 11 | 66961375 | **46** | 24 | 12766 | 11681 | 14167 | 369 | 16646 |
| cg13735974 | *NFYC* | 1 | 40929567 | **47** | 1 | 18192 | 4985 | 17744 | 3700 | 12151 |
| cg26922202 | *OR2S2* | 9 | 35947984 | **48** | 8679 | 21041 | 11064 | 10422 | 5148 | 20663 |
| cg05535113 | *CHST4* | 16 | 70117080 | **49** | 562 | 21103 | 12176 | 1488 | 15907 | 10848 |
| cg15584813 | *SLC38A4* | 12 | 45505893 | **50** | 12183 | 9530 | 7242 | 2662 | 22758 | 1950 |

| ProbeID | Gene | Chr | Position | Rank from Analytical Group * | | | | | | |
|---|---|---|---|---|---|---|---|---|---|---|
| | | | | **7** | **1** | **2** | **3** | **4** | **5** | **6** |
| cg07665060 | *C19orf33* | 19 | 43486438 | **1** | 18961 | 16491 | 1260 | 3918 | 841 | 15261 |
| cg17006282 | *RPL36* | 19 | 5640943 | **2** | 8399 | 14281 | 15373 | 15382 | 6212 | 15091 |
| cg01353448 | *C7orf16* | 7 | 31693437 | **3** | 6797 | 1395 | 2048 | 17540 | 3930 | 18596 |
| cg09547224 | *SLC5A1* | 22 | 30769259 | **4** | 19953 | 15167 | 14191 | 1366 | 5907 | 5053 |
| **cg17571291** | ***BLVRA*** | **7** | **43764312** | **5** | 17722 | 5125 | 9599 | 16859 | 5631 | **98** |
| cg01511567 | *SSRP1* | 11 | 56860207 | **6** | 2292 | 8396 | 11564 | 8753 | 6183 | 3810 |
| cg00027083 | *EPB41L3* | 18 | 5533801 | **7** | 5384 | 1077 | 3295 | 13451 | 780 | 7480 |
| cg16685388 | *HIVEP3* | 1 | 42156643 | **8** | 6901 | 19832 | 13999 | 19171 | 21418 | 10421 |
| cg08020808 | *CMA1* | 14 | 24047613 | **9** | 12405 | 2408 | 17225 | 1070 | 11693 | 732 |
| cg14847483 | *TMEM85* | 15 | 32303932 | **10** | 12176 | 2427 | 1872 | 19521 | 12103 | 3857 |
| cg25434223 | *ELAVL3* | 19 | 11452491 | **11** | 8749 | 16340 | 18125 | 17301 | 13794 | 14075 |
| cg12437481 | *MRPL28* | 16 | 360113 | **12** | 19441 | 6516 | 8970 | 16548 | 21702 | 5954 |
| cg01294695 | *MESP1* | 15 | 88095644 | **13** | 2470 | 16855 | 22908 | 10001 | 18990 | 1628 |
| cg11126134 | *FLJ14834* | 13 | 30378304 | **14** | 7936 | 4219 | 1395 | 21578 | 7562 | 569 |
| cg11096993 | *ACY3* | 11 | 67174534 | **15** | 23164 | 13877 | 13068 | 17275 | 9717 | 9668 |
| cg14972143 | *EIF4E* | 4 | 100070026 | **16** | 11449 | 21622 | 1494 | 1779 | 22087 | 948 |
| cg17838516 | *MTNR1B* | 11 | 92343184 | **17** | 23239 | 22953 | 1703 | 9002 | 11459 | 2267 |
| cg18771300 | *RHOJ* | 14 | 3469616 | **18** | 20112 | 5235 | 20835 | 17879 | 14434 | 293 |
| cg21667836 | *PRMT8* | 12 | 62741490 | **19** | 869 | 5627 | 10781 | 22663 | 767 | 13552 |
| cg19668234 | *TSP50* | 3 | 46734453 | **20** | 9214 | 11160 | 6394 | 16371 | 9448 | 1894 |
| cg06131859 | *KYNU* | 2 | 143351601 | **21** | 14584 | 3890 | 9200 | 1639 | 2434 | 14197 |
| cg06148175 | *ACY3* | 11 | 67174724 | **22** | 22691 | 6165 | 17363 | 7236 | 4831 | 6299 |
| cg02630694 | *C10orf7* | 10 | 12276839 | **23** | 22669 | 1003 | 13346 | 22398 | 3240 | 707 |
| cg08040471 | *C17orf62* | 17 | 78001068 | **24** | 181 | 10950 | 551 | 13531 | 10452 | 1627 |
| cg20832020 | *VSIG9* | 3 | 115495602 | **25** | 4648 | 7391 | 17142 | 3264 | 5535 | 19906 |
| cg04384398 | *PMM1* | 22 | 40316279 | **26** | 12495 | 18520 | 19281 | 15367 | 9903 | 21112 |
| cg16357381 | *COL7A1* | 3 | 48607896 | **27** | 5470 | 15520 | 1984 | 15531 | 8770 | 12036 |
| cg25250358 | *PLOD2* | 3 | 147361774 | **28** | 17378 | 2421 | 14352 | 1025 | 435 | 3661 |
| cg18432105 | *MYH2* | 17 | 10394235 | **29** | 21350 | 18491 | 3581 | 15639 | 9456 | 11805 |
| **cg18284523** | ***TINF2*** | **14** | **23781402** | **30** | **25** | 13419 | 8095 | 10212 | 13207 | 7265 |
| **cg01253545** | ***RNF185*** | **22** | **29886314** | **31** | **29** | 10296 | 6538 | 1305 | 4994 | 2485 |
| cg00498305 | *SLC18A2* | 10 | 118990226 | **32** | 2412 | 7975 | 23395 | 16062 | 3852 | 5821 |
| cg05189291 | *ICF45* | 5 | 157091035 | **33** | 16704 | 1168 | 8155 | 5209 | 828 | 20105 |
| **cg22313024** | ***UQCRB*** | **8** | **97317245** | **34** | 224 | 20952 | 3835 | 624 | **59** | 11315 |
| **cg19235307** | ***MBD4*** | **3** | **130642844** | **35** | **48** | 185 | 23461 | 13191 | 131 | 22523 |
| cg10214058 | *CNOT8* | 5 | 154218362 | **36** | 1567 | 8336 | 5200 | 7145 | 16219 | 12613 |
| cg17749961 | *LYCAT* | 2 | 30523367 | **37** | 13367 | 1239 | 8100 | 10733 | 5854 | 8267 |
| cg08440425 | *LRRC51* | 11 | 71468383 | **38** | 22749 | 14377 | 10224 | 735 | 17173 | 131 |
| cg19404582 | *GRIN1* | 9 | 139152774 | **39** | 20086 | 8453 | 494 | 9601 | 8291 | 21416 |
| cg19408398 | *FIP1L1* | 4 | 53938085 | **40** | 1339 | 8659 | 22076 | 18740 | 3337 | 1174 |
| cg20663831 | *GIMAP2* | 7 | 150014087 | **41** | 1474 | 6195 | 18196 | 6877 | 7703 | 18089 |
| cg13234848 | *AUTS2* | 7 | 68701907 | **42** | 19897 | 17508 | 21035 | 2895 | 5612 | 3338 |
| cg13311440 | *CD48* | 1 | 158948028 | **43** | 20782 | 4243 | 18331 | 17792 | 17829 | 185 |
| cg14951292 | *HMOX2* | 16 | 4465987 | **44** | 15531 | 5652 | 15973 | 13940 | 16910 | 2844 |
| cg10562586 | *MAP2* | 2 | 210152399 | **45** | 775 | 15171 | 5672 | 15622 | 1289 | 12929 |
| cg03962522 | *SLC5A1* | 22 | 185300625 | **46** | 722 | 15312 | 6127 | 4400 | 20515 | 5298 |
| cg18236734 | *HTR3E* | 3 | 30769254 | **47** | 19500 | 9971 | 3990 | 10877 | 13038 | 725 |
| cg10784813 | *SOCS1* | 16 | 11256179 | **48** | 1764 | 1364 | 16658 | 13345 | 11728 | 10254 |
| cg18632631 | *TNK1* | 17 | 7224773 | **49** | 22485 | 13715 | 17751 | 1385 | 14256 | 1400 |
| cg07758574 | *C3orf62* | 3 | 49289374 | **50** | 420 | 19858 | 15319 | 157 | 467 | 17377 |
