# Supplementary material for: Methylomic analysis of monozygotic twins discordant for autism spectrum disorder and related behavioural traits
Source: Mol Psychiatry. 2013 Apr 23;19(4):495–503. doi: 10.1038/mp.2013.41 (PMC3906213; doi:10.1038/mp.2013.41)
Supplement: Supplementary Table 8 [file mp201341x8.doc]

| Rank list from analysis group* | 1 | 2 | 3 | 4 | 5 | 6 | 7 |
| --- | --- | --- | --- | --- | --- | --- | --- |
| 1 | 1 |  |  |  |  |  |  |
| 2 | 0.029 (p= 8.01e-06) | 1 |  |  |  |  |  |
| 3 | 0.034 (p=2.55E-07) | 0.042 (p=1.96E-10) | 1 |  |  |  |  |
| 4 | 0.031 (p=1.55E-06) | 0.025 (p=1.19E-04) | 0.041 (p=2.538E-10) | 1 |  |  |  |
| 5 | 0.156 (p<2.2E-16) | 0.199 (p< 2.2E-16) | 0.207 (p<2.2E-16) | 0.154 (p<2.2E-16) | 1 |  |  |
| 6 | 0.067 (p<2.2E-16) | 0.017 (p=8.54E-03) | 0.027 (p=4.339E-05) | 0.019 (p=3.12E-03) | 0.027 (p=3.14E-05) | 1 |  |
| 7 | 0.158 (p<2.2E-16) | 0.025 (p=1.10E-04) | 0.029 (p=1.048E-05) | 0.015 (p=2.13E-02) | 0.050 (p=2.27E-14) | 0.152 (p<2.2E-16) | 1 |
