# Supplementary material for: Methylomic analysis of monozygotic twins discordant for autism spectrum disorder and related behavioural traits
Source: Mol Psychiatry. 2013 Apr 23;19(4):495–503. doi: 10.1038/mp.2013.41 (PMC3906213; doi:10.1038/mp.2013.41)
Supplement: Supplementary Table 10 [file mp201341x10.pdf]

Twin 1	Twin 2	CNV locus*	Genes	No. Probes	Length (bp) **	Type	Previous reports of ASD-associated CNVs
Y		2p25.3	*BC043553*	6	7,047	Dup	(1-5)
Y		2p22.3		14	19,342	Del	(3, 6, 7)
Y	Y	2q37.3	*AK097934; FLJ38379; LOC441309*	23	174,961	Del	
Y	Y	4q13.3	*SLC4A4; NBC*	13	25,713	Del	(2, 6, 7)
Y	Y	5q23.1		36	89,171	Dup	(2, 6-8)
Y		5q33.1	*AK001582*	9	5,428	Del	(7)
Y		5q33.2	*FAM114A2; MFAP3*	15	73,140	Del	
Y	Y	6q14.1		10	42,975	Del	(6, 7)
Y	Y	6q27	*MLLT4; HGC6.3; HGC6.1.1; AK289488; KIF25; FRMD1; FLJ00181*	130	261,473	Dup	(1, 9)
Y	Y	10p12.31		8	6,742	Del	(10)
	Y	11p15.5		26	82,363	Del	(4, 11)
Y	Y	11q11	*OR4C11; OR4P4; OR4S2; OR4C6*	22	70,090	Del	(12)
Y	Y	12p12.1	*IFLTD1*	21	60,485	Del	(1, 13)
Y		12p11.1		8	89,961	Dup	(3)
Y	Y	18q22.1-22.2		10	7,941	Del	
	Y	21q11.2	*C21orf15; DQ579288; DQ579969; C21orf81; BC048201; BC024173; ANKRD20A11P*	18	215,769	Dup	(7)
