# Supplementary material for: Methylomic analysis of monozygotic twins discordant for autism spectrum disorder and related behavioural traits
Source: Mol Psychiatry. 2013 Apr 23;19(4):495–503. doi: 10.1038/mp.2013.41 (PMC3906213; doi:10.1038/mp.2013.41)
Supplement: Supplementary Table 11 [file mp201341x11.doc]

|  |  |  |  |  | ***Including Extreme Twin-pair*** | | ***Excluding Extreme Twin-pair*** | |
| --- | --- | --- | --- | --- | --- | --- | --- | --- |
|  | **Probe Name** | **Gene** | **Chr** | **Position** | ***r*** | **p-value** | ***r*** | **p-value** |
| **Total CAST Score** | cg07753644 | *P2RY11* | 19 | 10083175 | 0.44 | 9.43E-06 | 0.35 | 5.92E-04 |
|  | cg17720231 | *IGSF9* | 1 | 158181998 | -0.43 | 1.67E-05 | -0.32 | 1.65E-03 |
|  | cg09770154 | *MXI1* | 10 | 111957793 | 0.42 | 2.13E-05 | 0.37 | 3.06E-04 |
|  | cg07775675 | *SPHK1* | 17 | 71891517 | -0.42 | 3.11E-05 | -0.34 | 1.02E-03 |
|  | cg19430430 | *COL5A3* | 19 | 9982323 | 0.41 | 3.73E-05 | 0.28 | 7.87E-03 |
|  | cg08909157 | *C9orf66* | 9 | 205561 | 0.41 | 3.74E-05 | 0.41 | 4.88E-05 |
| **Score for Social Autistic Traits** | cg16279786 | *NRXN1* | 2 | 51108810 | -0.41 | 3.45E-05 | -0.28 | 7.25E-03 |
|  | cg21518208 | *KRTHB5* | 12 | 51047683 | 0.41 | 4.27E-05 | 0.37 | 3.06E-04 |
|  | cg10637955 | *BZW1* | 2 | 201384074 | 0.41 | 4.69E-05 | 0.35 | 7.52E-04 |
|  | cg20488657 | *TFF3* | 21 | 42609995 | -0.40 | 7.35E-05 | -0.34 | 8.82E-04 |
|  | cg09770154 | *MXI1* | 10 | 111957793 | 0.39 | 1.13E-04 | 0.33 | 1.43E-03 |
|  | cg09205751 | *NALP6* | 11 | 268394 | -0.39 | 1.22E-04 | -0.37 | 2.64E-04 |
| **Score for Autistic RRBIs** | cg23040064 | *JPH3* | 16 | 86193285 | 0.40 | 6.69E-05 | 0.33 | 1.28E-03 |
|  | cg19626078 | *KNDC1* | 10 | 134823786 | 0.37 | 2.38E-04 | 0.38 | 1.55E-04 |
|  | cg23242898 | *DCC* | 18 | 48121271 | 0.37 | 2.55E-04 | 0.32 | 2.03E-03 |
|  | cg13078388 | *CIRBP* | 19 | 1220048 | -0.37 | 2.72E-04 | -0.32 | 1.93E-03 |
|  | cg23492043 | *ASTN* | 1 | 175400889 | -0.36 | 3.07E-04 | -0.33 | 1.51E-03 |
| **Score for Communicative Autistic Traits** | cg17720231 | *IGSF9* | 1 | 158181998 | -0.47 | 1.75E-06 | -0.39 | 1.03E-04 |
|  | cg08909157 | *C9orf66* | 9 | 205561 | 0.46 | 3.44E-06 | 0.45 | 6.67E-06 |
|  | cg16612699 | *OR8B8* | 11 | 123816428 | 0.45 | 4.31E-06 | 0.42 | 2.43E-05 |
|  | cg00662775 | *TCEAL4* | X | 102727091 | -0.44 | 9.68E-06 | -0.40 | 7.31E-05 |
|  | cg07753644 | *P2RY11* | 19 | 10083175 | 0.43 | 1.63E-05 | 0.35 | 5.25E-04 |
|  | cg09205751 | *NALP6* | 11 | 268394 | -0.43 | 1.72E-05 | -0.41 | 4.09E-05 |
|  | cg26675382 | *NUP43* | 6 | 150109539 | 0.41 | 3.39E-05 | 0.37 | 3.09E-04 |
|  | cg19430430 | *COL5A3* | 19 | 9982323 | 0.41 | 4.82E-05 | 0.30 | 3.38E-03 |
|  | cg07775675 | *SPHK1* | 17 | 71891517 | -0.40 | 5.84E-05 | -0.33 | 1.13E-03 |
