# Supplementary material for: Methylomic analysis of monozygotic twins discordant for autism spectrum disorder and related behavioural traits
Source: Mol Psychiatry. 2013 Apr 23;19(4):495–503. doi: 10.1038/mp.2013.41 (PMC3906213; doi:10.1038/mp.2013.41)
Supplement: Supplementary Table 12 [file mp201341x12.pdf]

ProbeID	Gene	Chromosomal Location	Reported in Analyses	Evidence Type
cg05768141	*KCNJ10*	1q23.2	Twins discordant for communication autistic traits	Candidate gene association (14)
cg01447498	*TSNAX*	1q42.2	ASD-discordant twins	Structural variation (15)
cg16279786	*NRXN1*	2p16.3	Social autistic symptoms score correlation	Structural variation (16-22)
cg23627134	*ARHGAP15*	2q22.2	ASD-discordant twins	Structural variation (23)
cg10562586	*MAP2*	2q34	Sporadic *versus* familial ASD	Gene Expression (24)<br>Structural variation (25)
cg19235307	*MBD4*	3q21.3	ASD-discordant twins	Candidate gene association (26)
cg16970232	*APC*	5p15.33	Case *versus* control	Candidate gene association (27)
cg13234848	*AUTS2*	7q11.22	Sporadic *versus* familial ASD	Gene Expression (28)<br>Structural variation (7, 16, 32, 59, 60)
cg22492966	*JMJD1C*	10q21.2	Discordant twins for RRBIs	Candidate gene association (29)
cg15089487	*THAP10*	15q23	Twins discordant for social autistic traits	Structural variation (2, 30-32)
cg02171545	*SNRPN*	15q11.2	Discordant twins for RRBIs	Candidate gene association (33)<br>Structural variation (2, 3, 6, 7, 30, 32, 34-41)
cg05881762	*UBE3A*	15q11.2	Twins discordant for communication autistic traits	Candidate gene association (42)<br>Gene expression(43)<br>Structural variation (5, 7, 13-16, 18, 33-35, 37-46)
cg15940569	*GABRB3*	15q12	Twins discordant for social autistic traits	Candidate gene association (44-47)<br>Gene expression (48)<br>Structural variation (2, 3, 6, 7, 30, 34, 40, 41)<br>Epigenetics (49)
cg22584138	*SLC6A4*	17q11.2	Case *versus* control	Candidate gene association (50-55)<br>Linkage (45, 52, 56, 57)
cg19837131	*PIK3C3*	18q12.3	Combined discordant group	Linkage (58)<br>Structural variation (32)
cg02345317	*NLGN3*	Xq13.1	Twins discordant for social autistic traits	Candidate gene association (14, 25-27)<br>Gene expression (59)<br>Structural variation (30, 32)
cg27198824	*AFF2*	Xq28	Twins discordant for social autistic traits;<br>Discordant twins for RRBIs;<br>Combined discordant group	Structural variation (35, 60-63)
