# Supplementary figures and images for: Methylomic analysis of monozygotic twins discordant for autism spectrum disorder and related behavioural traits
Source: Mol Psychiatry. 2013 Apr 23;19(4):495–503. doi: 10.1038/mp.2013.41 (PMC3906213; doi:10.1038/mp.2013.41)

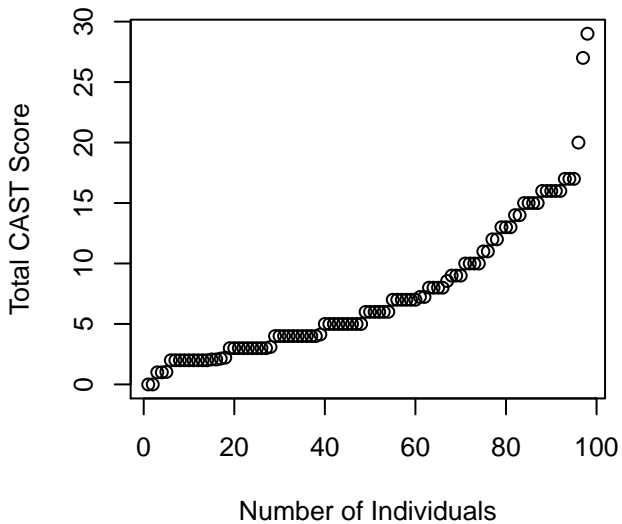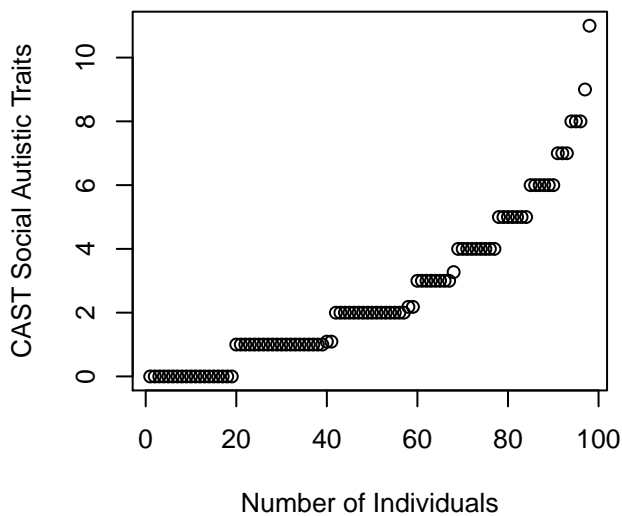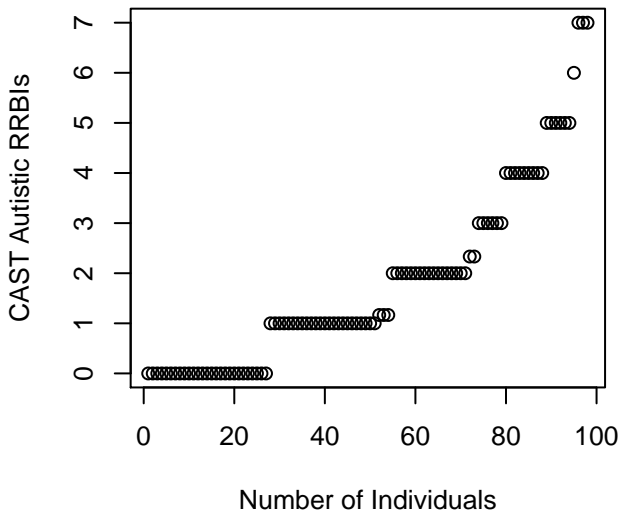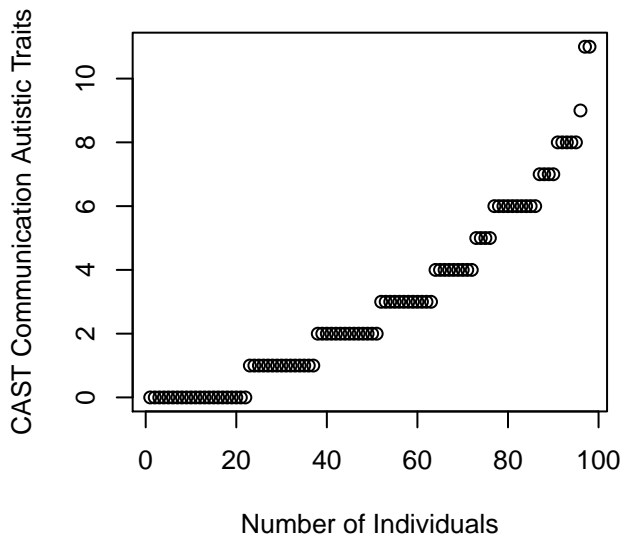

Supplement: Supplementary Figure 1 [file mp201341x13.pdf]

**a**

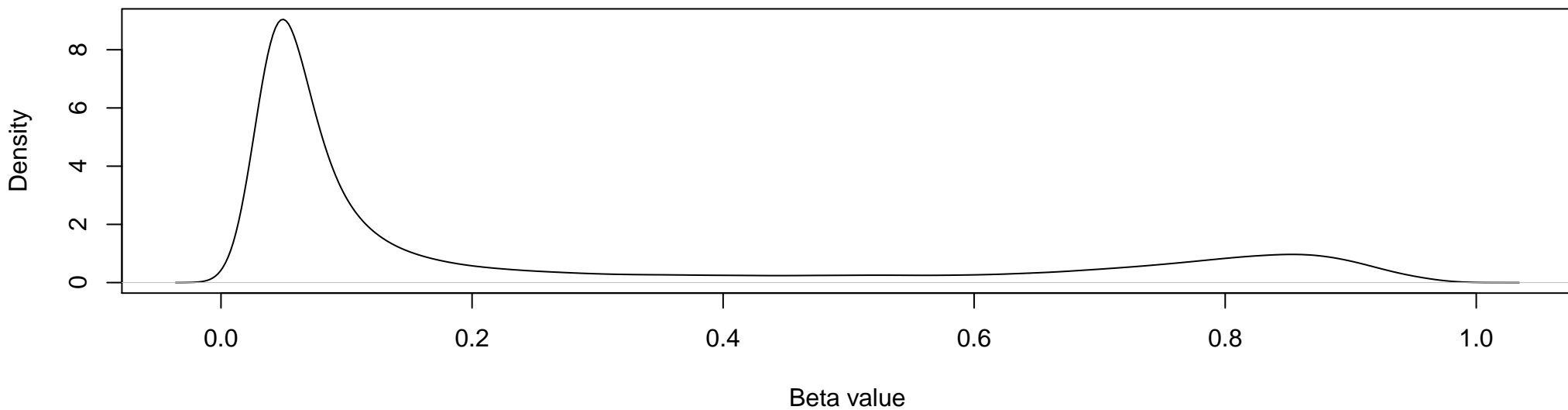

**b**

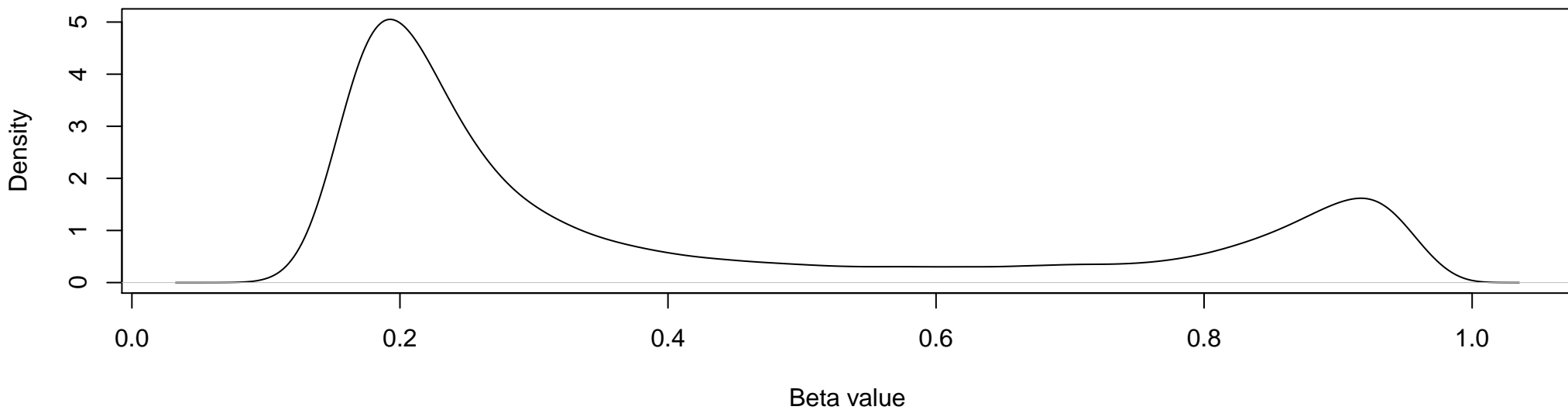

Supplement: Supplementary Figure 2 [file mp201341x14.pdf]

a

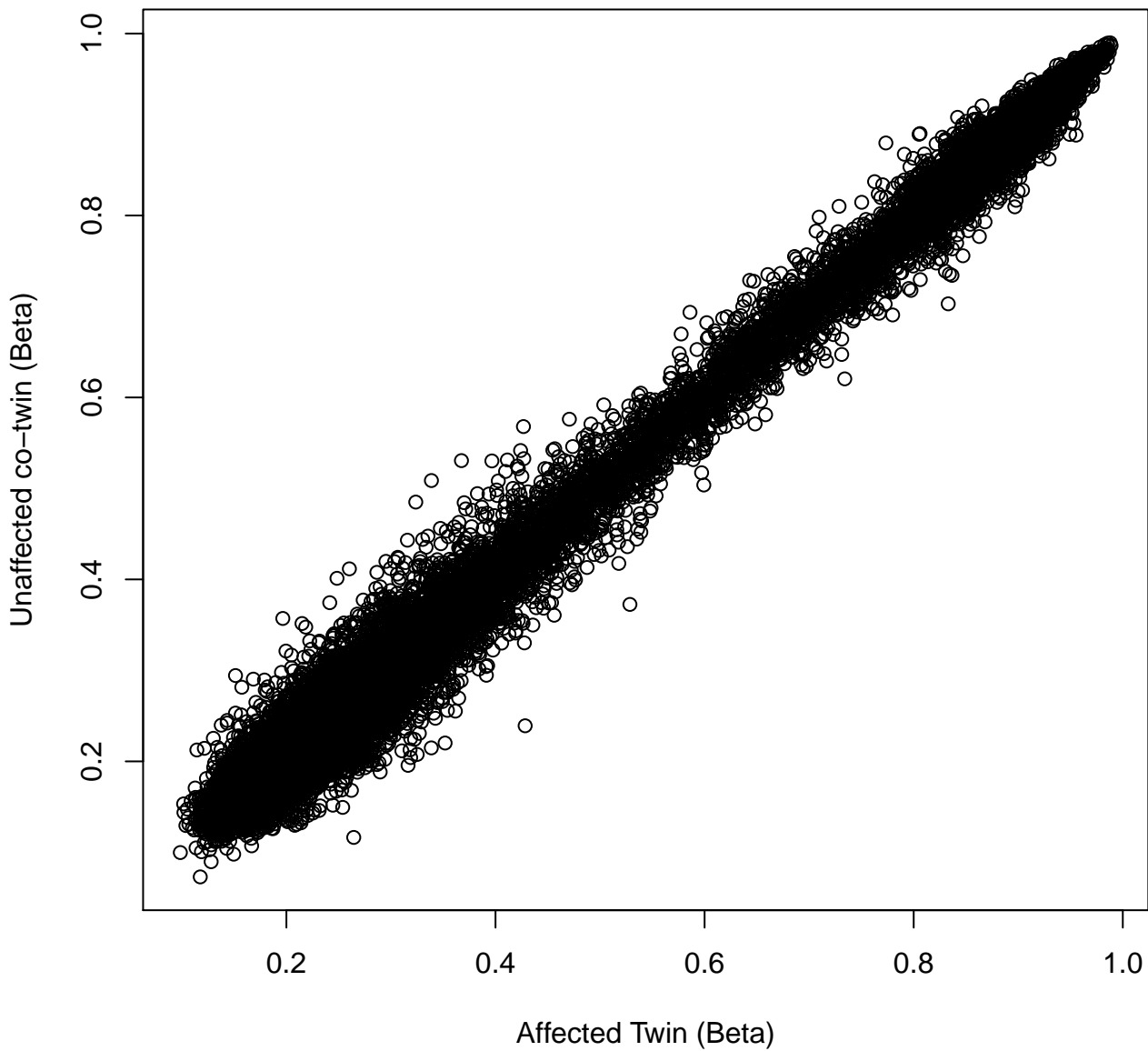

b

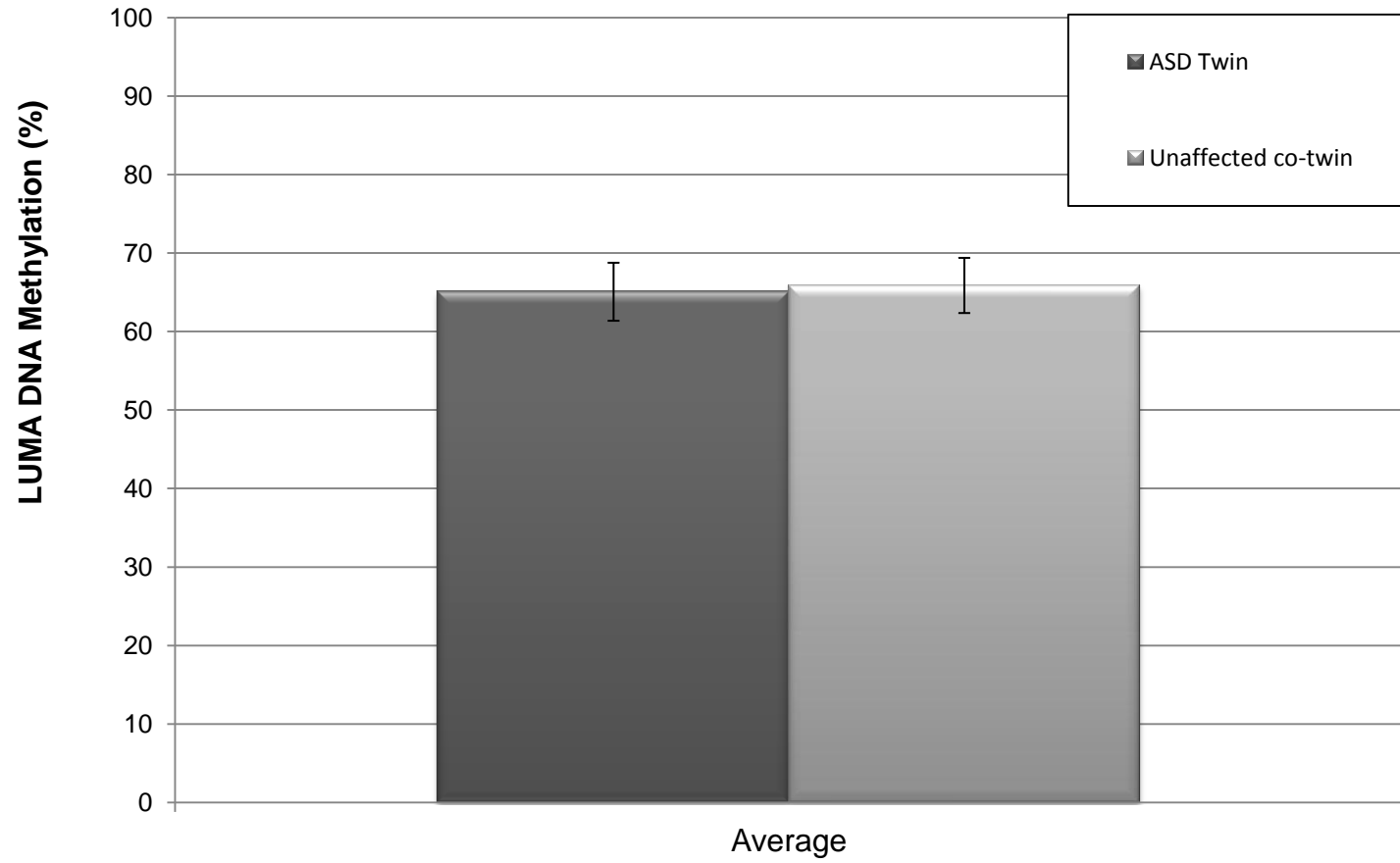

Supplement: Supplementary Figure 4 [file mp201341x16.pdf]

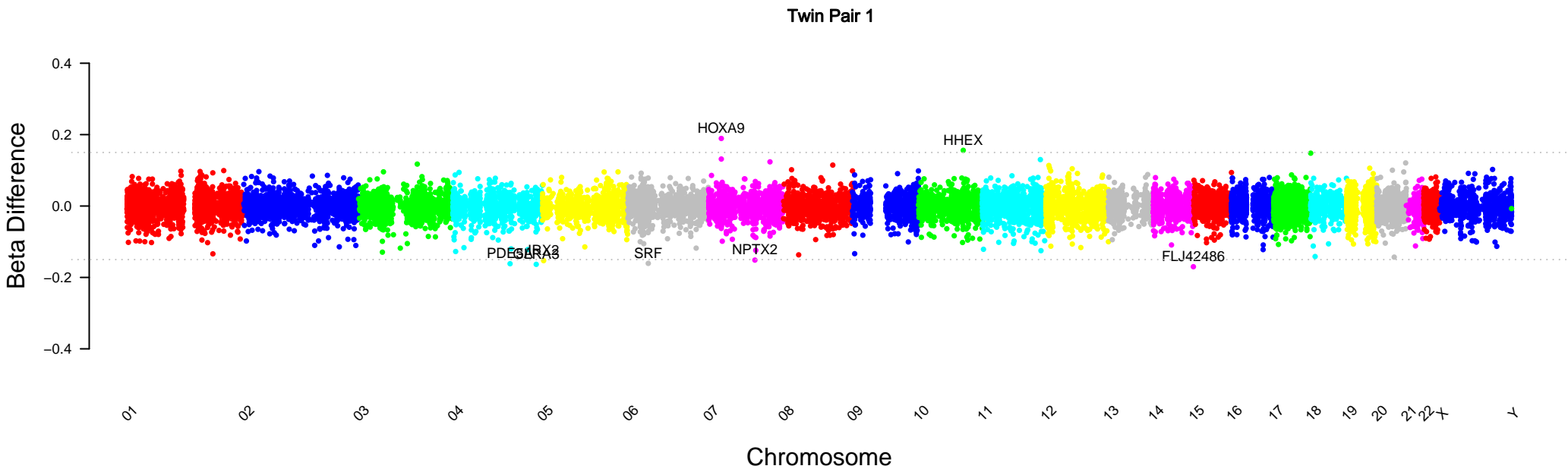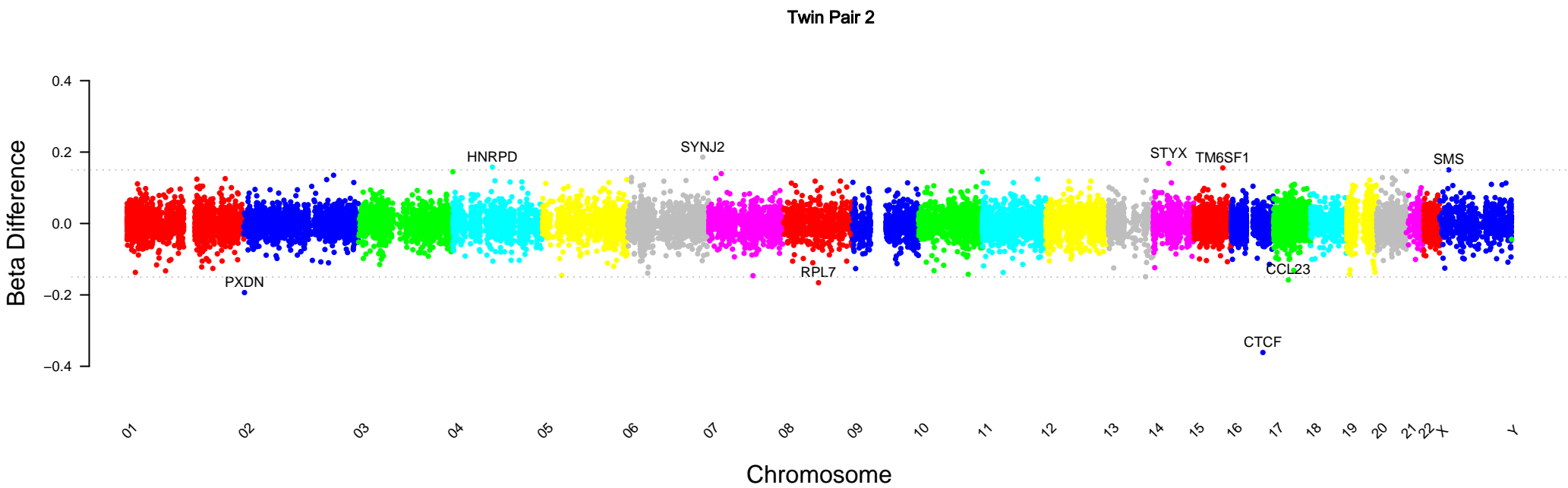

Twin Pair 3

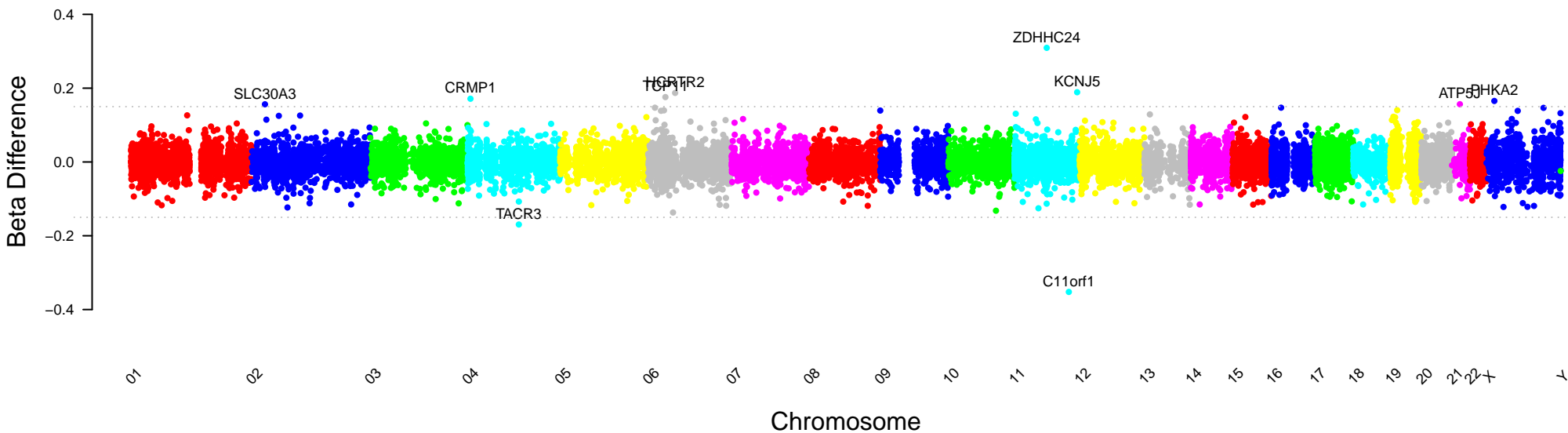

Twin Pair 4

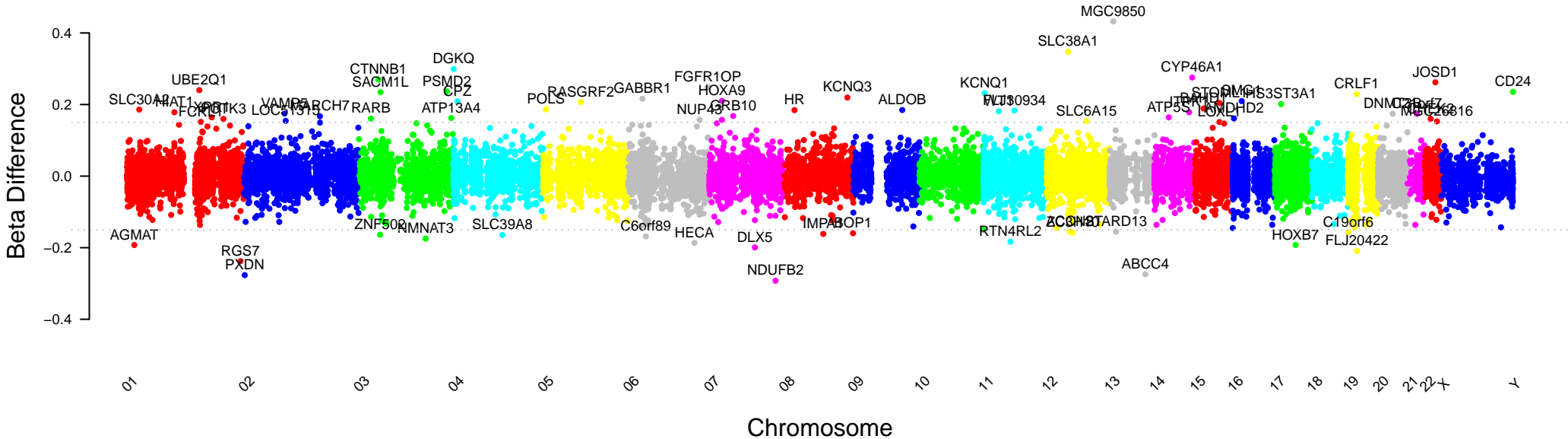

Supplement: Supplementary Figure 5 [file mp201341x17.pdf]

**a****THAP10**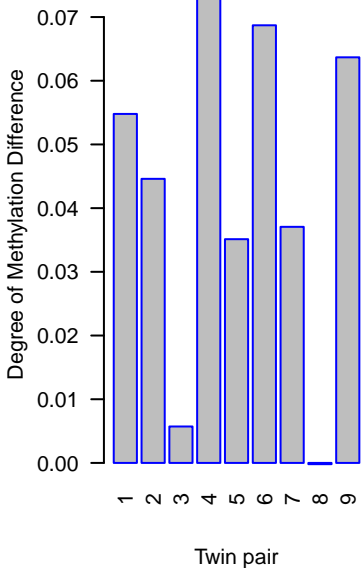**OIP5**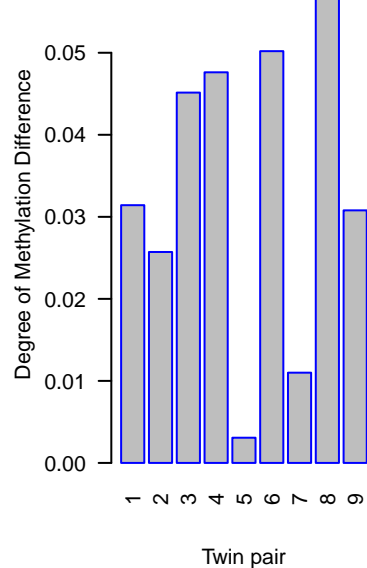**ZNF12**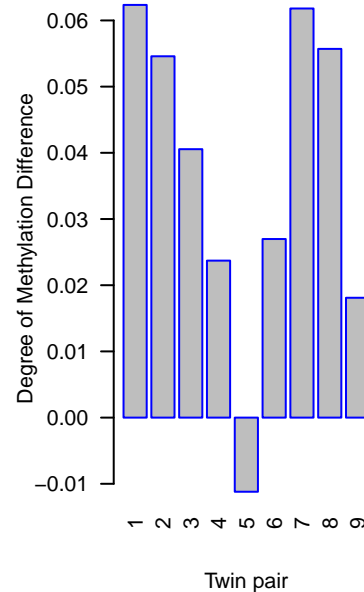**IRAK1**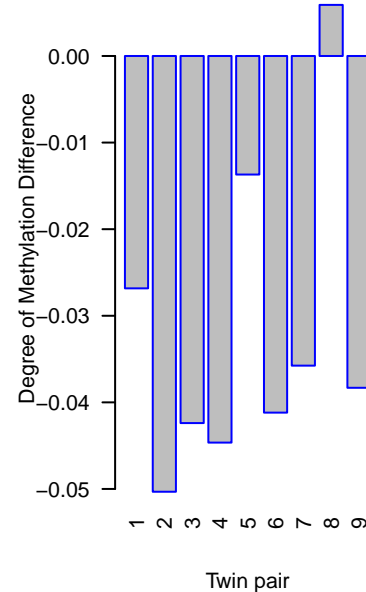**ATP2B4**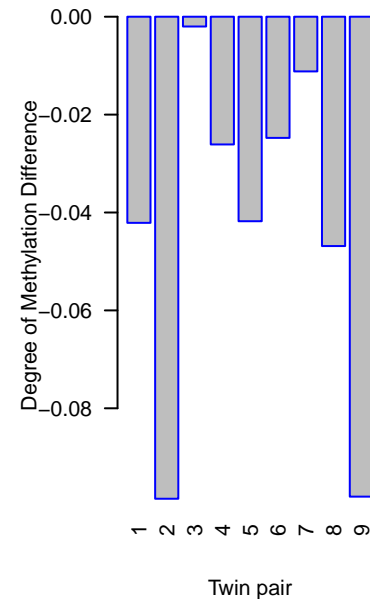**PRDM15**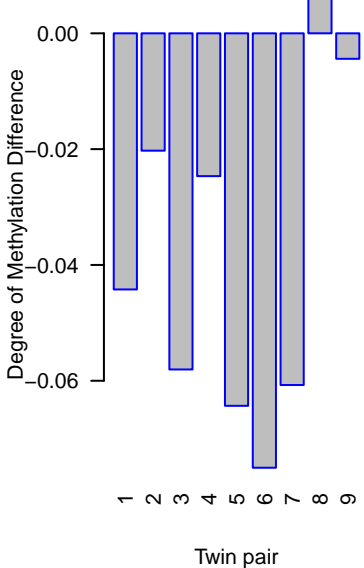**GABRB3**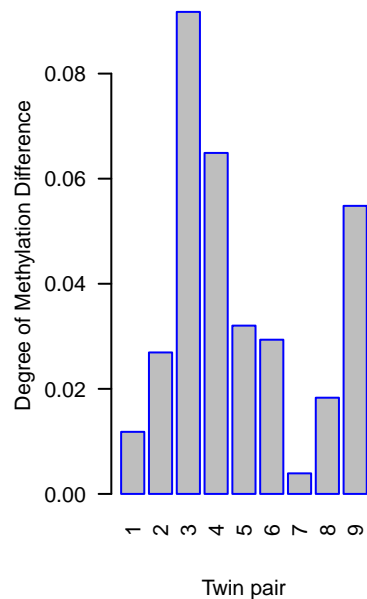**DKFZP686A10121**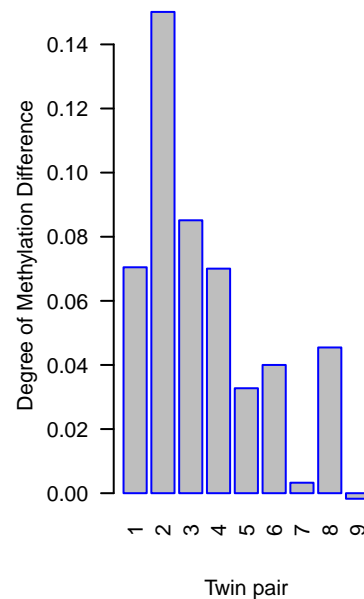**RAB20**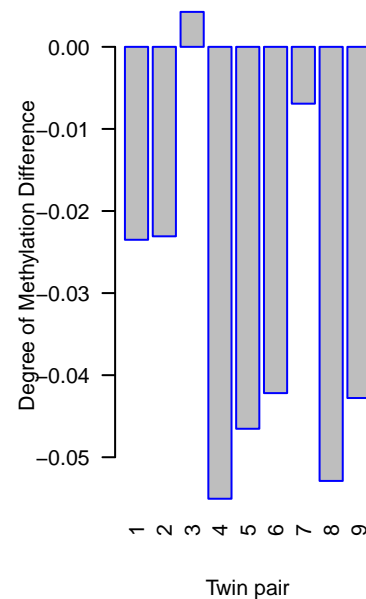**TMEM70**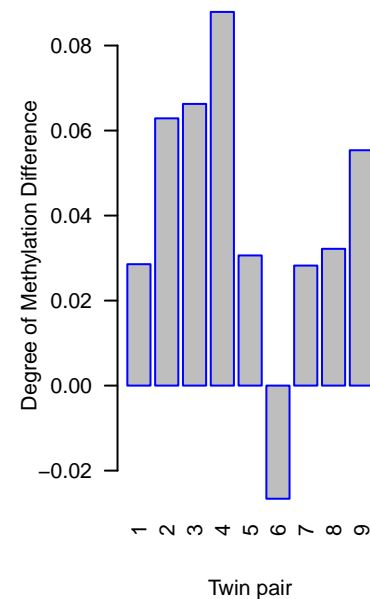

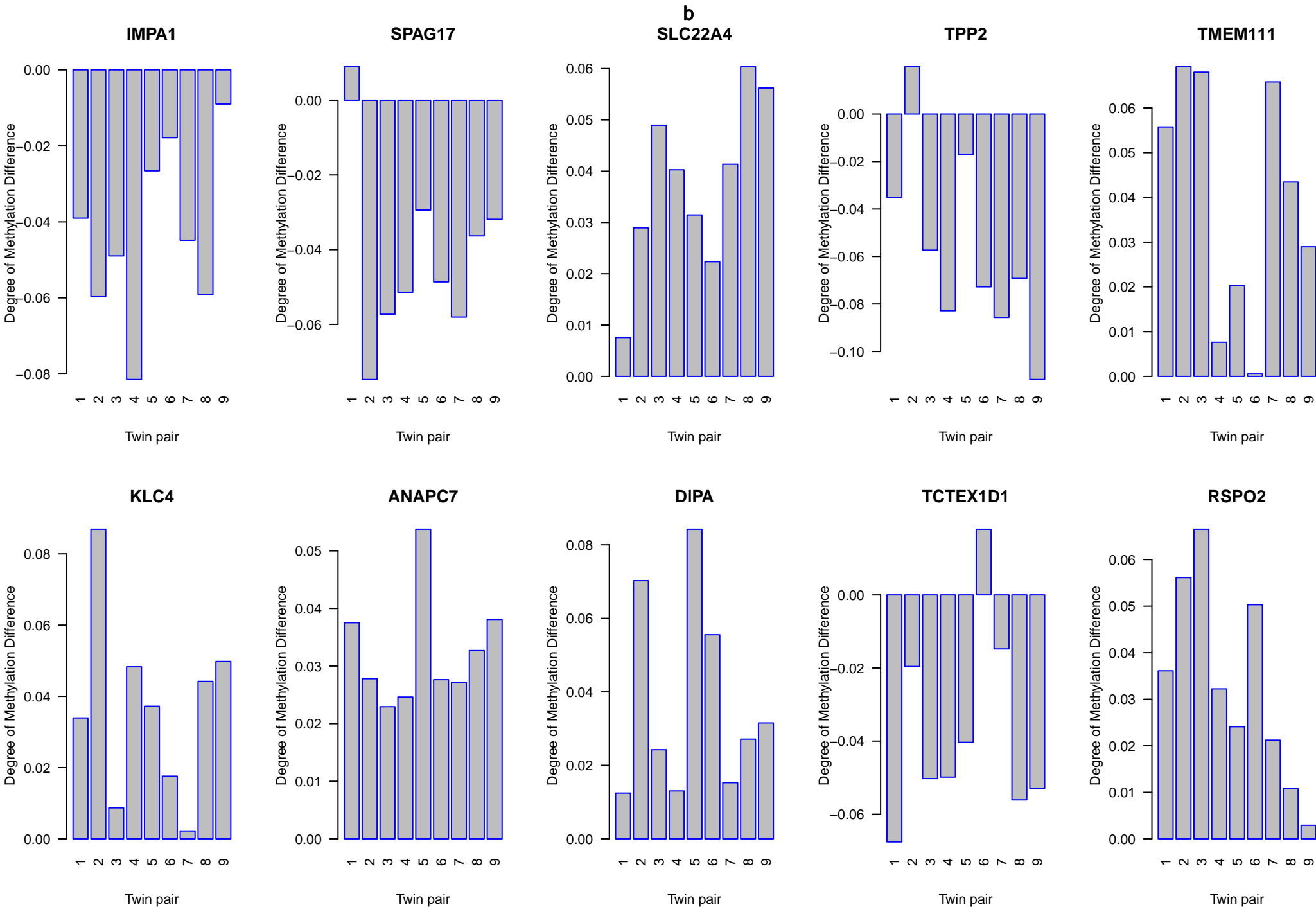

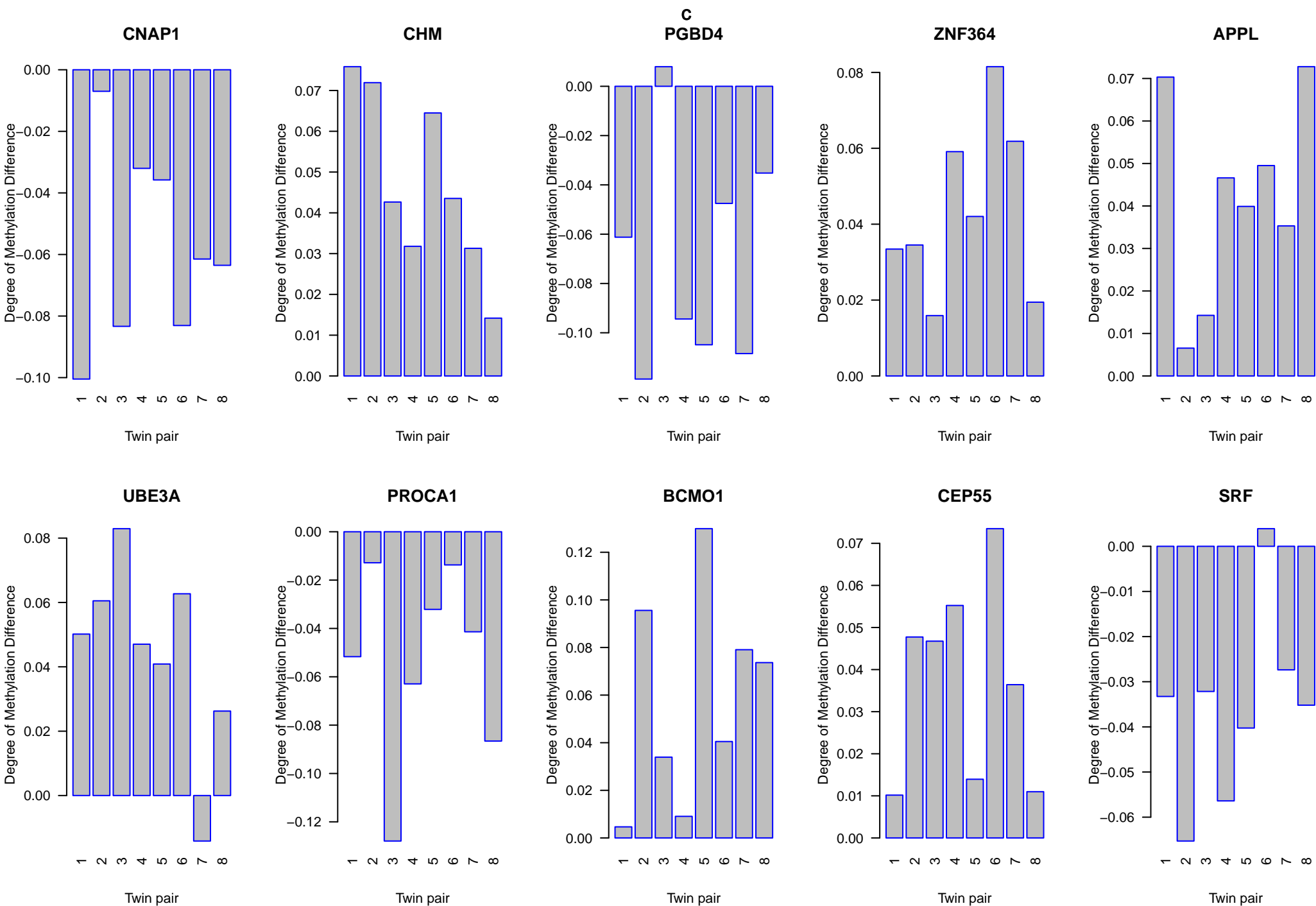

Supplement: Supplementary Figure 6 [file mp201341x18.pdf]

**PIK3C3**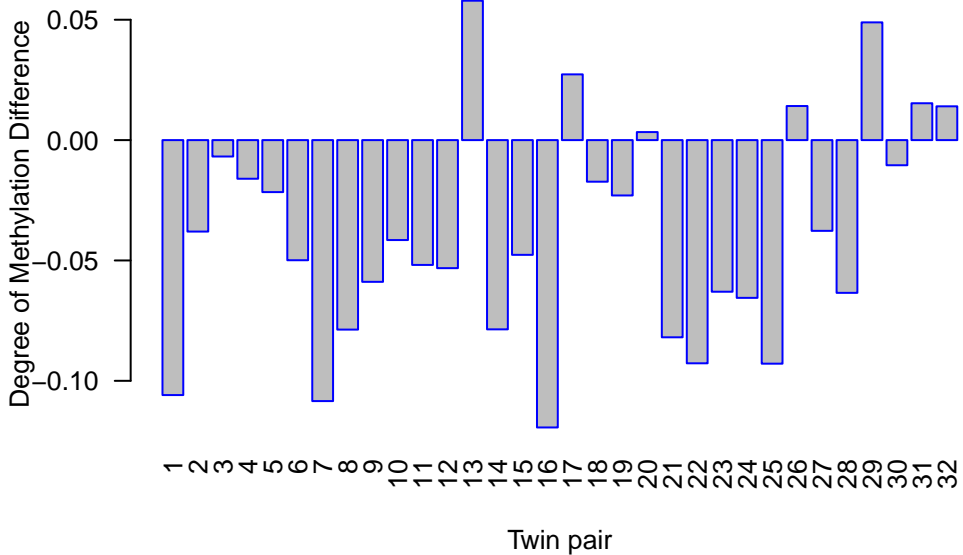**SMEK2**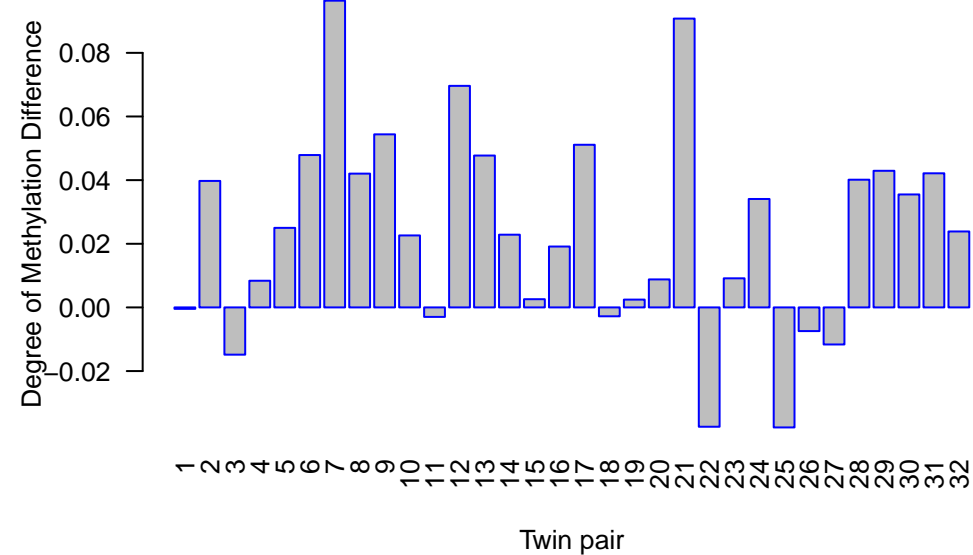**SCO1**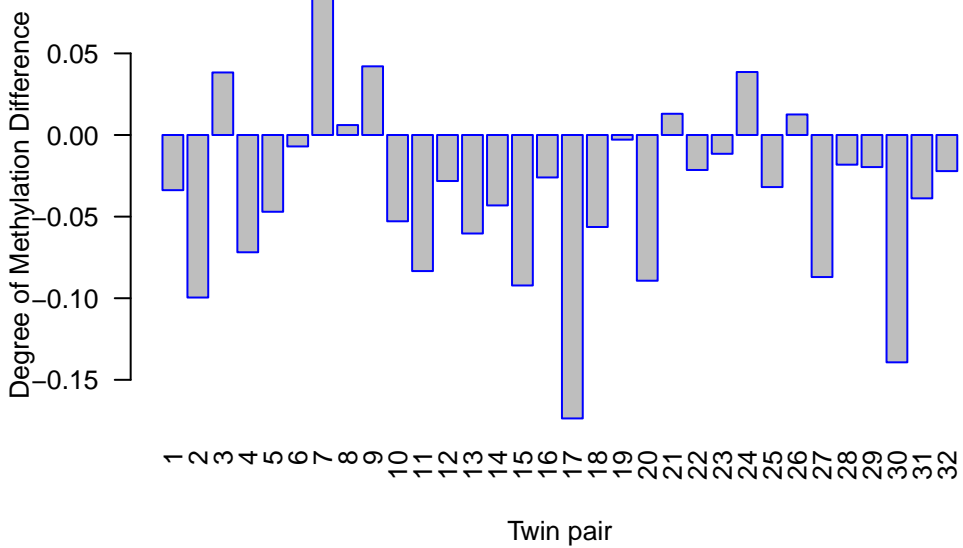**AFF2**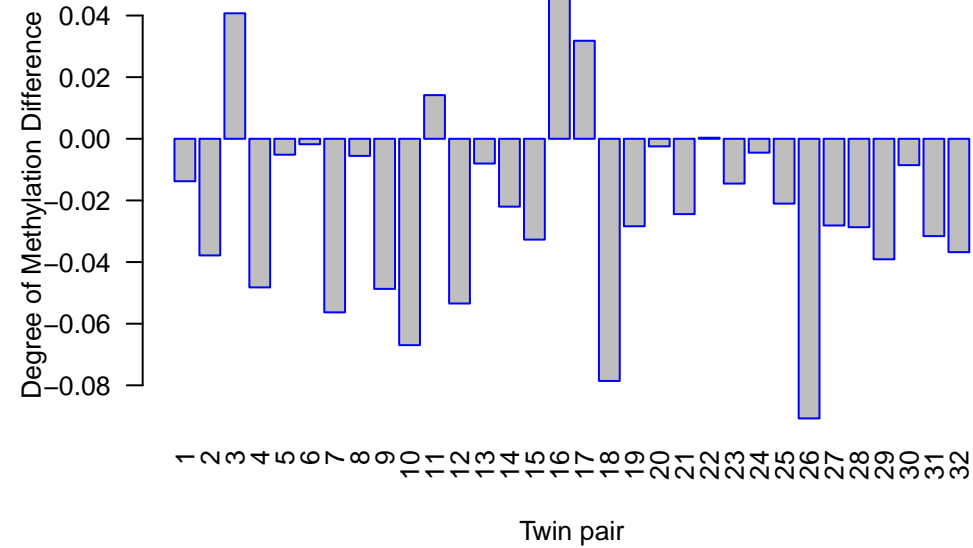

Supplement: Supplementary Figure 7 [file mp201341x19.pdf]

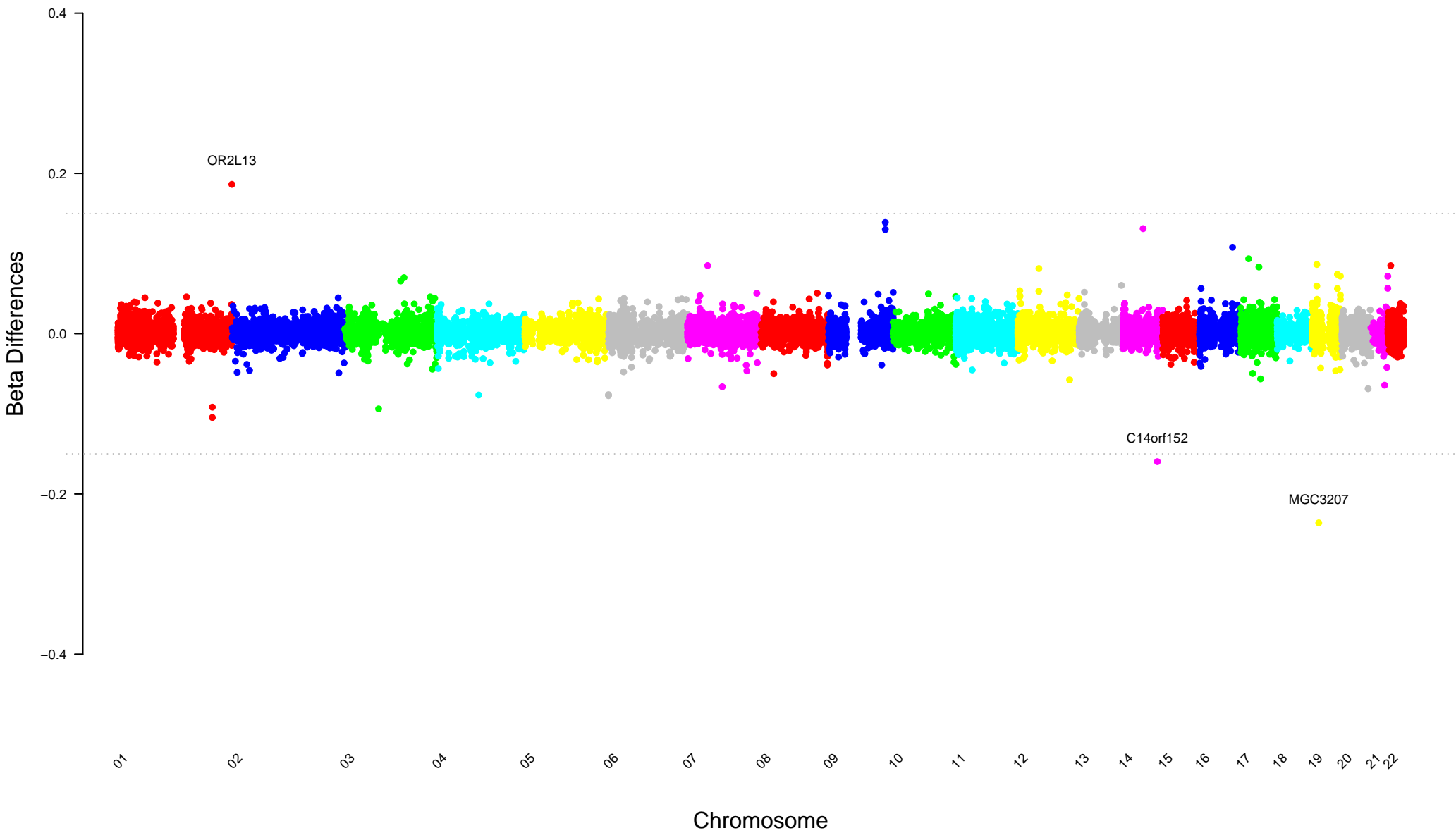

Supplement: Supplementary Figure 8 [file mp201341x20.pdf]

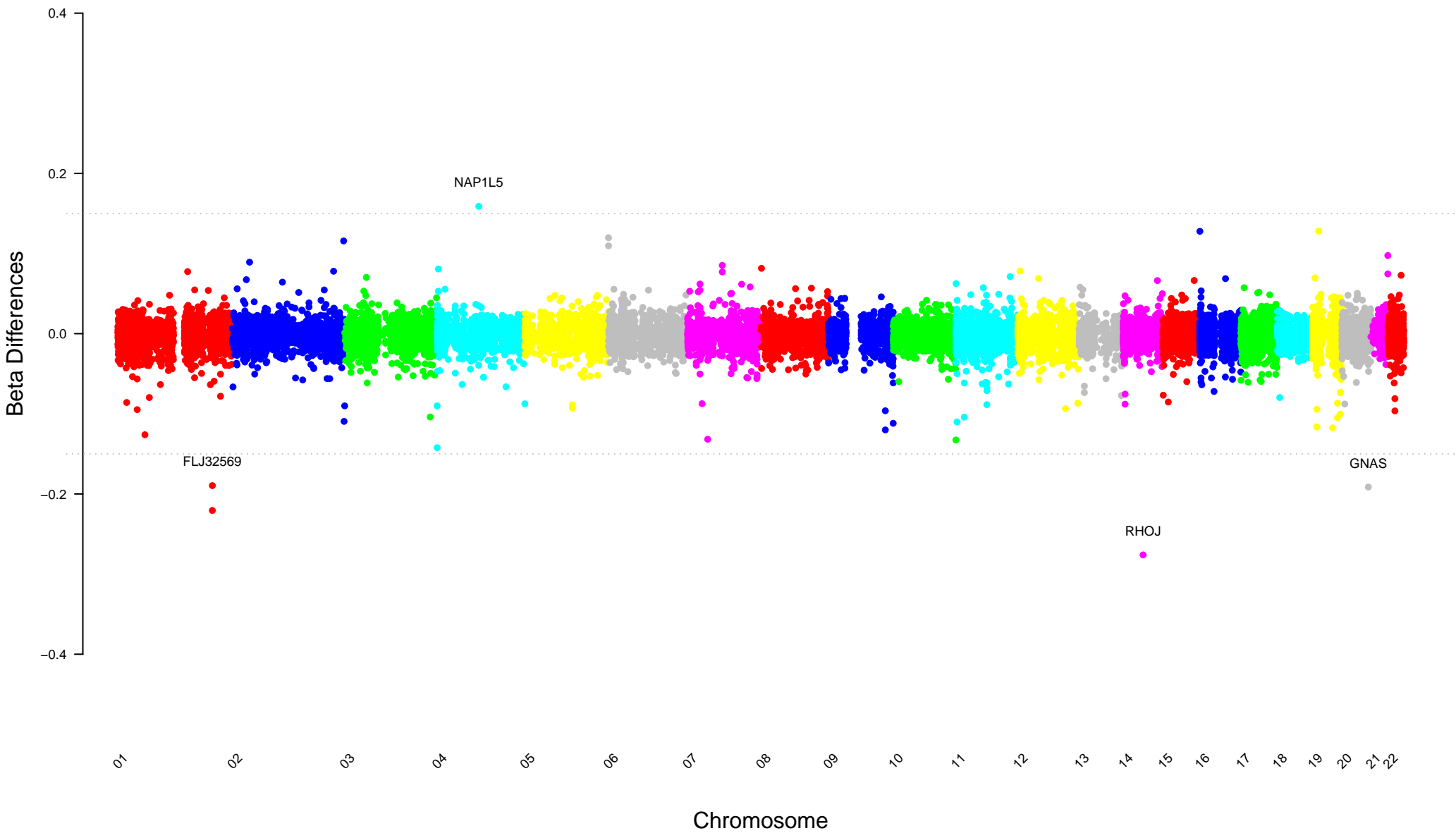

Supplement: Supplementary Figure 9 [file mp201341x21.pdf]

**SCD**

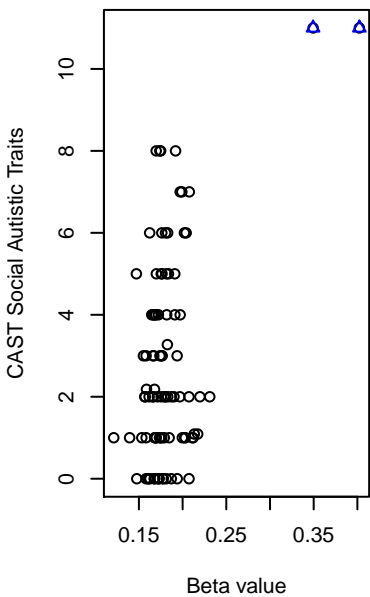

## C9orf72

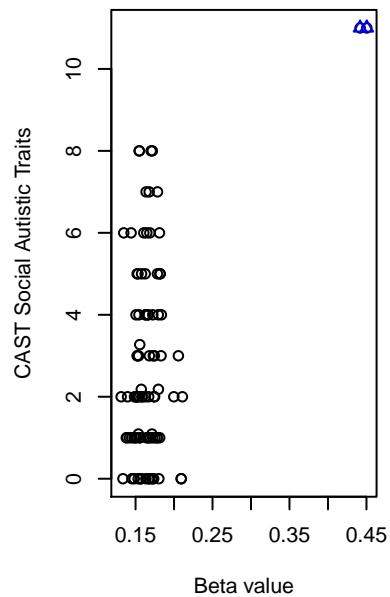

**a**  
**BTK**

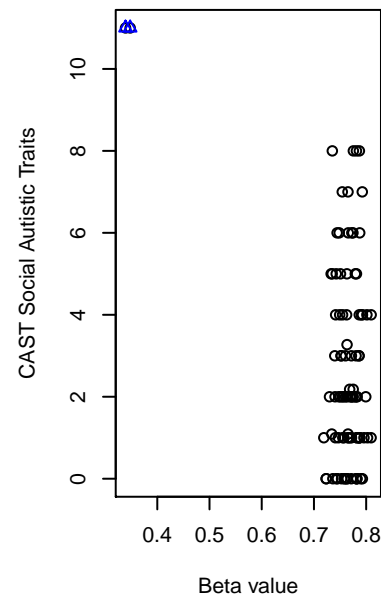

**NRXN1**

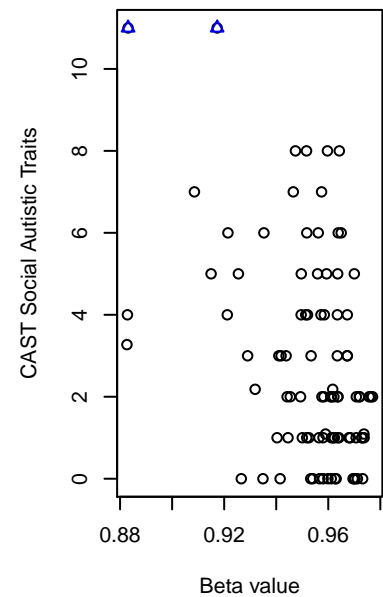

**KRTHB5**

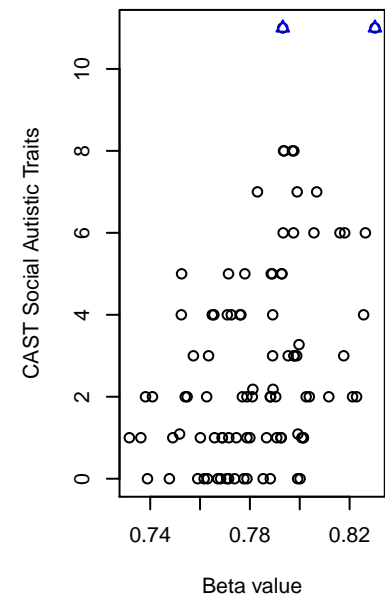

**SCT**

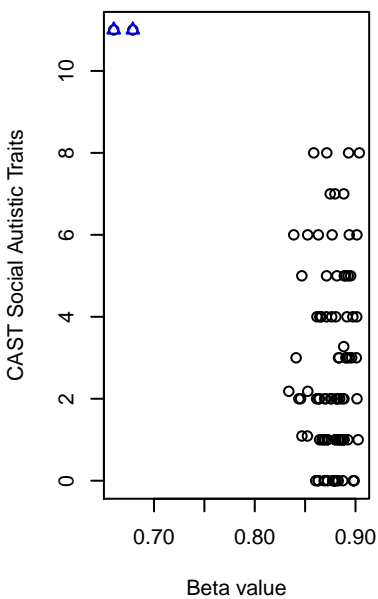

**BZW1**

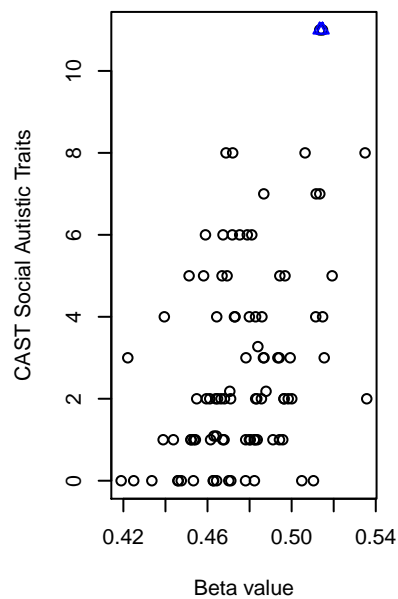

## TFF3

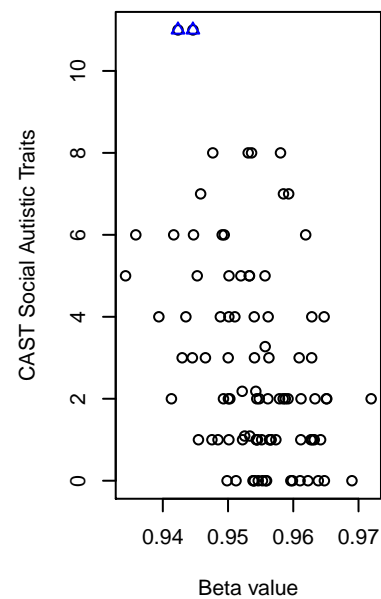

MXI1

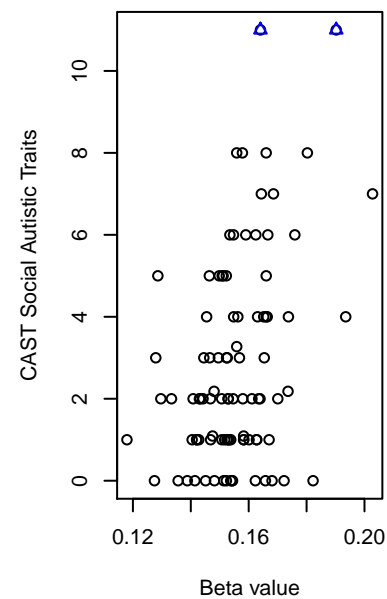

## NALP6

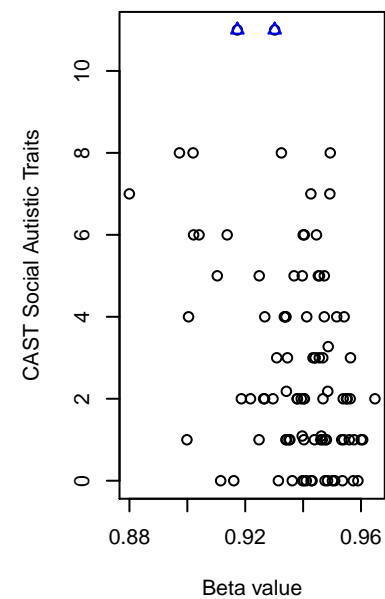

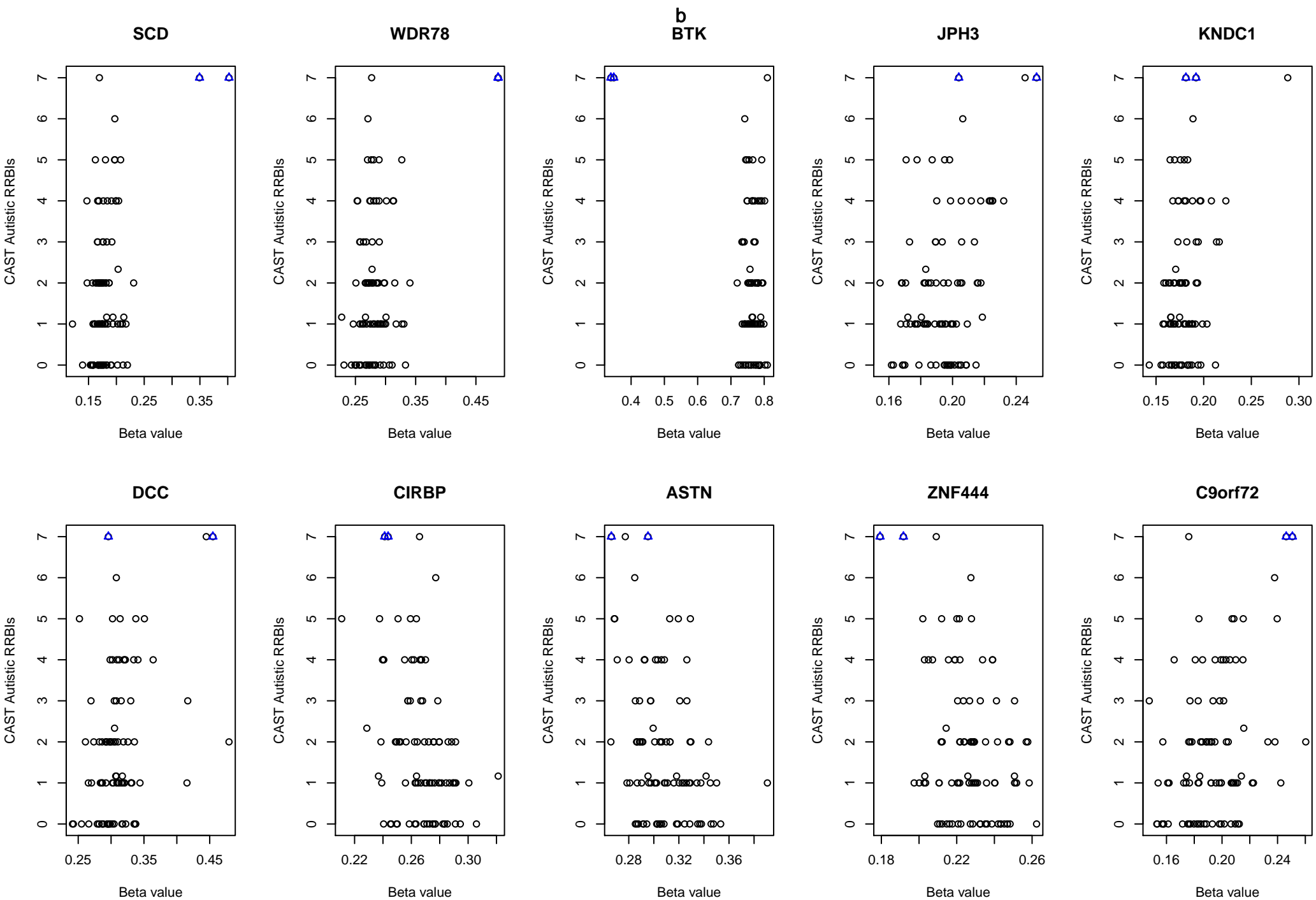

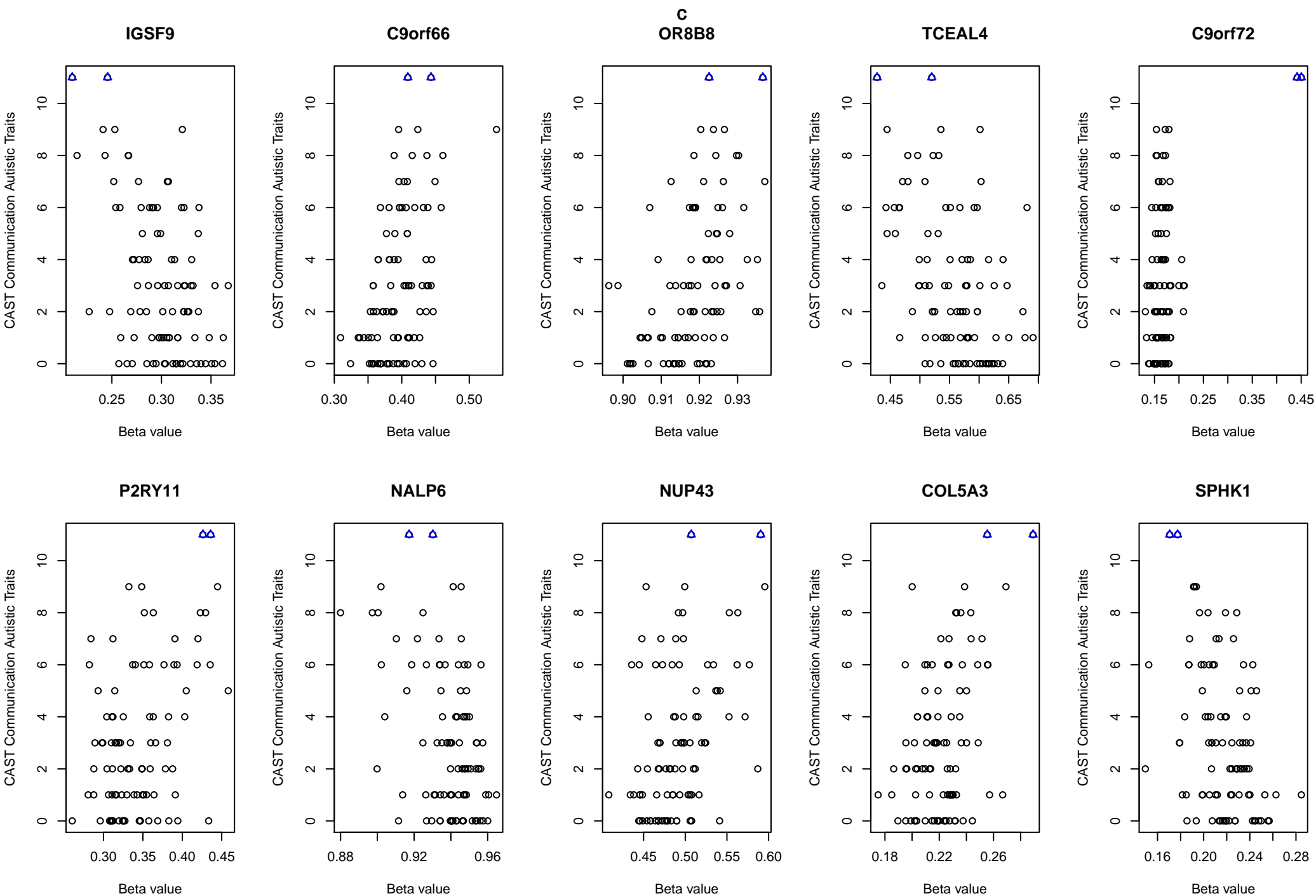

Supplement: Supplementary Figure 10 [file mp201341x22.pdf]

a

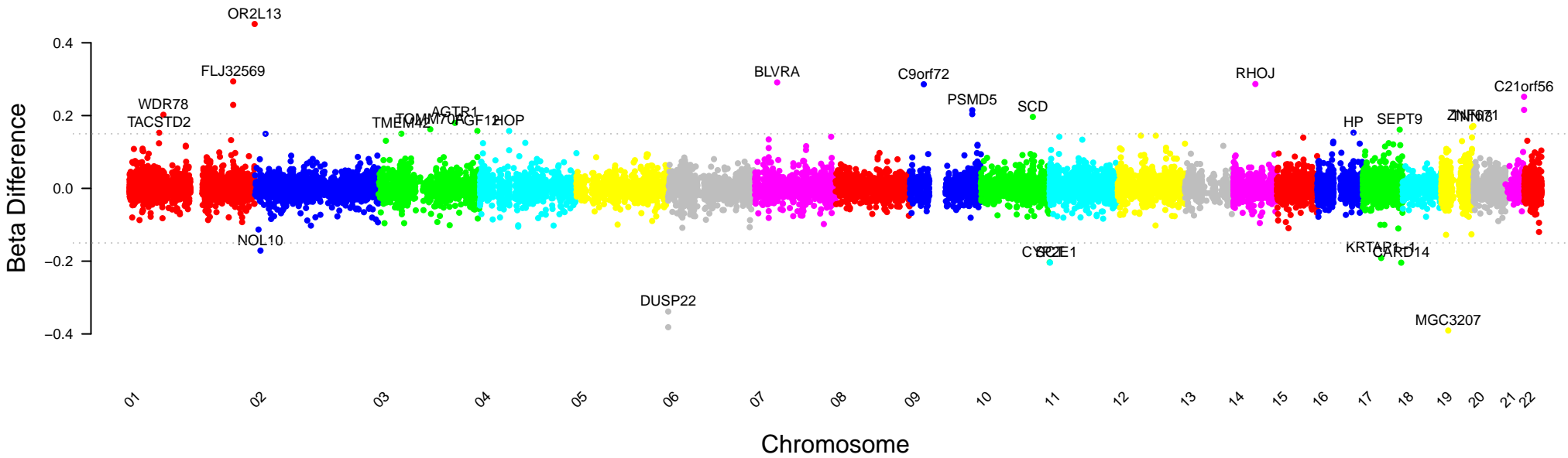

b

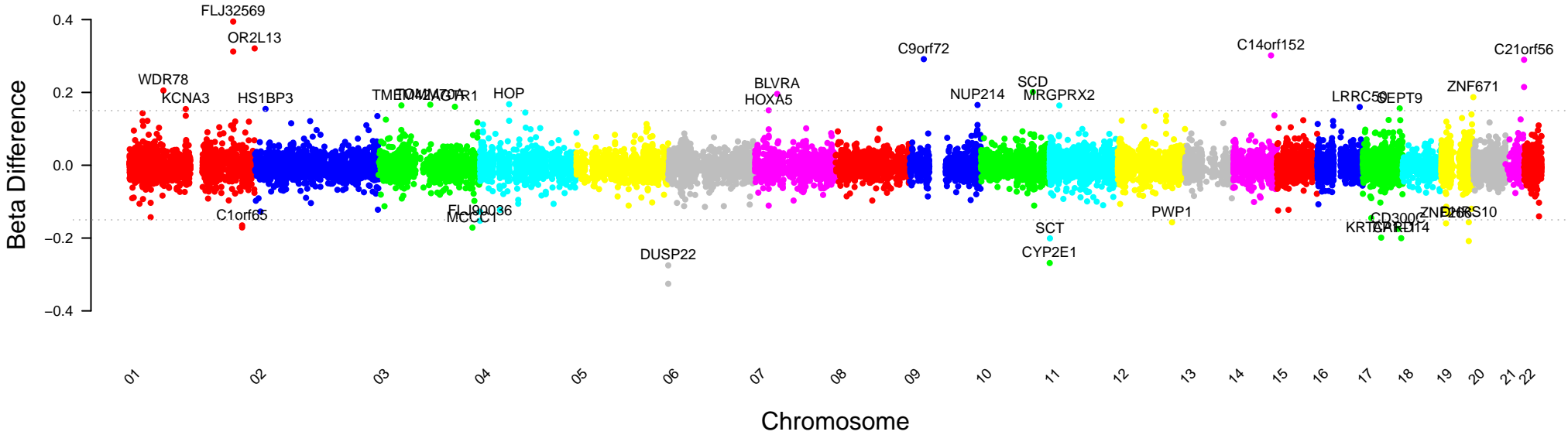

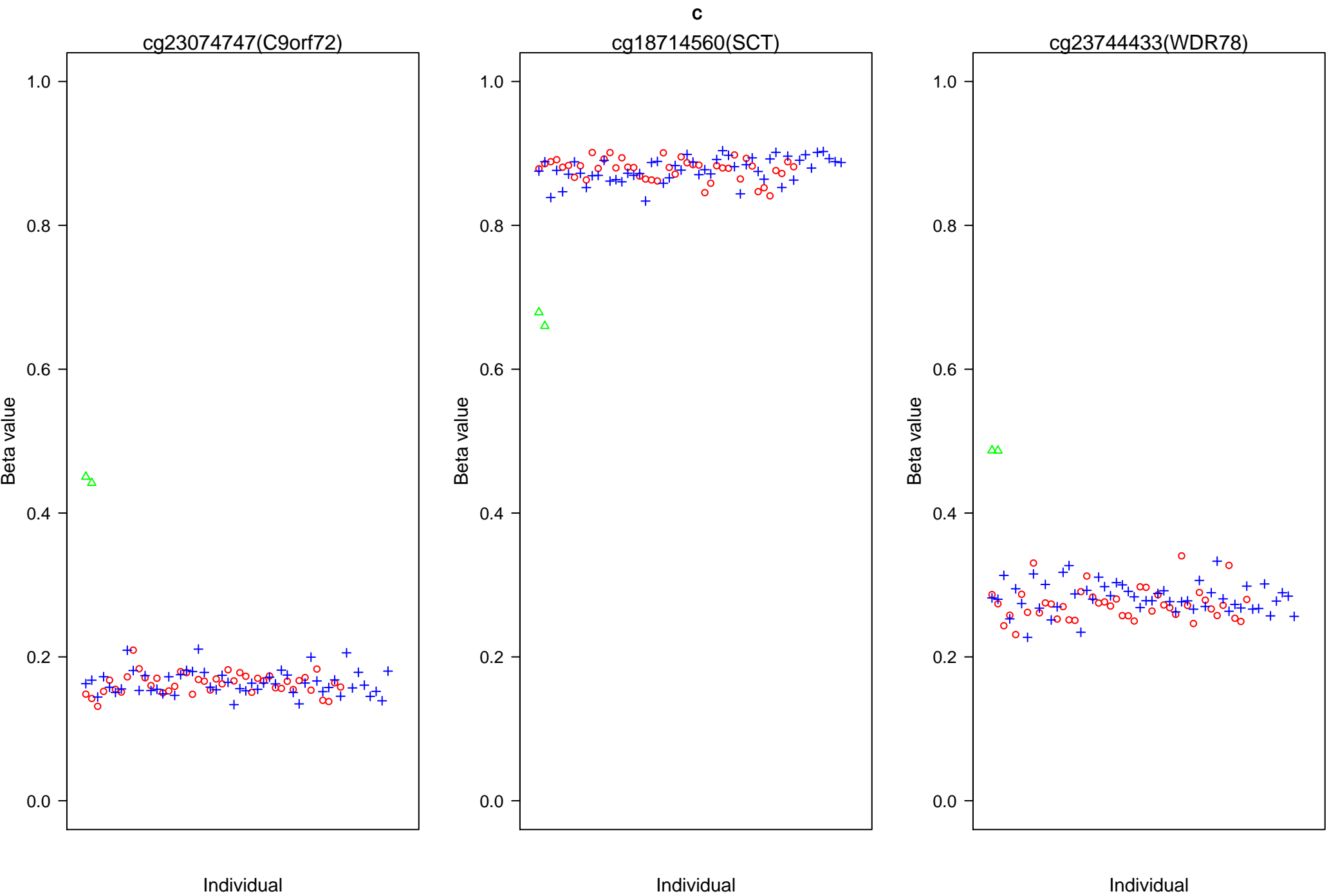

Supplement: Supplementary Figure 11 [file mp201341x23.pdf]
