# Supplementary material for: Methylomic analysis of monozygotic twins discordant for autism spectrum disorder and related behavioural traits
Source: Mol Psychiatry. 2013 Apr 23;19(4):495–503. doi: 10.1038/mp.2013.41 (PMC3906213; doi:10.1038/mp.2013.41)
Supplement: Supplementary Figure 3 [file mp201341x15.pdf]

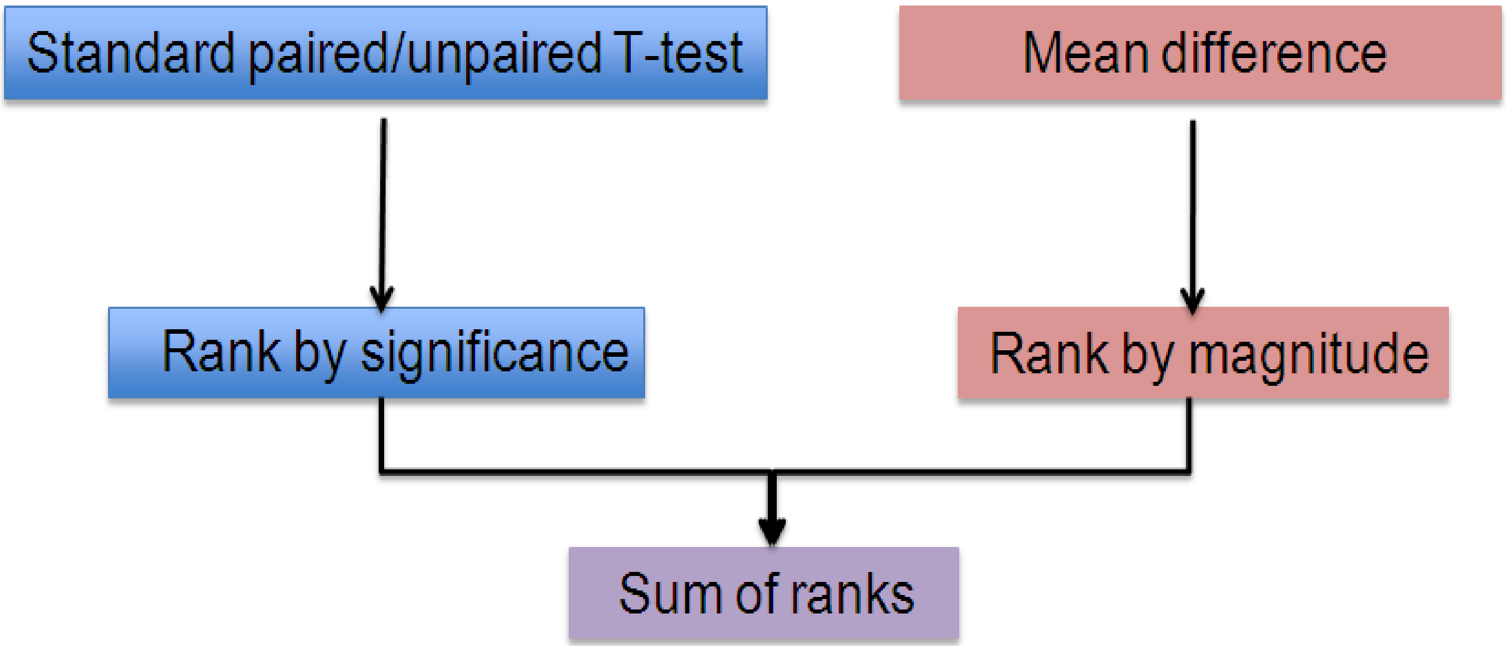

<u>Paired T-test</u>

1) Discordant for ASD
2) Discordant for impairments in social symptoms
3) Discordant for impairments in non-social symptoms
4) Discordant for communication impairments
5) Combined high scoring VS low scoring for ASD traits

<u>Unpaired T-test</u>

1) ASD VS Controls
2) Sporadic VS familial ASD
