# Supplementary material for: Methylomic analysis of monozygotic twins discordant for autism spectrum disorder and related behavioural traits
Source: Mol Psychiatry. 2013 Apr 23;19(4):495–503. doi: 10.1038/mp.2013.41 (PMC3906213; doi:10.1038/mp.2013.41)
Supplement: Supplementary Information [file mp201341x24.pdf]

References for Supplementary Materials

1. Rosenfeld JA, Ballif BC, Torchia BS, Sahoo T, Ravnan JB, Schultz R *et al.* Copy number variations associated with autism spectrum disorders contribute to a spectrum of neurodevelopmental disorders. *Genet Med* 2010; **12**(11)**:** 694-702.

2. Szatmari P, Paterson AD, Zwaigenbaum L, Roberts W, Brian J, Liu XQ *et al.* Mapping autism risk loci using genetic linkage and chromosomal rearrangements. *Nature genetics* 2007; **39**(3)**:** 319-328.

3. Christian SL, Brune CW, Sudi J, Kumar RA, Liu S, Karamohamed S *et al.* Novel submicroscopic chromosomal abnormalities detected in autism spectrum disorder. *Biol Psychiatry* 2008; **63**(12)**:** 1111-1117.

4. Itsara A, Wu H, Smith JD, Nickerson DA, Romieu I, London SJ *et al.* De novo rates and selection of large copy number variation. *Genome research* 2010; **20**(11)**:** 1469-1481.

5. Meyer KJ, Axelsen MS, Sheffield VC, Patil SR, Wassink TH. Germline mosaic transmission of a novel duplication of PXDN and MYT1L to two male half-siblings with autism. *Psychiatric genetics* 2012; **22**(3)**:** 137-140.

6. Marshall CR, Noor A, Vincent JB, Lionel AC, Feuk L, Skaug J *et al.* Structural variation of chromosomes in autism spectrum disorder. *American journal of medical genetics* 2008; **82**(2)**:** 477-488.

7. Pinto D, Pagnamenta AT, Klei L, Anney R, Merico D, Regan R *et al.* Functional impact of global rare copy number variation in autism spectrum disorders. *Nature* 2010; **466**(7304)**:** 368-372.

8. Griswold AJ, Ma D, Cukier HN, Nations LD, Schmidt MA, Chung RH *et al.* Evaluation of copy number variations reveals novel candidate genes in autism spectrum disorder-associated pathways. *Human molecular genetics* 2012; **21**(15)**:** 3513-3523.

9. Gregory S, Connelly J, Towers A, Johnson J, Biscocho D, Markunas C *et al.* Genomic and epigenetic evidence for oxytocin receptor deficiency in autism. *BMC Med* 2009; **7**(1)**:** 62.

10. Salyakina D, Cukier HN, Lee JM, Sacharow S, Nations LD, Ma D *et al.* Copy number variants in extended autism spectrum disorder families reveal candidates potentially involved in autism risk. *PloS one* 2011; **6**(10)**:** e26049.

11. Lesca G, Rudolf G, Labalme A, Hirsch E, Arzimanoglou A, Genton P *et al.* Epileptic encephalopathies of the Landau-Kleffner and continuous spike and waves during slow-wave sleep types: Genomic dissection makes the link with autism. *Epilepsia* 2012; **53**(9)**:** 1526-1538.

12. Girirajan S, Brkanac Z, Coe BP, Baker C, Vives L, Vu TH *et al.* Relative burden of large CNVs on a range of neurodevelopmental phenotypes. *PLoS genetics* 2011; **7**(11)**:** e1002334.

13. Lamb AN, Rosenfeld JA, Neill NJ, Talkowski ME, Blumenthal I, Girirajan S *et al.* Haploinsufficiency of SOX5 at 12p12.1 is associated with developmental delays with prominent language delay, behavior problems, and mild dysmorphic features. *Hum Mutat* 2012; **33**(4)**:** 728-740.

14. Sicca F, Imbrici P, D'Adamo MC, Moro F, Bonatti F, Brovedani P *et al.* Autism with seizures and intellectual disability: Possible causative role of Gain-of-function of the inwardly-rectifying K+ channel Kir4. 1. *Neurobiol Dis* 2011**:** 239-247.

15. Williams JM, Beck TF, Pearson DM, Proud MB, Cheung SW, Scott DA. A 1q42 deletion involving DISC1, DISC2, and TSNAX in an autism spectrum disorder. *Am J Med Genet A* 2009; **149**(8)**:** 1758-1762.

16. Kim HG, Kishikawa S, Higgins AW, Seong IS, Donovan DJ, Shen Y *et al.* Disruption of neurexin 1 associated with autism spectrum disorder. *American journal of medical genetics* 2008; **82**(1)**:** 199-207.

17. Yan J, Noltner K, Feng J, Li W, Schroer R, Skinner C *et al.* Neurexin 1alpha structural variants associated with autism. *Neurosci Lett* 2008; **438**(3)**:** 368-370.

18. Liu Y, Hu Z, Xun G, Peng Y, Lu L, Xu X *et al.* Mutation analysis of the NRXN1 gene in a Chinese autism cohort. *J Psychiatr Res* 2012; **46**(5)**:** 630-634.

19. Gauthier J, Siddiqui TJ, Huashan P, Yokomaku D, Hamdan FF, Champagne N *et al.* Truncating mutations in NRXN2 and NRXN1 in autism spectrum disorders and schizophrenia. *Human genetics* 2011; **130**(4)**:** 563-573.

20. Feng J, Schroer R, Yan J, Song W, Yang C, Bockholt A *et al.* High frequency of neurexin 1beta signal peptide structural variants in patients with autism. *Neurosci Lett* 2006; **409**(1)**:** 10-13.

21. Ching MS, Shen Y, Tan WH, Jeste SS, Morrow EM, Chen X *et al.* Deletions of NRXN1 (neurexin-1) predispose to a wide spectrum of developmental disorders. *Am J Med Genet B Neuropsychiatr Genet* 2010; **153B**(4)**:** 937-947.

22. Wisniowiecka-Kowalnik B, Nesteruk M, Peters SU, Xia Z, Cooper ML, Savage S *et al.* Intragenic rearrangements in NRXN1 in three families with autism spectrum disorder, developmental delay, and speech delay. *Am J Med Genet B Neuropsychiatr Genet* 2010; **153B**(5)**:** 983-993.

23. O'Roak BJ, Deriziotis P, Lee C, Vives L, Schwartz JJ, Girirajan S *et al.* Exome sequencing in sporadic autism spectrum disorders identifies severe de novo mutations. *Nature genetics* 2011; **43**(6)**:** 585-589.

24. Mukaetova-Ladinska E, Arnold H, Jaros E, Perry R, Perry E. Depletion of MAP2 expression and laminar cytoarchitectonic changes in dorsolateral prefrontal cortex in adult autistic individuals. *Neuropathol Appl Neurobiol* 2004; **30**(6)**:** 615-623.

25. Pescucci C, Meloni I, Bruttini M, Ariani F, Longo I, Mari F *et al.* Chromosome 2 deletion encompassing the MAP2 gene in a patient with autism and Rett-like features. *Clinical genetics* 2003; **64**(6)**:** 497-501.

26. Cukier HN, Rabionet R, Konidari I, Rayner-Evans MY, Baltos ML, Wright HH *et al.* Novel variants identified in methyl-CpG-binding domain genes in autistic individuals. *Neurogenetics* 2010**; 11**(3):291-303.

27. Zhou XL, Giacobini M, Anderlid BM, Anckarsater H, Omrani D, Gillberg C *et al.* Association of adenomatous polyposis coli (APC) gene polymorphisms with autism spectrum disorder (ASD). *Am J Med Genet B Neuropsychiatr Genet* 2007; **144B**(3)**:** 351-354.

28. Ben-David E, Granot-Hershkovitz E, Monderer-Rothkoff G, Lerer E, Levi S, Yaari M *et al.* Identification of a functional rare variant in autism using genome-wide screen for monoallelic expression. *Human molecular genetics* 2011**; 20**(18): 3632-3641.

29. Castermans D, Vermeesch JR, Fryns JP, Steyaert JG, Van de Ven WJM, Creemers JWM *et al.* Identification and characterization of the TRIP8 and REEP3 genes on chromosome 10q21. 3 as novel candidate genes for autism. *Eur J Hum Genet* 2007; **15**(4)**:** 422-431.

30. Sanders SJ. Multiple Recurrent De Novo CNVs, Including Duplications of the 7q11. 23 Williams Syndrome Region, Are Strongly Associated with Autism. *Neuron* 2011; **70**(5)**:** 863-885.

31. Smith M, Filipek PA, Wu C, Bocian M, Hakim S, Modahl C *et al.* Analysis of a 1 megabase deletion in 15q22 q23 in an autistic patient: Identification of candidate genes for autism and of homologous DNA segments in 15q22 q23 and 15q11 q13. *American journal of medical genetics* 2000; **96**(6)**:** 765-770.

32. Levy D, Ronemus M, Yamrom B, Lee Y, Leotta A, Kendall J *et al.* Rare De Novo and Transmitted Copy-Number Variation in Autistic Spectrum Disorders. *Neuron* 2011; **70**(5)**:** 886-897.

33. Kato C, Tochigi M, Ohashi J, Koishi S, Kawakubo Y, Yamamoto K *et al.* Association study of the 15q11-q13 maternal expression domain in Japanese autistic patients. *Am J Med Genet B Neuropsychiatr Genet* 2008; **147B**(7)**:** 1008-1012.

34. Sebat J, Lakshmi B, Malhotra D, Troge J, Lese-Martin C, Walsh T *et al.* Strong association of de novo copy number mutations with autism. *Science* 2007; **316**(5823)**:** 445-449.

35. Nord AS, Roeb W, Dickel DE, Walsh T, Kusenda M, O'Connor KL *et al.* Reduced transcript expression of genes affected by inherited and de novo CNVs in autism. *Eur J Hum Genet* 2011; **19**(6)**:** 727-731.

36. Keller K, Williams C, Wharton P, Paulk M, Bent Williams A, Gray B *et al.* Routine cytogenetic and FISH studies for 17p11/15q11 duplications and subtelomeric rearrangement studies in children with autism spectrum disorders. *Am J Med Genet A* 2003; **117**(2)**:** 105-111.

37. Talkowski ME, Rosenfeld JA, Blumenthal I, Pillalamarri V, Chiang C, Heilbut A *et al.* Sequencing chromosomal abnormalities reveals neurodevelopmental loci that confer risk across diagnostic boundaries. *Cell* 2012; **149**(3)**:** 525-537.

38. Wassink TH, Piven J, Patil SR. Chromosomal abnormalities in a clinic sample of individuals with autistic disorder. *Psychiatric genetics* 2001; **11**(2)**:** 57-63.

39. Wolpert CM, Menold MM, Bass MP, Qumsiyeh MB, Donnelly SL, Ravan SA *et al.* Three probands with autistic disorder and isodicentric chromosome 15. *American journal of medical genetics* 2000; **96**(3)**:** 365-372.

40. Bremer A, Giacobini M, Nordenskjold M, Brondum-Nielsen K, Mansouri M, Dahl N *et al.* Screening for copy number alterations in loci associated with autism spectrum disorders by two-color multiplex ligation-dependent probe amplification. *Am J Med Genet B Neuropsychiatr Genet* 2010; **153B**(1)**:** 280-285.

41. Sahoo T, Shaw CA, Young AS, Whitehouse NL, Schroer RJ, Stevenson RE *et al.* Array based comparative genomic hybridization analysis of recurrent chromosome 15q rearrangements. *Am J Med Genet A* 2005; **139**(2)**:** 106-113.

42. Guffanti G, Lievers LS, Bonati MT, Marchi M, Geronazzo L, Nardocci N *et al.* Role of UBE3A and ATP10A genes in autism susceptibility region 15q11-q13 in an Italian population: A positive replication for UBE3A. *Psychiatry Res* 2011; **185**(1-2)**:** 33-38.

43. Nishimura Y, Martin CL, Vazquez-Lopez A, Spence SJ, Alvarez-Retuerto AI, Sigman M *et al.* Genome-wide expression profiling of lymphoblastoid cell lines distinguishes different forms of autism and reveals shared pathways. *Human molecular genetics* 2007; **16**(14)**:** 1682-1698.

44. Buxbaum J, Silverman J, Smith C, Greenberg D, Kilifarski M, Reichert J *et al.* Association between a GABRB3 polymorphism and autism. *Molecular psychiatry* 2002; **7**(3)**:** 311-316.

45. McCauley JL, Olson LM, Delahanty R, Amin T, Nurmi EL, Organ EL *et al.* A linkage disequilibrium map of the 1-Mb 15q12 GABA(A) receptor subunit cluster and association to autism. *Am J Med Genet B Neuropsychiatr Genet* 2004; **131B**(1)**:** 51-59.

46. Delahanty RJ, Kang JQ, Brune CW, Kistner EO, Courchesne E, Cox NJ *et al.* Maternal transmission of a rare GABRB3 signal peptide variant is associated with autism. *Molecular psychiatry* 2011; **16**(1)**:** 86-96.

47. Jiao Y, Chen R, Ke X, Cheng L, Chu K, Lu Z *et al.* Single nucleotide polymorphisms predict symptom severity of autism spectrum disorder. *J Autism Dev Disord* 2012; **42**(6)**:** 971-983.

48. Voineagu I, Wang X, Johnston P, Lowe JK, Tian Y, Horvath S *et al.* Transcriptomic analysis of autistic brain reveals convergent molecular pathology. *Nature* 2011; **474**(7351)**:** 380-384.

49. Hogart A, Nagarajan RP, Patzel KA, Yasui DH, Lasalle JM. 15q11-13 GABAA receptor genes are normally biallelically expressed in brain yet are subject to epigenetic dysregulation in autism-spectrum disorders. *Human molecular genetics* 2007; **16**(6)**:** 691-703.

50. Kim SJ, Cox N, Courchesne R, Lord C, Corsello C, Akshoomoff N *et al.* Transmission disequilibrium mapping at the serotonin transporter gene (SLC6A4) region in autistic disorder. *Molecular psychiatry* 2002; **7**(3)**:** 278-288.

51. McCauley J, Olson L, Dowd M, Amin T, Steele A, Blakely R *et al.* Linkage and association analysis at the serotonin transporter (SLC6A4) locus in a rigid compulsive subset of autism. *Am J Med Genet B Neuropsychiatr Genet* 2004; **127**(1)**:** 104-112.

52. Sutcliffe JS, Delahanty RJ, Prasad HC, McCauley JL, Han Q, Jiang L *et al.* Allelic heterogeneity at the serotonin transporter locus (SLC6A4) confers susceptibility to autism and rigid-compulsive behaviors. *American journal of medical genetics* 2005; **77**(2)**:** 265-279.

53. Devlin B, Cook E, Coon H, Dawson G, Grigorenko E, McMahon W *et al.* Autism and the serotonin transporter: the long and short of it. *Molecular psychiatry* 2005; **10**(12)**:** 1110-1116.

54. Cook E, Courchesne R, Lord C, Cox NJ, Yan S, Lincoln A *et al.* Evidence of linkage between the serotonin transporter and autistic disorder. *Molecular psychiatry* 1997; **2:** 247-250.

55. Wassink TH, Hazlett HC, Epping EA, Arndt S, Dager SR, Schellenberg GD *et al.* Cerebral cortical gray matter overgrowth and functional variation of the serotonin transporter gene in autism. *Arch Gen psychiatry* 2007; **64**(6)**:** 709-717.

56. Spence SJ, Cantor RM, Chung L, Kim S, Geschwind DH, Alarcon M. Stratification based on language-related endophenotypes in autism: attempt to replicate reported linkage. *Am J Med Genet B Neuropsychiatr Genet* 2006; **141B**(6)**:** 591-598.

57. Ylisaukko-oja T, Alarcon M, Cantor RM, Auranen M, Vanhala R, Kempas E *et al.* Search for autism loci by combined analysis of Autism Genetic Resource Exchange and Finnish families. *Ann Neurol* 2006; **59**(1)**:** 145-155.

58. Lauritsen MB, Als TD, Dahl HA, Flint T, Wang AG, Vang M *et al.* A genome-wide search for alleles and haplotypes associated with autism and related pervasive developmental disorders on the Faroe Islands. *Molecular psychiatry* 2005; **11**(1)**:** 37-46.

59. Yasuda Y, Hashimoto R, Yamamori H, Ohi K, Fukumoto M, Umeda-Yano S *et al.* Gene expression analysis in lymphoblasts derived from patients with autism spectrum disorder. *Mol Autism* 2011; **2:** 9.

60. Stettner GM, Shoukier M, Hoger C, Brockmann K, Auber B. Familial intellectual disability and autistic behavior caused by a small FMR2 gene deletion. *Am J Med Genet A* 2011; **155A**(8)**:** 2003-2007.

61. Mazzocco MMM, Myers GF, Hamner JL, Panoscha R, Shapiro BK, Reiss AL. The prevalence of the FMR1 and FMR2 mutations among preschool children with language delay. *J Pediatr* 1998; **132**(5)**:** 795-801.

62. Moore SJ, Strain L, Cole GF, Miedzybrodzka Z, Kelly KF, Dean JC. Fragile X syndrome with FMR1 and FMR2 deletion. *Journal of medical genetics* 1999; **36**(7)**:** 565-566.

63. Sahoo T, Theisen A, Marble M, Tervo R, Rosenfeld JA, Torchia BS *et al.* Microdeletion of Xq28 involving the AFF2 (FMR2) gene in two unrelated males with developmental delay. *Am J Med Genet A* 2011; **155A**(12)**:** 3110-3115.
